# Fifteen 4-(2-meth­oxy­phen­yl)piperazin-1-ium salts containing organic anions: supra­molecular assembly in zero, one, two and three dimensions

**DOI:** 10.1107/S2056989020014097

**Published:** 2020-10-30

**Authors:** Chayanna Harish Chinthal, Channappa N. Kavitha, Hemmige S. Yathirajan, Sabine Foro, Ravindranath S. Rathore, Christopher Glidewell

**Affiliations:** a Department of Studies in Chemistry, University of Mysore, Manasagangotri, Mysuru-570 006, India; b Department of Chemistry, Maharani’s Science College for Women, Mysuru-570 001, India; c Institute of Materials Science, Darmstadt University of Technology, Alarich-Weiss-Strasse 2, D-64287 Darmstadt, Germany; d Department of Bioinformatics, School of Earth, Biological and Environmental Sciences, Central University of South Bihar, Gaya 824236, India; e School of Chemistry, University of St Andrews, St Andrews, Fife KY16 9ST, UK

**Keywords:** synthesis, piperazines, crystal structure, mol­ecular conformation, absolute configuration, disorder, hydrogen bonding, supra­molecular assembly

## Abstract

Fifteen 4-(2-meth­oxy­phen­yl)piperazin-1-ium salts containing organic anions have been prepared and structurally characterized. In the isostructural 4-chloro­benzoate and 4-bromo­benzoate salts, C_11_H_17_N_2_O^+^·C_7_H_4_ClO_2_^−^ (I) and C_11_H_17_N_2_O^+^·C_7_H_4_BrO_2_^−^ (II), and the 4-iodo­benzoate salt C_11_H_17_N_2_O^+^·C_7_H_4_IO_2_^−^ (III), the ions are linked by N—H⋯O hydrogen bonds, forming centrosymmetric *R* ^4^_4_(12) four-ion aggregates; a similar aggregate is formed in the 2-chloro­benzoate salt (V), isomeric with (I). In the 2-fluoro­benzoate salt C_11_H_17_N_2_O^+^·C_7_H_4_FO_2_^−^ (IV), and the isomorphous pair of salts, the 2-bromo­benzoate (VI), isomeric with (II) and 2-iodo­benzoate (VII), isomeric with (III), N—H⋯O and C—H⋯π(arene) interactions link the components into three-dimensional arrays. Four-ion *R* ^4^_4_(12) aggregates are also found in the 2-methyl­benzoate, 4-amino­benzoate and 4-nitro­benzoate salts, C_11_H_17_N_2_O^+^·C_8_H_7_O_2_^−^ (VIII), C_11_H_17_N_2_O^+^·C_7_H_6_NO_2_^−^ (IX) and C_11_H_17_N_2_O^+^·C_7_H_4_NO_4_^−^ (X), but those in (IX) are linked into complex sheets by an additional N—H⋯O hydrogen bond. In the 3,5-dinitrobenzoate salt, C_11_H_17_N_2_O^+^·C_7_H_3_N_2_O_6_^−^·2H_2_O (XI), N—H⋯O and O—H⋯O hydrogen bonds link the components into a complex ribbon structure. In the picrate salt, C_11_H_17_N_2_O^+^·C_6_H_2_N_3_O_7_^−^ (XII), the four-ion aggregates are linked into chains of rings by C—H⋯O hydrogen bonds. In the hydrogen maleate salt, C_11_H_17_N_2_O^+^·C_4_H_3_O_4_^−^ (XIII), two- and three-centre hydrogen bonds link the ions into a ribbon structure while both anions contain very short but asymmetric O—H⋯O hydrogen bonds, having O⋯O distances of 2.4447 (16) and 2.4707 (17) Å. O—H⋯O Hydrogen bonds link the anions in the hydrogen fumarate salt (XIV), isomeric with (XIII), into chains that are linked into sheets *via* N—H⋯O hydrogen bonds. In the hydrogen (2*R*,3*R*)-tartrate salt, C_11_H_17_N_2_O^+^·C_4_H_5_O_6_^−^·1.698H_2_O (XV), the anions are linked into sheets by O—H⋯O hydrogen bonds. Comparisons are made with the structures of some related compounds.

## Abstract 2

Fifteen 4-(2-meth­oxy­phen­yl)piperazin-1-ium salts with organic anions exhibit a range of hydrogen-bonded supra­molecular assemblies in the form of finite aggregates, a chain of rings, ribbons, sheets and three-dimensional networks.

## Chemical context   

**Figure 1:**
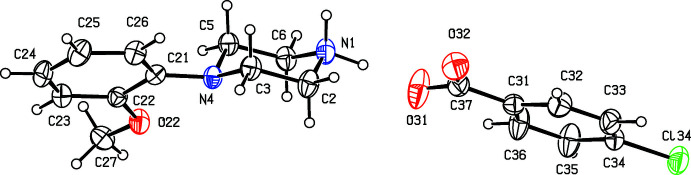
The independent components of compound (I) showing the atom-labelling scheme. Displacement ellipsoids are drawn at the 30% probability level.

We have recently reported the mol­ecular and supra­molecular structures of the recreational drug *N*-(4-meth­oxy­phen­yl)piperazine (4-MeOPP) (Kiran Kumar *et al.*, 2020 ▸) and those of a range of salts formed by 4-MeOPP with organic acids (Kiran Kumar, Yathirajan, Foro *et al.*, 2019 ▸; Kiran Kumar *et al.* 2020 ▸), as well as those of a number of *N*-aroyl derivatives (Kiran Kumar, Yathirajan, Sagar *et al.*, 2019 ▸). We have also reported the structures of some salts of *N*-(4-fluoro­phen­yl)piperazine (4-FPP) (Harish Chinthal, Yathirajan, Archana *et al.*, 2020 ▸; Harish Chinthal, Yathirajan, Kavitha *et al.*, 2020 ▸). As a continuation of this study, we have now investigated a number of salts of the isomeric *N*-(2-meth­oxy­phen­yl)piperazine (2-MeOPP), which has been used as a building block in the synthesis of both 5-HT_1A_ receptor ligands (Orjales *et al.*, 1995 ▸) and dopamine D_2_ and D_3_ ligands (Hackling *et al.*, 2003 ▸) and also as a building block for the synthesis of derivatives exhibiting anti­depressant-like activity (Waszkielewicz *et al.*, 2015 ▸). Here we report the syntheses and structures of the salts (I)–(XI) (Figs. 1 ▸–11 ▸ ▸ ▸ ▸ ▸ ▸ ▸ ▸ ▸ ▸) formed between 2-MeOPP and eleven aromatic carb­oxy­lic acids, along with a redetermination of the salt (XII) (Fig. 12 ▸) formed with 2,4,6-tri­nitro­phenol (picric acid) where the reported structure (Verdonk *et al.*, 1997 ▸; CSD refcode NEBGIK) shows signs of unmodelled disorder, and we report here also the structures of three acid salts (XIII)–(XV) (Figs. 13 ▸–15 ▸ ▸) formed with some aliphatic di­carb­oxy­lic acids. All of the salts (I)–(XV) were straightforwardly prepared by the acid–base reactions and subsequent crystallizations of equimolar mixtures of 2-MeOPP with the appropriate organic acid.
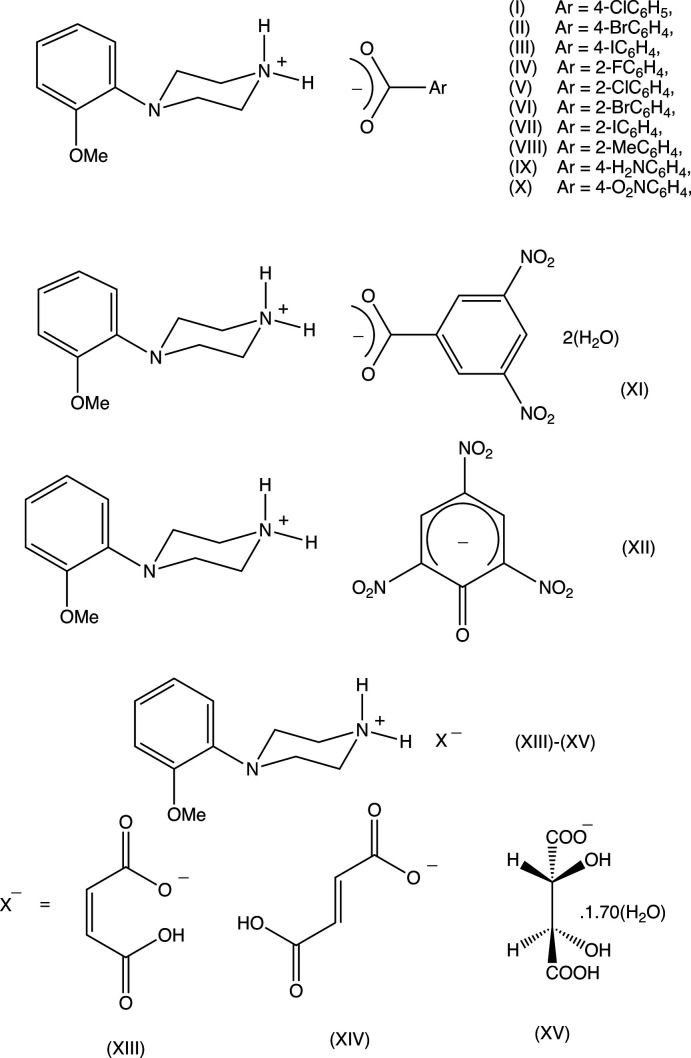


## Structural commentary   

**Figure 3:**
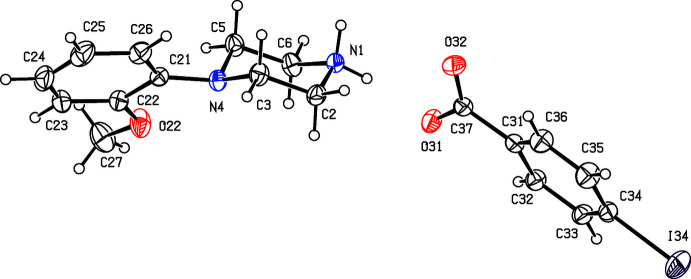
The independent components of compound (III) showing the atom-labelling scheme. Displacement ellipsoids are drawn at the 30% probability level.

**Figure 4:**
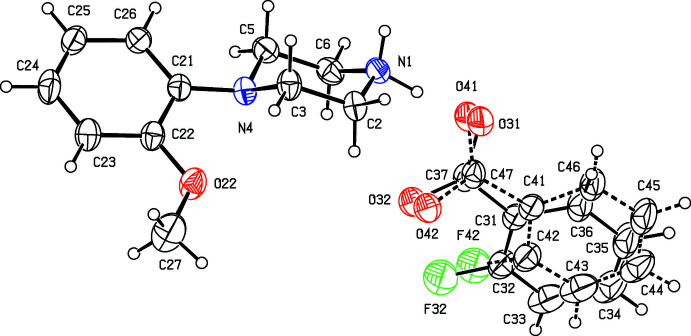
The independent components of compound (IV) showing the atom-labelling scheme and the disorder in the anion; the major disorder component is drawn using full lines and the minor disorder component is drawn using broken lines. Displacement ellipsoids are drawn at the 30% probability level.

**Figure 6:**
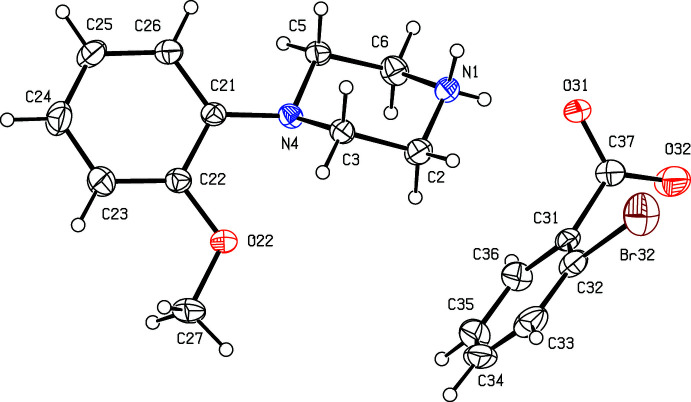
The independent components of compound (VI) showing the atom-labelling scheme. Displacement ellipsoids are drawn at the 30% probability level.

**Figure 7:**
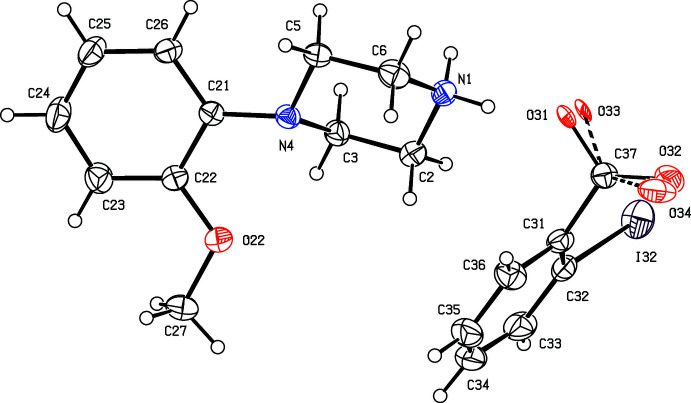
The independent components of compound (VII) showing the atom-labelling scheme and the disorder in the carboxyl­ate group; the major disorder component is drawn using full lines and the minor disorder component is drawn using broken lines. Displacement ellipsoids are drawn at the 30% probability level.

**Figure 8:**
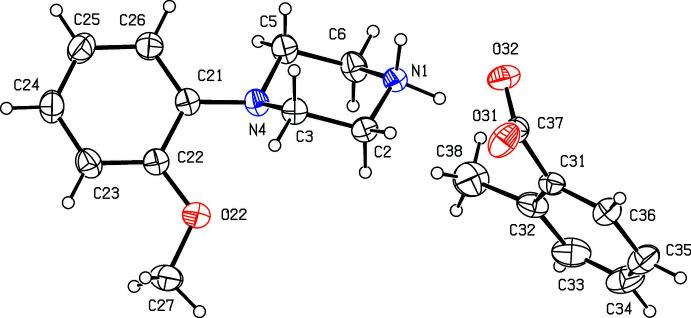
The independent components of compound (VIII) showing the atom-labelling scheme. Displacement ellipsoids are drawn at the 30% probability level.

**Figure 11:**
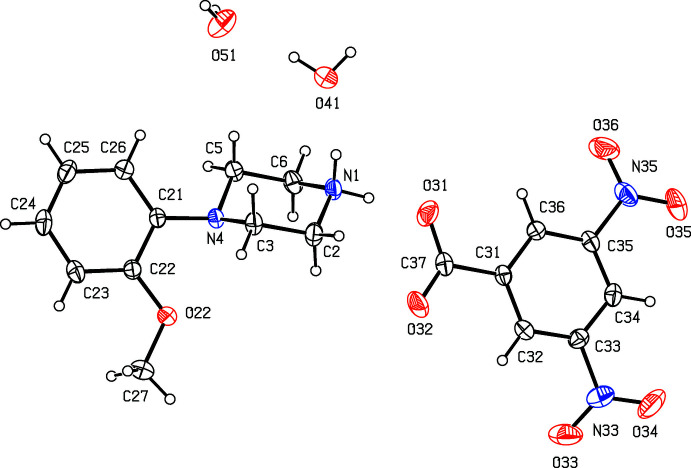
The independent components of compound (XI) showing the atom-labelling scheme. Displacement ellipsoids are drawn at the 30% probability level.

**Figure 12:**
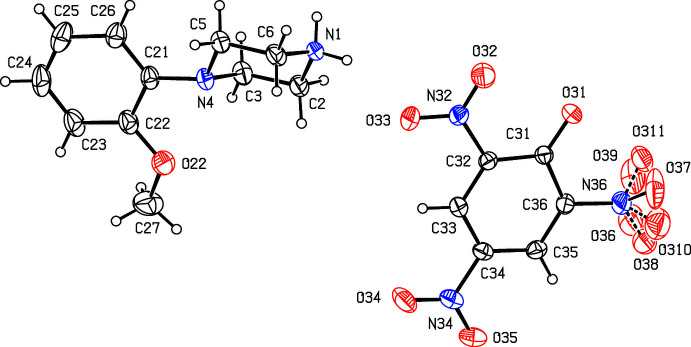
The independent components of compound (XII) showing the atom-labelling scheme and the disorder in one of the nitro groups, where the dominant disorder component is drawn using full lines, and the two minor disorder components are drawn using broken lines. Displacement ellipsoids are drawn at the 30% probability level.

Compounds (I) and (II) (Figs. 1 ▸ and 2 ▸) are isostructural in space group *P* 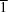. Although the 4-iodo­benzoate analogue (III) (Fig. 3 ▸) also crystallizes in the same space group, it is not isostructural with (I) and (II). Among the 2-halobenzoate salts, in the 2-fluoro­benzoate (IV) the anion is disordered over two sets of atomic sites having occupancies 0.907 (8) and 0.093 (8) (Fig. 4 ▸). There is a significant peak, 1.15 e Å^−3^, in the final difference map for compound (V): it was originally thought that this might represent a partial-occupancy water mol­ecule, although no associated H atoms could be located, but its distance from atom O32 is only 2.35 Å, which would require an unusually short O—H⋯O hydrogen bond for this assignment to be plausible. Consistent with this, examination of the refined, solvent-free structure of (V) using *PLATON* (Spek, 2020 ▸) showed that the structure contains no solvent-accessible void spaces. Compounds (VI) and (VII) are isomorphous, but whereas the components of (VI) are fully ordered (Fig. 6 ▸), in (VII) the carboxyl­ate group in the anion is disordered over two sets of atomic sites having occupancies 0.54 (9) and 0.46 (9) (Fig. 7 ▸); hence, these isomorphous compounds cannot be regarded as strictly isostructural (*cf*. Acosta *et al.*, 2009 ▸; Yépes *et al.*, 2012 ▸; Shreekanth *et al.*, 2020 ▸), because of the disorder in (VII). The structures of (VI) and (VII) are mutually inverse for the crystals selected for data collection, but this has no chemical significance. Compounds (VIII)–(X) (Figs. 8 ▸–10 ▸ ▸) all crystallize in solvent-free form, but the 3,5-di­nitro­benzoate salt (XI) is a dihydrate (Fig. 11 ▸). The structure of the picrate salt (XII) was reported a number of years ago (Verdonk *et al.*, 1997 ▸), but the deposited anisotropic displacement parameters suggest the presence of unmodelled disorder in one of the nitro groups. Accordingly, we have redetermined this structure and found, indeed, that one of the nitro groups is disordered over three sets of atomic sites having occupancies 0.850 (5), 0.080 (4) and 0.069 (4) (Fig. 12 ▸).

**Figure 13:**
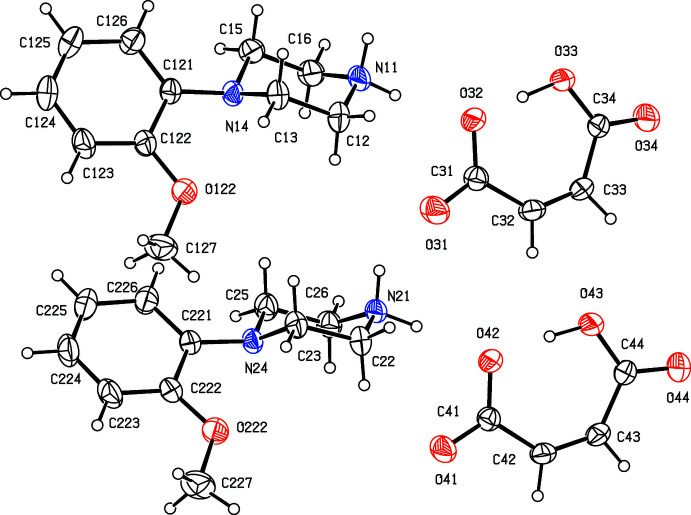
The independent components of compound (XIII) showing the atom-labelling scheme. Displacement ellipsoids are drawn at the 30% probability level.

**Figure 14:**
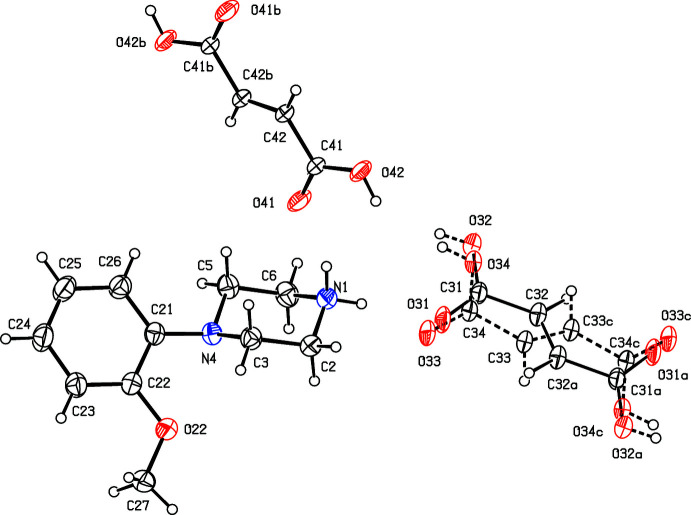
The independent components of compound (XIV) showing the atom-labelling scheme and the disorder in one of the anions. The major disorder component is drawn using full lines and the minor disorder component is drawn using broken lines. Displacement ellipsoids are drawn at the 30% probability level. The atoms marked ‘a’ or ‘b’ are at the symmetry positions (2 − *x*, 1 − *y*, 2 − *z*) and (−*x*, −*y*, 2 − *z*), respectively. The H atoms bonded to atoms O32, O34 and O42 have occupancies 0.286 (9), 0.214 (9) and 0.5, respectively, as do their inversion-related equivalents.

**Figure 15:**
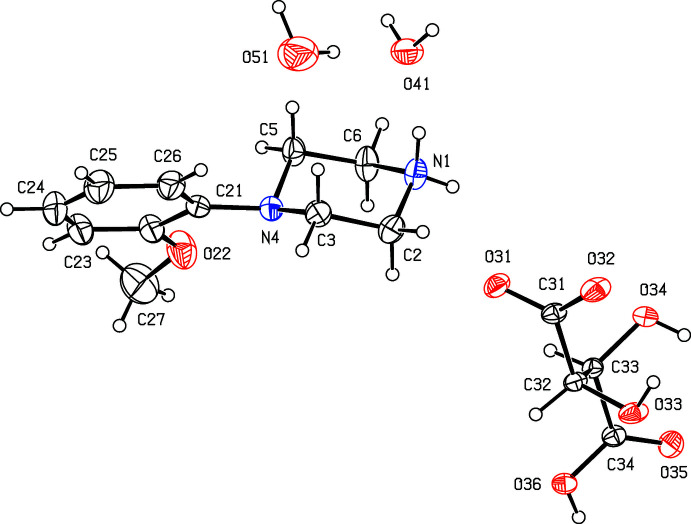
The independent components of compound (XV) showing the atom-labelling scheme. Displacement ellipsoids are drawn at the 30% probability level.

The solvent-free 1:1 acid salt (XIII) derived from maleic acid crystallizes with *Z*′ = 2 (Fig. 13 ▸). A search for possible additional crystallographic symmetry revealed none, although the atomic coordinates of the two cations and the two anions are related by the approximate, but non-crystallographic translation (*x*, 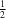 + *y*, *z*). In sharp contrast to compound (XIII), the 1:1 salt (XIV) derived from fumaric acid, which is isomeric with maleic acid, crystallizes with two independent hydrogen fumarate anions, each lying across a centre of inversion: one of the anions is fully ordered but the other is disordered over two sets of atomic sites having occupancies 0.572 (9) and 0.428 (9) (Fig. 14 ▸). The 1:1 acid salt (XV) derived from (2*R*,3*R*)-tartaric acid crystallizes as a dihydrate (Fig. 15 ▸).

In none of the salts reported does the cation exhibit any inter­nal symmetry: hence all are conformationally chiral but, with the exception of compounds (VI) and (VII), the space groups indicate that equal numbers of both conformational enanti­omers are present. For all compounds except (VII), the reference cation was selected to be one for which the ring-puckering angles θ (Cremer & Pople, 1975 ▸) is close to zero, as calculated for the atom sequence (N1,C2,C3,N4,C5,C6). For the crystal of (VII) chosen for data collection, the value of this angle is 177.2 (5)°, confirming that this salt and (VI) have opposite absolute structures. In all of the cations, the piperazine ring adopts a chair conformation with the *N*-aryl substituent in an equatorial site. In the 2-meth­oxy­phenyl units, the meth­oxy C atom is always close to coplanar with the adjacent aryl ring: the displacement of this atom from the plane of the ring ranges from 0.038 (5) Å in compound (I) to 0.288 (5) Å in compound (VII). Associated with this near planarity, the two exocyclic C—C—O angles differ in each compound by *ca* 10°, as is usually observed in planar or near-planar alk­oxy­arenes (Seip & Seip, 1973 ▸; Ferguson *et al.*, 1996 ▸).

The two independent ions in compound (XIII) both contain a very short O—H⋯O hydrogen bond (Table 1 ▸): while these are both nearly linear, the two O—H distances in each are significantly different, as established both by refinement of the atomic coordinates for the H atom, and from the final difference maps.

## Supra­molecular features   

The supra­molecular assembly in the salts (I)–(XV) is based on N—H⋯O and O—H⋯O hydrogen bonds augmented in a number of cases by C—H⋯O and C—H⋯π(arene) hydrogen bonds. In general, we have discounted hydrogen bonds having *D*—H⋯*A* angles that are significantly less than 140°, as the inter­action energies associated with such contacts are likely to be very low, so that these cannot be regarded as structurally significant (Wood *et al.*, 2009 ▸). We have also discounted short contacts involving the H atoms of the methyl groups, as such groups are likely to be undergoing very rapid rotation about the adjacent C—O bonds (Riddell & Rogerson, 1996 ▸, 1997 ▸). Most of the C—H⋯π(arene) contacts have H⋯*Cg* distances in excess of 2.85 Å, and we have therefore only considered the effects of such contacts in the assembly of compounds (III) and (IV), where these distances are below 2.80 Å. It should perhaps be conceded here that these are somewhat arbitrary judgments, made with the primary aim of avoiding over-inter­pretation of the longer contacts and over-complication of the crystal structure descriptions.

**Figure 16:**
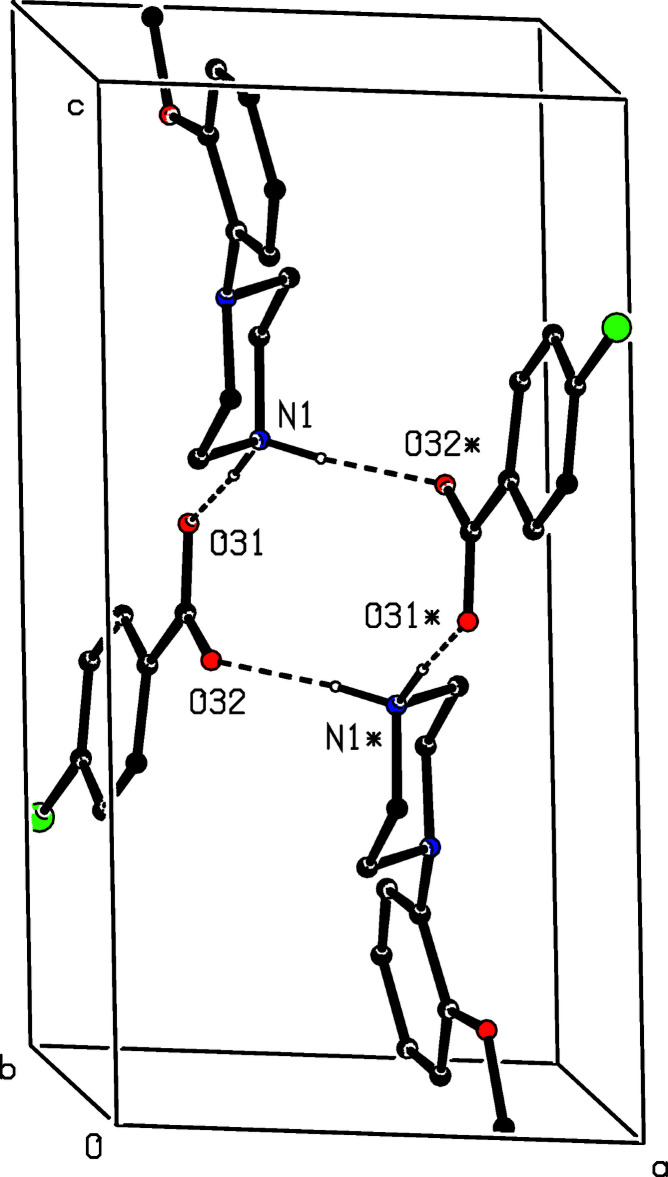
Part of the crystal structure of compound (I) showing the formation of a centrosymmetric four-ion aggregate. Hydrogen bonds are drawn as dashed lines and, for the sake of clarity, the H atoms bonded to C atoms have been omitted. The atoms marked with an asterisk (*) are at the symmetry position (1 − *x*, 1 − *y*, 1 − *z*).

**Figure 17:**
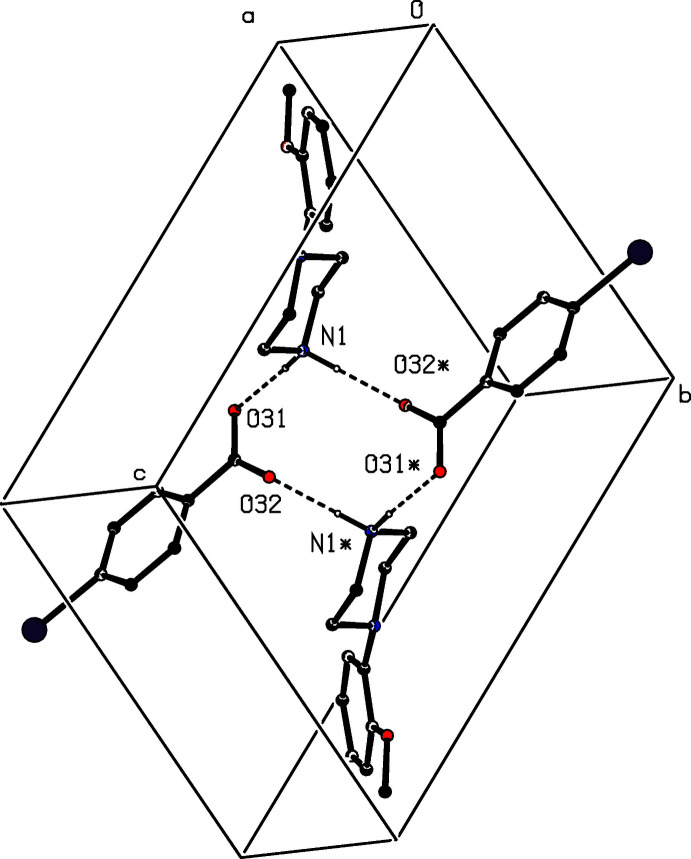
Part of the crystal structure of compound (III) showing the formation of a centrosymmetric four-ion aggregate. Hydrogen bonds are drawn as dashed lines and, for the sake of clarity, the H atoms bonded to C atoms have been omitted. The atoms marked with an asterisk (*) are at the symmetry position (1 − *x*, 1 − *y*, 1 − *z*).

In each of the isostructural pair of compounds (I) and (II), two N—H⋯O hydrogen bonds (Table 1 ▸) link the ionic components into a centrosymmetric four-ion aggregate, characterized by an 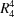
(12) (Etter, 1990 ▸; Etter *et al.*, 1990 ▸; Bernstein *et al.*, 1995 ▸) motif (Fig. 16 ▸). A similar motif occurs in the structure of compound (III) (Fig. 17 ▸), but the different orientations of the unit-cell outline in Figs. 16 ▸ and 17 ▸, illustrate the different arrangements of the components in compounds (I) and (II) on the one hand and compound (III) on the other. In (III), the four-ion aggregates are linked into chains by a C—H⋯π(arene) inter­action, but the C—H⋯O contact in (III) has a very small *D*—H⋯*A* angle and is thus not structurally significant (Wood *et al.*, 2009 ▸).

**Figure 18:**
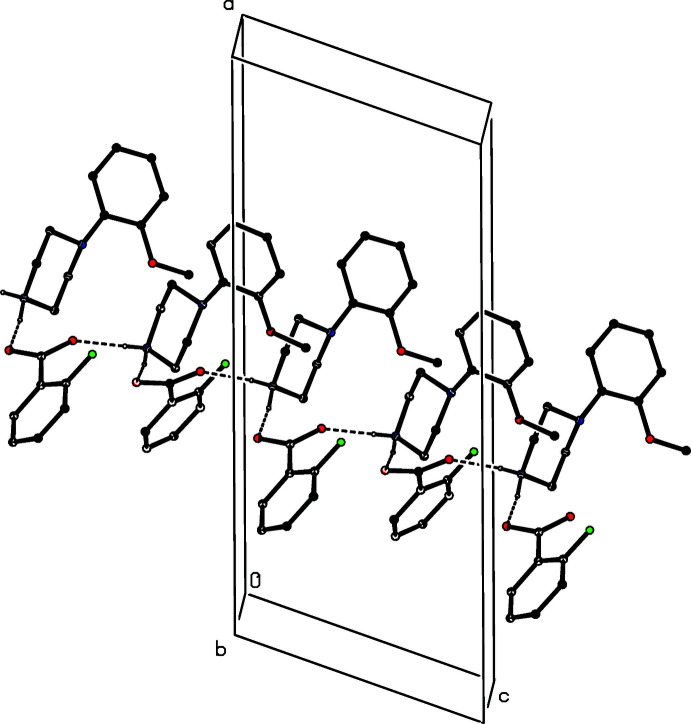
Part of the crystal structure of compound (IV) showing the linking of the ion pairs by a further N—H⋯O hydrogen bond to form a 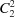
(6) chain running parallel to [001]. Hydrogen bonds are drawn as dashed lines and, for the sake of clarity, the minor disorder component and the H atoms bonded to C atoms have been omitted.

**Figure 19:**
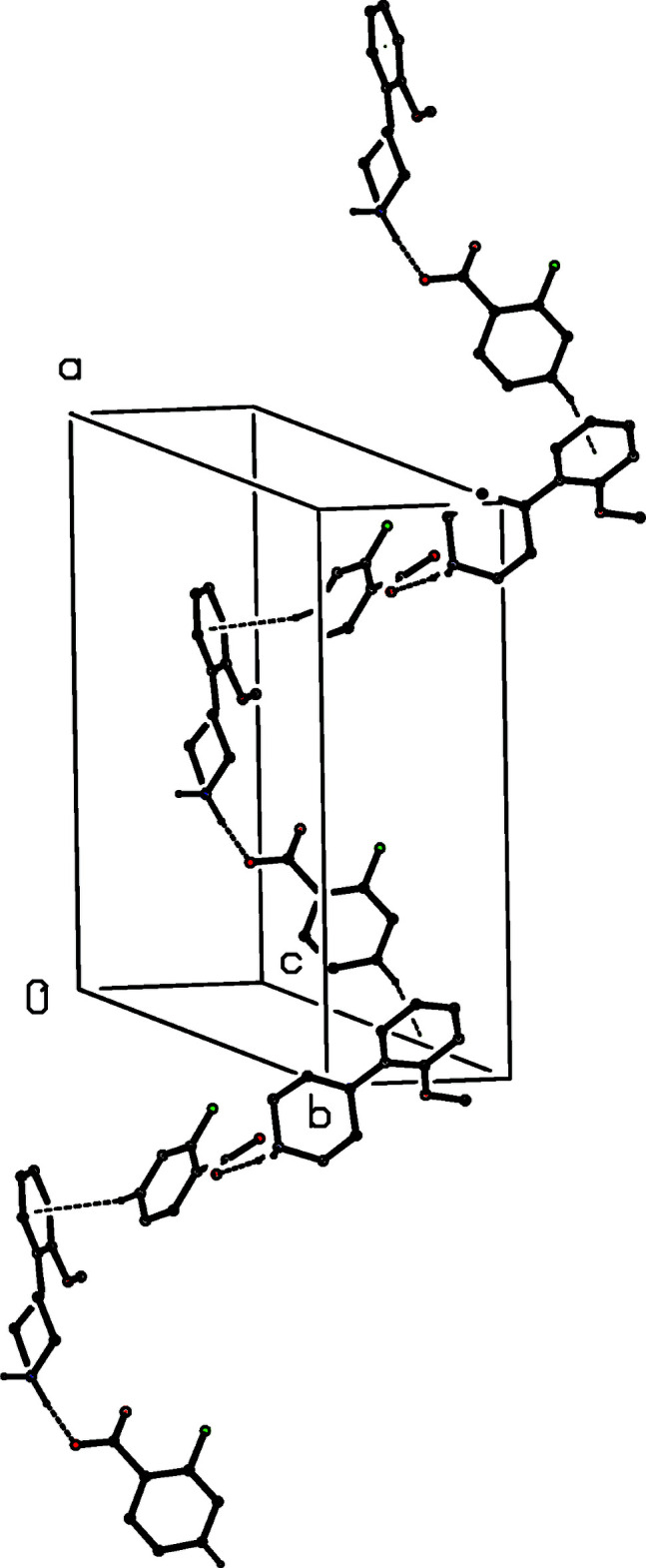
Part of the crystal structure of compound (IV) showing the linking of the ions pairs by a C—H⋯π(arene) hydrogen bond to form a chain parallel to [101]. Hydrogen bonds are drawn as dashed lines and, for the sake of clarity, the minor disorder component and the H atoms not involved in the motif shown have been omitted.

**Figure 20:**
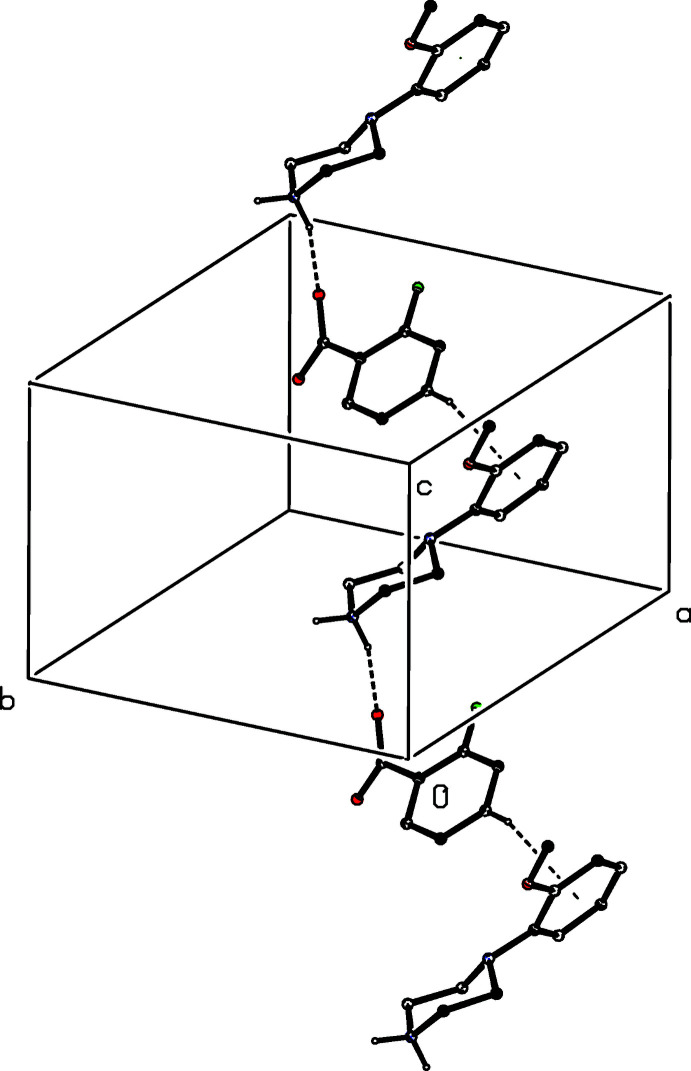
Part of the crystal structure of compound (IV) showing the alternating action of N—H⋯O and C—H⋯π(arene) hydrogen bonds in linking the ion pairs into a chain parallel to [112]. Hydrogen bonds are drawn as dashed lines and, for the sake of clarity, the minor disorder component and the H atoms not involved in the motif shown have been omitted.

The hydrogen bonding involving the two disorder components in compound (IV) are very similar (Table 1 ▸) and thus only the major component needs to be considered here. The combination of two N—H⋯O hydrogen bonds and one C—H⋯π(arene) hydrogen bond, involving atom C34 as the donor, links the ions into a three-dimensional network, whose formation is readily analysed in terms of three one-dimensional sub-structures (Ferguson *et al.*, 1998*a* ▸,*b* ▸; Gregson *et al.*, 2000 ▸). In addition to the N—H⋯O hydrogen bond forming the ion pair, which defines the selected asymmetric unit, we consider in turn the linking of these ion pairs by the action of the N—H⋯O hydrogen bond involving atom H12, acting alone; by that of the C—H⋯π(arene) hydrogen bond acting alone; and finally by that of the two hydrogen bonds in combination. The ion pairs are linked by a second N—H⋯O hydrogen bond to form a 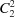
(6) chain running parallel to the [001] direction (Fig. 18 ▸), and they are linked by the C—H⋯π(arene) hydrogen bond to form a chain running parallel to [101] (Fig. 19 ▸). The N—H⋯O and C—H⋯π hydrogen bonds, acting alternately, generate a chain running parallel to the [112] direction (Fig. 20 ▸), and the combination of chains running parallel to [001], [101] and [112] suffices to generate a three-dimensional structure. In the 2-chloro­benzoate analogue, compound (V), two independent N-H⋯O hydrogen bonds again link the ions into a centrosymmetric 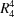
(12) motif, of the type observed in compounds (I)–(III). There are two C—H⋯π(arene) contacts in (V), but these are both long, and probably not structurally significant.

**Figure 21:**
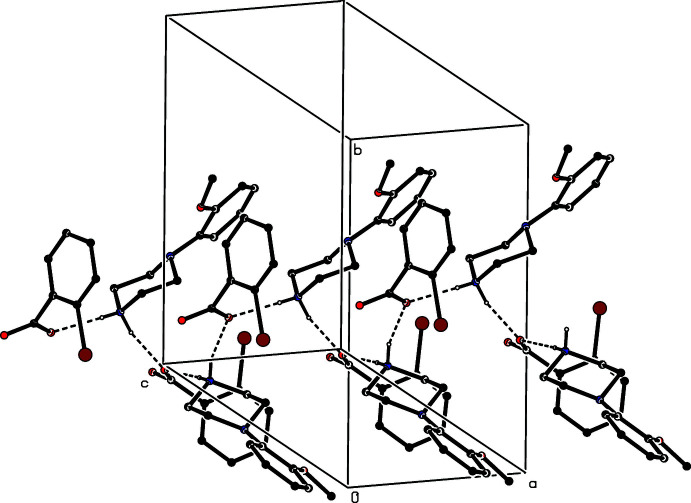
Part of the crystal structure of compound (VI) showing the formation of a 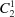
(4) chain running parallel to [100], in which ion pairs are linked by a further N—H⋯O hydrogen bond. Hydrogen bonds are drawn as dashed lines and, for the sake of clarity, the H atoms bonded to C atoms have been omitted.

**Figure 22:**
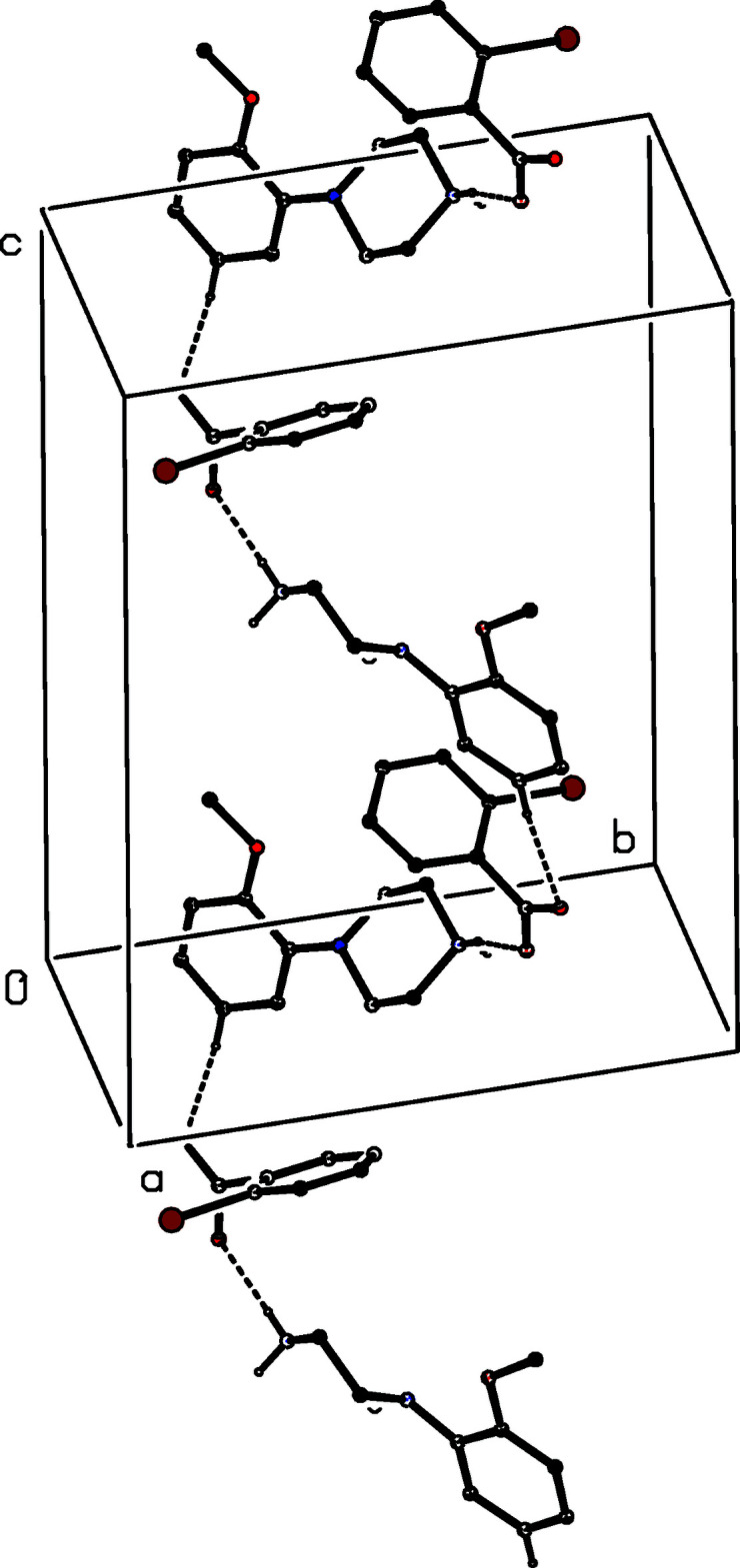
Part of the crystal structure of compound (VI) showing the formation of a 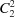
(12) chain running parallel to [001], in which ion pairs are linked by a C—H⋯O hydrogen bond. Hydrogen bonds are drawn as dashed lines and, for the sake of clarity, the H atoms not involved in the motif shown have been omitted.

**Figure 23:**
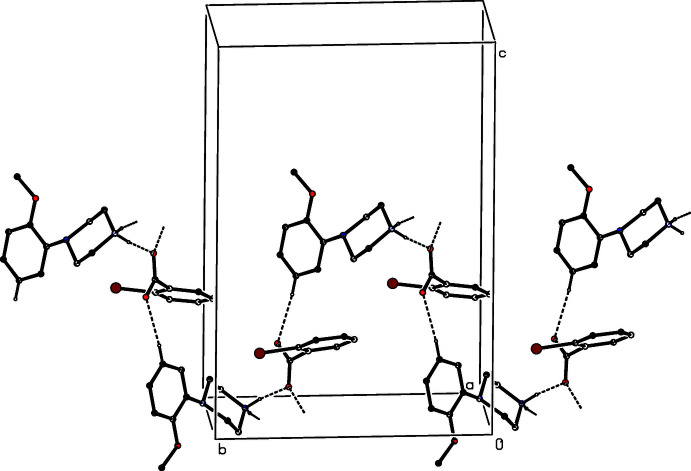
Part of the crystal structure of compound (VI) showing the formation of a chain running parallel to [010], in which ion pairs are linked by alternating N—H⋯O and C—H⋯O hydrogen bonds. Hydrogen bonds are drawn as dashed lines and, for the sake of clarity, the H atoms not involved in the motif shown have been omitted.

The ion pairs in compounds (VI) and (VII) are again linked into three-dimensional arrays, by a combination of N—H⋯O and C—H⋯O hydrogen bonds, as opposed to the N—H⋯O and C—H⋯π(arene) inter­actions in the structure of (IV). An N—H⋯O hydrogen bond links ion pairs which are related by the 2_1_ screw axis along (*x*, 1/4, 1/2) to form a 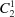
(4) chain along [100] (Fig. 21 ▸). In addition, the ion pairs which are related by the 2_1_ screw axis along (1/4, 1/2, *z*) are linked by a C—H⋯O hydrogen bond to form a 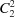
(12) chain along [001] (Fig. 22 ▸), while the alternating action of the N—H⋯O and C—H⋯O hydrogen bonds generates a chain running parallel to the [010] direction (Fig. 23 ▸). The combination of chains along [100], [010] and [001] thus generates a three-dimensional array.

**Figure 24:**
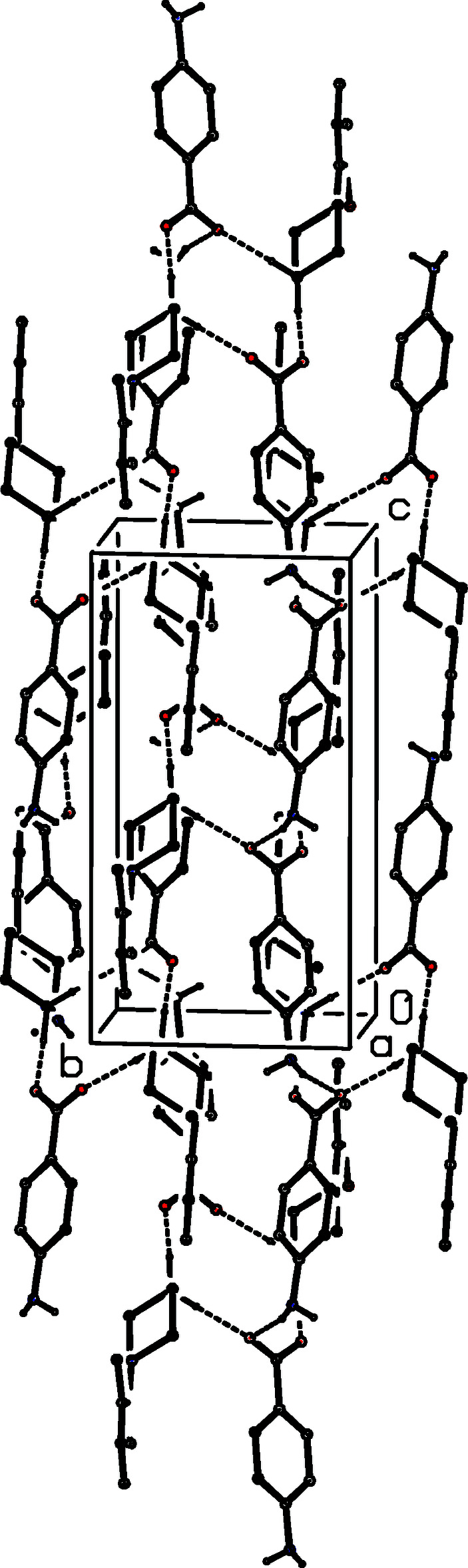
Part of the crystal structure of compound (IX) showing the formation of a hydrogen-bonded sheet lying parallel to (100). Hydrogen bonds are drawn as dashed lines and, for the sake of clarity, the H atoms bonded to C atoms have been omitted.

**Figure 25:**
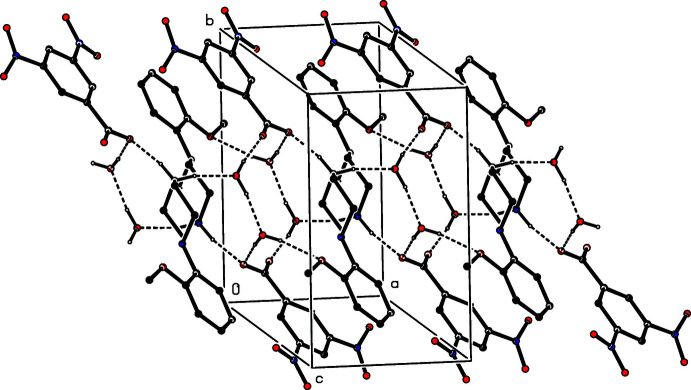
Part of the crystal structure of compound (XI) showing the formation of a hydrogen-bonded ribbon running parallel to [100]. Hydrogen bonds are drawn as dashed lines and, for the sake of clarity, the H atoms bonded to C atoms have been omitted.

**Figure 26:**
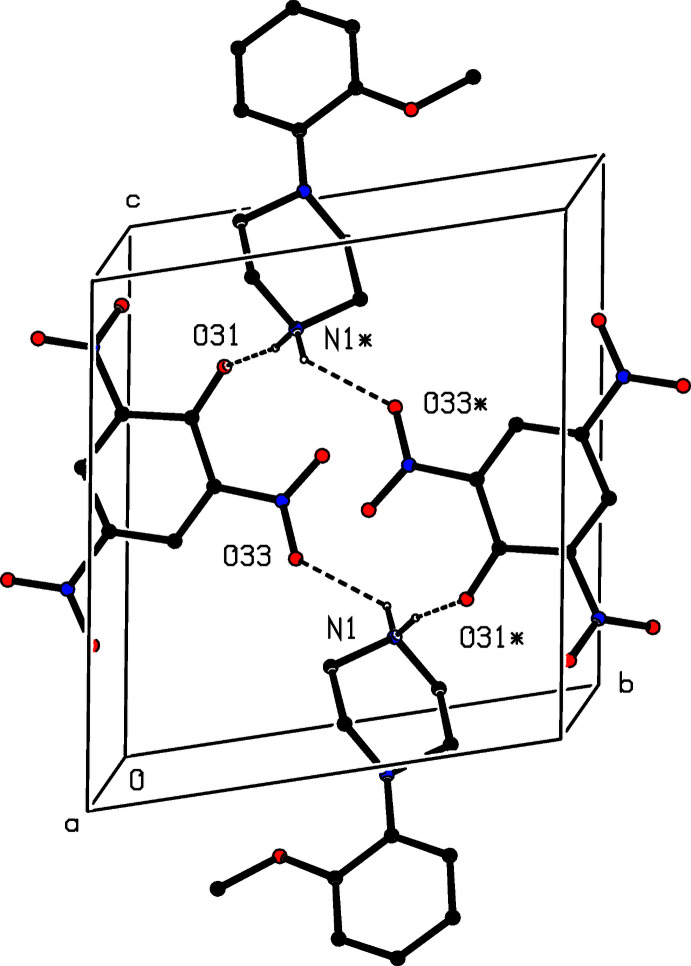
Part of the crystal structure of compound (XII) showing the formation of a centrosymmetric four-ion aggregate. Hydrogen bonds are drawn as dashed lines and, for the sake of clarity, the H atoms bonded to C atoms have been omitted and only the major disorder component is shown. The atoms marked with an asterisk (*) are at the symmetry position (1 − *x*, 1 − *y*, 1 − *z*).

The ions in compound (VIII) are linked by two N—H⋯O hydrogen bonds to form an 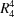
(12) four-ion aggregate analogous to those observed in compounds (I)–(III) and (V). Similar four-ion aggregates are also found in compounds (IX) and (X), but in (IX) they are linked by a further N—H⋯O hydrogen bond, involving the amino group, to form a complex sheet lying parallel to (100) (Fig. 24 ▸). In the dihydrate (XI), each water mol­ecule acts as a single acceptor and a double donor of hydrogen bonds (Table 1 ▸), and supra­molecular aggregation takes the form of a complex ribbon running parallel to the [100] direction (Fig. 25 ▸). In the picrate salt (XII), a combination of two independent N—H⋯O hydrogen bonds links the components into a centrosymmetric four-ion aggregate of 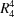
(16) type, where the two acceptor are the phenolic atom O31 and one of the nitro O atoms (Fig. 26 ▸). Aggregates of this type are weakly linked into a chain of rings by a C—H⋯O hydrogen bond.

**Figure 27:**
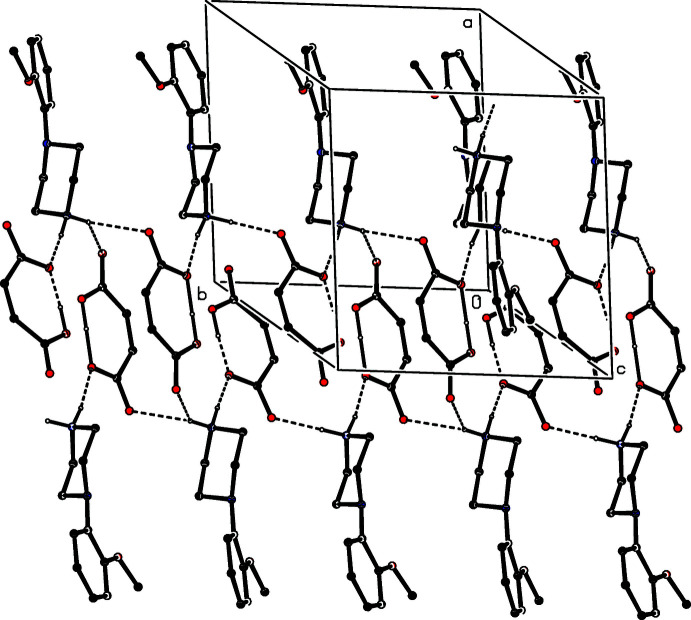
Part of the crystal structure of compound (XIII) showing the formation of a hydrogen-bonded ribbon of 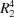
(14) and 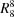
(30) rings running parallel to [010]. Hydrogen bonds are drawn as dashed lines and, for the sake of clarity, the H atoms bonded to C atoms have been omitted.

**Figure 28:**
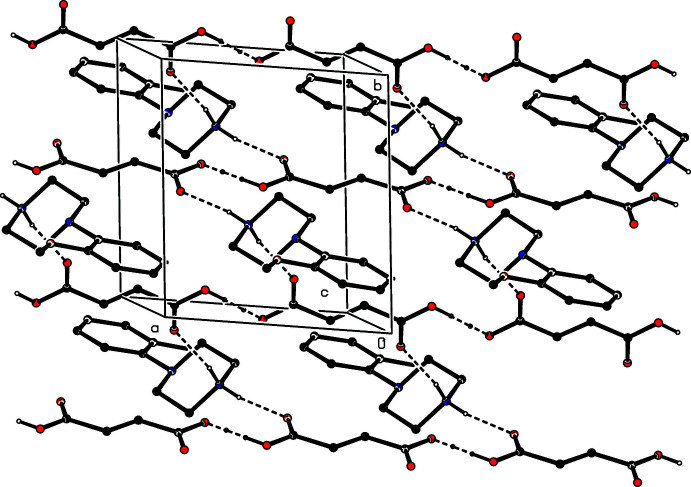
Part of the crystal structure of compound (XIV) showing the formation of a hydrogen-bonded sheet of 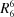
(26) rings lying parallel to [001]. Hydrogen bonds are drawn as dashed lines and, for the sake of clarity, the H atoms bonded to C atoms have been omitted.

**Figure 29:**
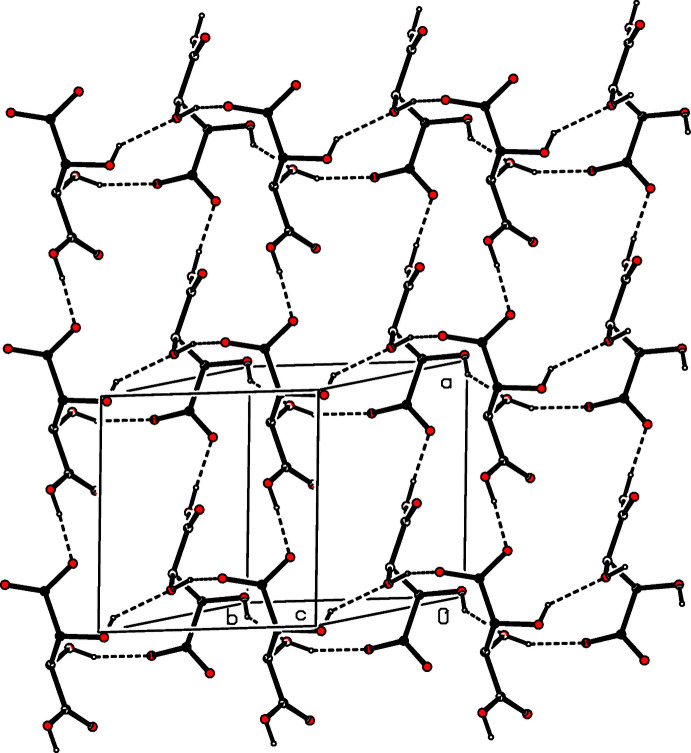
Part of the crystal structure of compound (XV) showing the formation of a hydrogen-bonded sheet of anions lying parallel to (001). Hydrogen bonds are drawn as dashed lines and, for the sake of clarity, the H atoms bonded to C atoms have been omitted.

In compound (XIII), where *Z*′ = 2, each of the anions contains a very short O—H⋯O hydrogen bond, although in each of these inter­actions the two O—H distances are significantly different (Table 1 ▸). The supra­molecular assembly depends upon three independent two-centre N—H⋯O hydrogen bonds and one three-centre N—H⋯(O)_2_ hydrogen bond. These link the ions into a ribbon, or mol­ecular ladder, running parallel to the [010] direction and in which 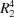
(14) rings centred at (0, *n* + 1/2, 1/2) alternate with 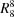
(30) rings centred at (0, *n*, 1/2), where *n* represents an integer in each case (Fig. 27 ▸). Analysis of the supra­molecular assembly in compound (XIV) is complicated by the combination of centrosymmetric anions and the disorder exhibited by one of them. However, since the hydrogen bonds involving the two disorder components are very similar, only the major disorder components need to be considered here. The ordered anions are linked by O—H⋯O hydrogen bonds into a chain along (*x*, 0, 1) and the disordered anions are similarly linked into a chain along (*x*, 1/2, 1). The two types of chain, which alternate along the [010] direction, are linked by the cations to form a sheet of 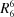
(26) rings lying parallel to (001) (Fig. 28 ▸). In the structure of compound (XV), the anions are linked by three independent O—H⋯O hydrogen bonds, in which both of the hydroxyl groups as well as the carboxyl group act as donors, to form a sheet lying parallel to (001), in which both 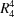
(18) and 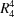
(20) rings can be identified (Fig. 29 ▸). The cations and the water mol­ecules are tethered to this sheet, markedly increasing its complexity but without changing the dimensionality of the overall assembly. The result is a thick tripartite sheet, occupying the whole domain 0 < *z* < 1.0 and having a hydrogen-bonded layer in the centre with the aryl groups on the outside surfaces: there are no direction-specific inter­actions between adjacent sheets.

In summary, therefore, the hydrogen-bonded assembly is finite, or zero-dimensional in compounds (I)–(III), (V), (VIII) and (X); one-dimensional in (XI), (XII) and (XIII); two-dimensional in (IX), (XIV) and (XV); and three–dimensional in (IV), (VI) and (VII).

## Database survey   

It is of inter­est briefly to compare the structures of the compounds reported here with those of some closely related examples, in particular the salts formed by the isomeric *N*-(4-meth­oxy­phen­yl)piperazine (4-MeOPP) and the analogous *N*-(4-fluoro­phen­yl)piperazine (4-FPP). The salts formed between 4-MeOPP and the benzoic acids 4-*X*C_6_H_4_COOH, where *X* = H, F, Cl, and Br, all crystallize as stoichiometric monohydrates and they are all isomorphous in space group *P* 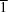 (Kiran Kumar, Yathirajan, Foro *et al.*, 2019 ▸), a combination of N—H⋯O, O—H⋯O, C—H⋯O and C—H⋯π(arene) hydrogen bonds links the components into complex sheets. By contrast, compounds (I)–(III) reported here all crystallize in solvent-free form and all form finite centrosymmetric four-ion aggregates (Figs. 16 ▸ and 17 ▸). The salt formed between 4-MeOPP and 4-amino­benzoate crystallizes as a monohydrate (Kiran Kumar *et al.*, 2020 ▸), as compared with the solvent free analogues (IX) reported here, and the components are linked by a combination of N—H⋯O, O—H⋯O and C—H⋯π(arene) hydrogen bonds to form a three-dimensional assembly, as compared with the two-dimensional assembly in (IX). The 3,5-di­nitro­benzoate salt with 4-MeOPP crystallizes in solvent-free form (Kiran Kumar *et al.*, 2020 ▸), as opposed to the dihydrate (XI) reported here, and the component ions are linked into the simple 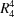
(12) motif found here for compounds (I)–(III), (VIII) and (X). The picrate salt of 4-MeOPP exhibits orientational disorder in one of the nitro groups (Kiran Kumar *et al.*, 2020 ▸), as observed in compound (XII) here, but the supra­molecular aggregation is more complex than the simple aggregate found for (XII), in that a combination of N—H⋯O and C—H⋯π(arene) hydrogen bonds generates a sheet structure. The anion in the hydrogen maleate salt of 4-MeOPP, which crystallizes with *Z*′ = 1 (Kiran Kumar, Yathirajan, Foro *et al.*, 2019 ▸) unlike the *Z*′ = 2 for compound (XIII), contains a very short, but unsymmetrical O—H⋯O hydrogen bond, and the ions are linked into a chain of rings by a combination of two-centre N—H⋯O and three-centre N—H⋯(O,O) hydrogen bonds. By contrast with compound (XIV) reported here where there are two independent hydrogen fumarate anions each lying across a centre of inversion, in the hydrogen fumarate salt of 4-MeOPP, there is only one type of anion, although this exhibits some orientational disorder and *Z*′ = 1: a combination of N—H⋯O and O—H⋯O and C—H⋯π(arene) hydrogen bonds links the ions into a three-dimensional structure, as opposed to the two-dimensional structure of (XIV). Finally, we note some salts formed by 4-FPP with organic acids (Harish Chinthal, Yathirajan, Archana *et al.*, 2020 ▸; Harish Chinthal, Yathirajan, Kavitha *et al.*, 2020 ▸). The 2-fluoro­benzoate crystallizes as a stoichiometric monohydrate, and the 2-bromo­benzoate as a partial hydrate, while the 2-iodo­benzoate crystallizes in solvent-free form (Harish Chinthal, Yathirajan, Kavitha *et al.*, 2020 ▸), in contrast to compounds (IV)–(VII), which are all solvent-free, and the 3,5-di­nitro­benzoate salt of 4-FPP is also solvent-free, as opposed to the dihydrate (XI). The 1:1 acid salt formed between (2*R*,3*R*)-tartaric acid and 4-FPP crystallizes as a monohydrate (Harish Chinthal, Yathirajan, Archana *et al.*, 2020 ▸), whereas the analogous compound (XV) crystallizes as a 1.70 (hydrate).

## Synthesis and crystallization   

All reagents were obtained commercially, and all were used as received. For the synthesis of compounds (I)–(XV), solutions of *N*-(2-meth­oxy­phen­yl)piperazine (100 mg, 0.52 mmol) in methanol (10 ml) were mixed with an equimolar qu­antity of the appropriate acid [4-chloro­benzoic acid (82 mg) for (I), 4-bromo­benzoic acid (103 mg) for (II), 4-iodo­benzoic acid (129 mg) for (III), 2-fluoro­benzoic acid (73 mg) for (IV), 2-chloro­benzoic acid (82 mg) for (V), 2-bromo­benzoic acid (103 mg) for (VI), 2-iodo­benzoic acid (129 mg) for (VII), 2-methyl­benzoic acid (71 mg) for (VIII), 4-amino­benzoic acid (72 mg) for (IX), 4-nitro­benzoic acid (97 mg) for (X), 3,5-di­nitro­benzoic acid (110 mg) for (XI), picric acid (120 mg) for (XII), maleic acid (61 mg) for (XIII), fumaric acid (61 mg) for (XIV) and (2*R*,3*R*)-tartaric acid (78 mg) for (XV)] also dissolved in methanol (10 ml). These mixtures were then heated briefly at 323 K with magnetic stirring and then set aside to crystallize at room temperature. The resulting products were then collected by filtration and dried in air. Crystals suitable for single-crystal X-ray diffraction were grown by slow evaporation, at ambient temperature and in the presence of air, of solutions in acetone/aceto­nitrile (initial composition 1:1, *v*/*v*) for (I), methanol/aceto­nitrile (1:6, *v*/*v*) for (II), methanol/aceto­nitrile (1:1, *v*/*v*) for (III), ethyl acetate/acetone (2:1, *v*/*v*) for (IV) and (V), methanol/ethyl acetate (1:7, *v*/*v*) for (VI) and (VII), methanol for (VIII), (X), and (XIII)–(XV), methanol/ethyl acetate (3:2, *v*/*v*) for (IX), and methanol/ethyl acetate (1:1, *v*/*v*) for (XI) and (XII). M.p. (I) 374–378 K, (II) 390–394 K, (III) 422–428 K, (IV) 384–387 K, (V) 396–389 K, (VI) 396–399 K, (VII) 402–408 K, (VIII) 389–393 K, (IX) 441–445 K, (X) 408–412 K, (XI) 437–442 K, (XII) 430–435 K, (XIII) 390–396 K, (XIV) 435–437 K, (XV) 407–411 K.

## Refinement   

Crystal data, data collection and refinement details are summarized in Table 2 ▸. Two bad outlier reflections [(1,4,0) and (1,2,2)] were removed from the dataset for compound (V), and one bad outlier reflection (0,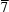,13) was removed from the dataset for compound (XV) before the final refinements. For compound (IV), calculation of the Flack *x* parameter (Flack, 1983 ▸) using 1089 quotients of the type [(*I* ^+^) − (*I* ^−^)]/[(*I* ^+^) + (*I* ^−^)] (Parsons *et al.*, 2013 ▸) gave a value 0.2 (3) in the absence of significant resonant scattering, the correct orientation of the structure of (IV) with respect to the polar axis directions remains uncertain. The correct absolute configurations for compounds (VI) and (VII) were established from the Flack *x* parameters: for (VI) *x* = 0.004 (5) calculated using 919 coefficients, and for (VII) *x* = 0.004 (10) calculated using 1045 coefficients. For the minor disorder component in compound (IV), the bonded distances and the 1,3-non-bonded distances were restrained to be the same as the corresponding distances in the major disorder components, subject to s.u. values of 0.01 and 0.02 Å, respectively, and the anisotropic displacement parameters for corresponding pairs of atoms in the two disorder components were constrained to be the same, giving occupancies of 0.907 (8) and 0.093 (8). Similar distance restraints were applied to the disordered carboxyl­ate group in compound (VII), where the displacement parameters for the disordered O atoms were subjected to similarity restraints, giving occupancies of 0.53 (9) and 0.47 (9). The disordered nitro group in compound (XII) was modelled over three sets of atomic sites, with similar restraints to those imposed in (VII) giving occupancies of 0.860 (5), 0.080 (4) and 0.069 (4). All H atoms, apart from those in the minor disorder component of compound (IV) and in the partial-occupancy water mol­ecule in compound (V), were located in difference maps. The H atoms bonded to C atoms, apart from those in the disordered anion of compound (XIV) which were permitted to ride at the locations found in difference maps, were then treated as riding atoms in geometrically idealized positions with C—H distances of 0.93 Å (alkenyl and aromatic), 0.96 Å (CH_3_), 0.97 Å (CH_2_) or 0.98 Å (aliphatic C—H), and with *U*_iso_(H) = *kU*_eq_(C), where *k* = 1.5 for the methyl groups, which were permitted to rotate but not to tilt, and 1.2 for all other H atoms bonded to C atoms: the H atoms in the minor disorder component of compound (IV) were included on exactly the same basis. For the H atoms bonded to N atoms, these were treated as riding atoms in the disordered structures (VII) and (XIV) with N—H distances of 0.89 Å and *U*_iso_H = 1.2*U*_eq_(N), but in all other compounds, the atomic coordinates of the H atoms bonded to N atoms were refined with *U*_iso_H = 1.2*U*_eq_(N), giving the N—H distances shown in Table 1 ▸. For the H atoms bonded to O atoms in compounds (XI), (XIII) and (XV), the atomic coordinates were refined with *U*_iso_(H) = 1.5*U*_eq_(O), giving the O—H distances shown in Table 1 ▸, but the partial occupancy H atoms bonded to O atoms in compound (XIV) were treated as riding atoms with O—H = 0.82 Å and *U*_iso_(H) = 1.5*U*_eq_(O).

## Supplementary Material

Crystal structure: contains datablock(s) global, I, II, III, IV, V, VI, VII, VIII, IX, X, XI, XII, XIII, XIV, XV. DOI: 10.1107/S2056989020014097/hb7950sup1.cif

Structure factors: contains datablock(s) I. DOI: 10.1107/S2056989020014097/hb7950Isup2.hkl

Structure factors: contains datablock(s) II. DOI: 10.1107/S2056989020014097/hb7950IIsup3.hkl

Structure factors: contains datablock(s) III. DOI: 10.1107/S2056989020014097/hb7950IIIsup4.hkl

Structure factors: contains datablock(s) IV. DOI: 10.1107/S2056989020014097/hb7950IVsup5.hkl

Structure factors: contains datablock(s) V. DOI: 10.1107/S2056989020014097/hb7950Vsup6.hkl

Structure factors: contains datablock(s) VI. DOI: 10.1107/S2056989020014097/hb7950VIsup7.hkl

Structure factors: contains datablock(s) VII. DOI: 10.1107/S2056989020014097/hb7950VIIsup8.hkl

Structure factors: contains datablock(s) VIII. DOI: 10.1107/S2056989020014097/hb7950VIIIsup9.hkl

Structure factors: contains datablock(s) IX. DOI: 10.1107/S2056989020014097/hb7950IXsup10.hkl

Structure factors: contains datablock(s) X. DOI: 10.1107/S2056989020014097/hb7950Xsup11.hkl

Structure factors: contains datablock(s) XI. DOI: 10.1107/S2056989020014097/hb7950XIsup12.hkl

Structure factors: contains datablock(s) XII. DOI: 10.1107/S2056989020014097/hb7950XIIsup13.hkl

Structure factors: contains datablock(s) XIII. DOI: 10.1107/S2056989020014097/hb7950XIIIsup14.hkl

Structure factors: contains datablock(s) XIV. DOI: 10.1107/S2056989020014097/hb7950XIVsup15.hkl

Structure factors: contains datablock(s) XV. DOI: 10.1107/S2056989020014097/hb7950XVsup16.hkl

Supporting information file. DOI: 10.1107/S2056989020014097/hb7950Isup17.cml

Supporting information file. DOI: 10.1107/S2056989020014097/hb7950IIsup18.cml

Supporting information file. DOI: 10.1107/S2056989020014097/hb7950IIIsup19.cml

Supporting information file. DOI: 10.1107/S2056989020014097/hb7950IVsup20.cml

Supporting information file. DOI: 10.1107/S2056989020014097/hb7950Vsup21.cml

Supporting information file. DOI: 10.1107/S2056989020014097/hb7950VIsup22.cml

Supporting information file. DOI: 10.1107/S2056989020014097/hb7950VIIsup23.cml

Supporting information file. DOI: 10.1107/S2056989020014097/hb7950VIIIsup24.cml

Supporting information file. DOI: 10.1107/S2056989020014097/hb7950IXsup25.cml

Supporting information file. DOI: 10.1107/S2056989020014097/hb7950Xsup26.cml

Supporting information file. DOI: 10.1107/S2056989020014097/hb7950XIsup27.cml

Supporting information file. DOI: 10.1107/S2056989020014097/hb7950XIIsup28.cml

Supporting information file. DOI: 10.1107/S2056989020014097/hb7950XIIIsup29.cml

Supporting information file. DOI: 10.1107/S2056989020014097/hb7950XIVsup30.cml

CCDC references: 2039887, 2039886, 2039885, 2039884, 2039883, 2039882, 2039881, 2039880, 2039879, 2039878, 2039877, 2039876, 2039875, 2039874, 2039873

Additional supporting information:  crystallographic information; 3D view; checkCIF report

## Figures and Tables

**Figure 2:**
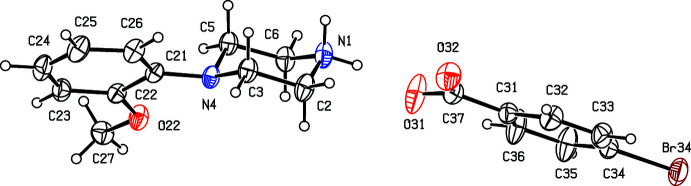
The independent components of compound (II) showing the atom-labelling scheme. Displacement ellipsoids are drawn at the 30% probability level.

**Figure 5:**
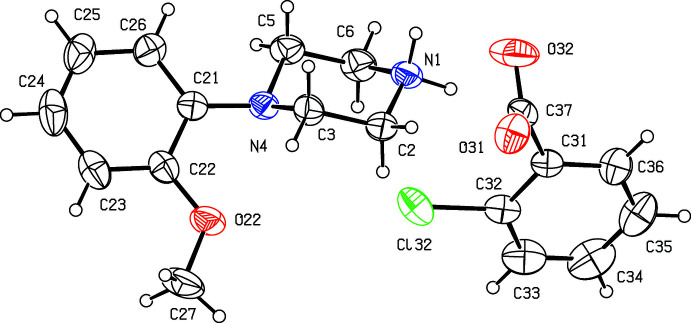
The independent components of compound (V) showing the atom-labelling scheme. Displacement ellipsoids are drawn at the 30% probability level.

**Figure 9:**
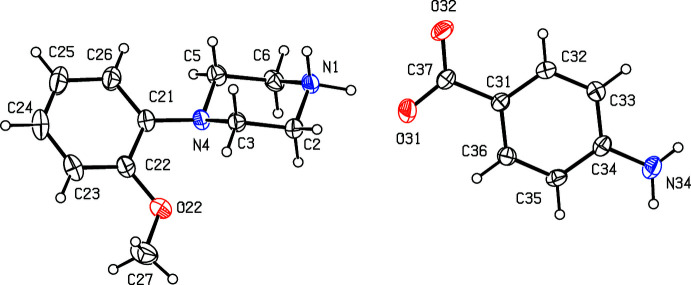
The independent components of compound (IX) showing the atom-labelling scheme. Displacement ellipsoids are drawn at the 30% probability level.

**Figure 10:**
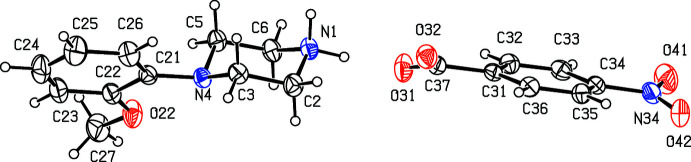
The independent components of compound (X) showing the atom-labelling scheme. Displacement ellipsoids are drawn at the 30% probability level.

**Table 1:** Hydrogen bonds and short inter-ion contacts (Å, °) *Cg*1, *Cg*2 and *Cg*3 represent the centroids of the rings (C31–C36), (C21–C26) and (C41–C46), respectively.

Compound	*D*—H⋯*A*	*D*—H	H⋯*A*	*D*⋯*A*	*D*—H⋯*A*
(I)	N1—H11⋯O31	1.02 (2)	1.60 (2)	2.616 (3)	176 (2)
	N1—H12⋯O32^i^	0.92 (3)	1.88 (3)	2.792 (3)	173 (2)
	C3—H3*A*⋯*Cg*1^i^	0.97	2.96	3.881 (3)	160
(II)	N1—H11⋯O31	0.89 (4)	1.75 (4)	2.620 (4)	168 (3)
	N1—H12⋯O32^i^	0.88 (4)	1.91 (4)	2.786 (4)	175 (4)
(III)	N1—H11⋯O31	0.88 (2)	1.83 (2)	2.684 (3)	163 (2)
	N1—H11⋯O32	0.88 (2)	2.60 (2)	3.060 (3)	113.6 (17)
	N1—H12⋯O32^i^	0.91 (3)	1.84 (3)	2.746 (3)	176 (3)
	C33—H33⋯O32^ii^	0.93	2.57	3.327 (3)	139
	C2—H2*B*⋯*Cg*2^iii^	0.97	2.77	3.482 (2)	131
(IV)	N1—H11⋯O31	0.99 (3)	1.72 (3)	2.694 (4)	167 (3)
	N1—H11⋯O32	0.99 (3)	2.51 (3)	3.131 (4)	120.9 (19)
	N1—H12⋯O32^iii^	0.88 (3)	1.83 (3)	2.679 (4)	161 (3)
	N1—H11⋯O41	0.99 (3)	1.77 (5)	2.67 (4)	151 (3)
	N1—H11⋯O42	0.99 (3)	2.52 (5)	3.20 (4)	126 (2)
	N1—H12⋯O42^iii^	0.88 (3)	1.83 (5)	2.63 (4)	151 (3)
	C34—H34⋯*Cg*2^iv^	0.93	2.74	3.543 (5)	145
	C44—H44⋯*Cg*2^iv^	0.93	2.99	3.73 (4)	137
	C26—H26⋯*Cg*3^v^	0.93	2.96	3.754 (17)	144
(V)	N1—H11⋯O31	0.97 (4)	1.74 (3)	2.682 (4)	162 (3)
	N1—H12⋯O32^i^	0.92 (4)	1.79 (4)	2.700 (5)	170 (4)
	C5—H5*B*⋯*Cg*1^ii^	0.97	2.87	3.554 (4)	128
	C34—H34⋯*Cg*2^vi^	0.93	2.93	3.658 (7)	136
(VI)	N1—H11⋯O31	0.75 (4)	1.98 (4)	2.726 (4)	170 (4)
	N1—H12⋯O32^vii^	0.88 (3)	1.86 (3)	2.712 (4)	163(3
	C25—H25⋯O32^viii^	0.93	2.56	3.488 (4)	173
	C26—H26⋯*Cg*1^viii^	0.93	2.93	3.697 (4)	141
(VII)	N1—H11⋯O31	0.89	1.80	2.66 (3)	162
	N1—H11⋯O33	0.89	1.93	2.80 (3)	165
	N1—H12⋯O31^ix^	0.89	1.97	2.83 (3)	162
	N1—H12⋯O33^ix^	0.89	1.74	2.60 (3)	161
	C25—H25⋯O34^*x*^	0.93	2.50	3.43 (3)	174
	C26—H26⋯*Cg*1^*x*^	0.93	2.93	3.716 (5)	143
(VIII)	N1—H11⋯O31	1.010 (15)	1.673 (15)	2.6696 (19)	168.6 (13)
	N1—H12⋯O32^i^	0.963 (16)	1.745 (16)	2.7077 (17)	178.2 (10)
(IX)	N1—H11⋯O31	1.068 (15)	1.547 (15)	2.6048 (15)	169.7 (14)
	N1—H12⋯O32^i^	0.942 (15)	1.861 (15)	2.7797 (15)	164.4 (14)
	N34—H34⋯O32^xi^	0.914 (16)	2.155 (16)	3.0535 (18)	167.5 (14)
(*X*)	N1—H11⋯O31	0.974 (16)	1.677 (16)	2.6500 (19)	176.8 (15)
	N1—H11⋯O32	0.974 (16)	2.581 (17)	3.2169 (17)	123.0 (12)
	N1—H12⋯O32^i^	0.948 (17)	1.837 (17)	2.7709 (18)	168.2 (16)
(XI)	N1—H11⋯O31	0.929 (16)	1.771 (16)	2.6837 (16)	166.8 (15)
	N1—H12⋯O41	0.911 (16)	1.939 (16)	2.8324 (19)	165.5 (14)
	O41—H41⋯O32^xii^	0.84 (2)	1.99 (2)	2.8156 (19)	168 (2)
	O41—H42⋯O51	0.90 (2)	1.91 (2)	2.810 (2)	172 (2)
	O51—H51⋯O31^i^	0.90 (2)	1.91 (2)	2.810 (2)	172 (2)
	O51—H52⋯O22^xii^	0.77 (2)	2.25 (2)	2.9544 (19)	153 (2)
	C25—H25⋯O36^i^	0.93	2.58	3.433 (2)	153
(XII)	N1—H11⋯O33	0.868 (18)	2.224 (18)	2.9120 (19)	136.1 (16)
	N1—H12⋯O31^i^	0.900 (18)	1.833 (18)	2.7142 (18)	165.9 (16)
	N1—H12⋯O32^i^	0.900 (19)	2.593 (17)	3.154 (2)	121.2 (13)
	C6—H6*A*⋯O34^xiii^	0.97	2.56	3.423 (2)	148
(XIII)	O33—H33⋯O32	1.07 (2)	1.37 (2)	2.4447 (16)	177.7 (16)
	O43—H43⋯O42	1.00 (2)	1.48 (2)	2.4707 (17)	174.0 (17)
	N11—H111⋯O32	0.927 (17)	1.891 (17)	2.8122 (18)	172.3 (16)
	N11—H112⋯O41^xiv^	0.930 (17)	1.848 (17)	2.7725 (17)	172.9 (13)
	N21—H211⋯O42	0.975 (15)	1.821 (15)	2.7926 (16)	174.5 (14)
	N21—H212⋯O31	0.895 (15)	2.283 (15)	2.9776 (17)	134.4 (12)
	N21—H212⋯O34^xv^	0.895 (15)	2.428 (15)	3.1170 (18)	134.1 (12)
	C16—H16*A*⋯O34^xv^	0.97	2.55	3.341 (2)	138
	C16—H16*B*⋯O44^xv^	0.97	2.52	3.338 (2)	141
	C25—H25*B*⋯*Cg*4^xvi^	0.97	2.92	3.8440 (16)	159
(XIV)	N1—H11⋯O31	0.89	2.01	2.894 (5)	171
	N1—H11⋯O33	0.89	1.73	2.584 (7)	160
	N1—H12⋯O41	0.89	1.97	2.8251 (15)	161
	O32—H32⋯O32^xvii^	0.82	1.54	2.355 (7)	176
	O34—H34⋯O34^xvii^	0.82	2.03	2.820 (9)	161
	O42—H42⋯O42^xviii^	0.82	1.62	2.4352 (12)	177
(XV)	N1—H11⋯O31	0.79 (4)	2.40 (4)	3.028 (4)	137 (3)
	N1—H11⋯O36^xii^	0.79 (4)	2.43 (4)	2.977 (4)	128 (3)
	N1—H11⋯O35^xix^	0.79 (4)	2.50 (3)	2.942 (3)	117 (3)
	N1—H12⋯O41	0.89 (4)	1.91 (4)	2.792 (5)	168 (3)
	O33—H33⋯O34^xx^	0.77 (4)	2.14 (4)	2.800 (3)	144 (4)
	O34—H34⋯O31^xx^	0.82 (4)	2.11 (4)	2.836 (3)	148 (3)
	O36—H36⋯O32^ii^	0.81 (4)	1.68 (4)	2.478 (3)	167(3
	O41—H41⋯O33^xxi^	0.82 (5)	1.94 (5)	2.753 (4)	167 (3)
	O41—H42⋯O31^xii^	0.87 (5)	1.90 (5)	2.766 (4)	169 (3)
	O51—H51⋯O41	0.98 (4)	1.80 (5)	2.776 (5)	172 (9)
	O51—H52⋯O22^xii^	0.97 (7)	2.22 (7)	3.054 (7)	144 (6)
	O51—H52⋯N4^xii^	0.97 (7)	2.48 (6)	3.307 (6)	143 (5)
	C23—H23⋯*Cg*2^xxii^	0.93	2.91	3.722 (4)	147

Symmetry codes: (i) 1 − *x*, 1 − *y*, 1 − *z*; (ii) 1 + *x*, *y*, *z*; (iii) *x*, 1 − *y*, −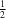 + *z*; (iv) −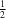 + *x*, 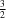 − *y*, −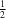 + *z*; (v) 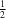 + *x*, −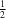 + *y*, *z*; (vi) −1 + *x*, *y*, 1 + *z*; (vii) 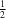 + *x*, 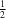 − *y*, 1 − *z*; (Viii) 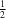 − *x*, 1 − *y*, −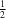 + *z*; (ix) −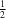 + *x*, 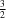 − *y*, 1 − *z*; (*x*) 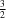 − *x*, 1 − *y*, 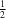 + *z*; (xi) *x*, 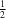 − *y*, −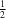 + *z*; (xii) −1 + *x*, *y*, *z*; (xiii) *x*, 1 + *y*, *z*; (xiv) *x*, −1 + *y*, *z*; (xv) −*x*, 1 − *y*, 1 − *z*; (xvi) 1 − *x*, −*y*, −*z*; (xvii) 1 − *x*, 1 − *y*, 2 − *z*; (xviii) 1 − *x*, −*y*, 2 − *z*; (xix) 2 − *x*, 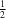 + *y*, 1 − *z*; (xx) 2 − *x*, −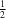 + *y*, 1 − *z*; (xxi) −1 + *x*, 1 + *y*, *z*; (xxii) 1 − *x*, 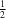 + *y*, −*z*.

**Table:** 

	(I)	(II)	(III)	(IV)	(V)
Crystal data					
Chemical formula	C_11_H_17_N_2_O^+^·C_7_H_4_ClO_2_^−^	C_11_H_17_N_2_O^+^·C_7_H_4_BrO_2_^−^	C_11_H_17_N_2_O^+^·C_7_H_4_IO_2_^−^	C_11_H_17_N_2_O^+^·C_7_H_4_FO_2_^−^	C_11_H_17_N_2_O^+^·C_7_H_4_ClO_2_^−^
*M*_r_	348.82	393.27	440.27	332.37	348.82
Crystal system, space group	Triclinic, *P* 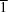	Triclinic, *P* 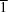	Triclinic, *P* 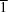	Monoclinic, *C* *c*	Monoclinic, *P*2_1_/*c*
Temperature (K)	296	296	296	296	296
*a*, *b*, *c* (Å)	7.401 (1), 7.888 (1), 15.410 (3)	7.4313 (5), 7.9163 (5), 15.5212 (9)	7.1129 (4), 11.2722 (7), 12.5923 (8)	19.940 (1), 10.2705 (7), 9.0148 (7)	7.9974 (8), 27.611 (2), 8.5972 (9)
α, β, γ (°)	100.28 (2), 94.40 (1), 94.14 (1)	101.565 (5), 94.780 (5), 92.691 (5)	69.852 (5), 74.681 (5), 79.121 (5)	90, 109.663 (8), 90	90, 106.40 (1), 90
*V* (Å^3^)	879.2 (2)	889.54 (10)	908.82 (10)	1738.5 (2)	1821.2 (3)
*Z*	2	2	2	4	4
Radiation type	Mo *K*α	Mo *K*α	Mo *K*α	Mo *K*α	Mo *K*α
μ (mm^−1^)	0.24	2.33	1.78	0.09	0.23
Crystal size (mm)	0.44 × 0.28 × 0.16	0.42 × 0.42 × 0.12	0.48 × 0.24 × 0.14	0.48 × 0.36 × 0.22	0.48 × 0.20 × 0.12

Data collection					
Diffractometer	Oxford Diffraction Xcalibur with Sapphire CCD	Oxford Diffraction Xcalibur with Sapphire CCD	Oxford Diffraction Xcalibur with Sapphire CCD	Oxford Diffraction Xcalibur with Sapphire CCD	Oxford Diffraction Xcalibur with Sapphire CCD
Absorption correction	Multi-scan (*CrysAlis RED*; Oxford Diffraction, 2009 ▸)	Multi-scan (*CrysAlis RED*; Oxford Diffraction, 2009 ▸)	Multi-scan (*CrysAlis RED*; Oxford Diffraction, 2009 ▸)	Multi-scan (*CrysAlis RED*; Oxford Diffraction, 2009 ▸)	Multi-scan (*CrysAlis RED*; Oxford Diffraction, 2009 ▸)
*T*_min_, *T*_max_	0.884, 0.963	0.258, 0.756	0.534, 0.779	0.884, 0.963	0.747, 0.973
No. of measured, independent and observed [*I* > 2σ(*I*)] reflections	6241, 3763, 2318	5996, 3739, 2989	6342, 3897, 3203	6204, 3343, 2786	13275, 3410, 2060
*R*_int_	0.019	0.018	0.012	0.012	0.030
(sin θ/λ)_max_ (Å^−1^)	0.650	0.652	0.660	0.658	0.607

Refinement					
*R*[*F* ^2^ > 2σ(*F* ^2^)], *wR*(*F* ^2^), *S*	0.053, 0.131, 1.01	0.043, 0.115, 1.05	0.026, 0.065, 1.02	0.036, 0.100, 1.03	0.067, 0.216, 1.03
No. of reflections	3763	3739	3897	3343	3410
No. of parameters	224	224	224	256	223
No. of restraints	0	0	0	25	0
H-atom treatment	H atoms treated by a mixture of independent and constrained refinement	H atoms treated by a mixture of independent and constrained refinement	H atoms treated by a mixture of independent and constrained refinement	H atoms treated by a mixture of independent and constrained refinement	H atoms treated by a mixture of independent and constrained refinement
Δρ_max_, Δρ_min_ (e Å^−3^)	0.19, −0.27	0.84, −0.55	0.52, −0.70	0.24, −0.14	1.15, −0.30
Absolute structure	–	–	–	Flack *x* determined using 1089 quotients [(*I* ^+^)−(*I* ^−^)]/[(*I* ^+^)+(*I* ^−^)] (Parsons *et al.*, 2013 ▸)	–
Absolute structure parameter	–	–	–	0.2 (3)	–

**Table:** 

	(VI)	(VII)	(VIII)	(IX)	(X)
Crystal data					
Chemical formula	C_11_H_17_N_2_O^+^·C_7_H_4_BrO_2_^−^	C_11_H_17_N_2_O^+^·C_7_H_4_IO_2_^−^	C_11_H_17_N_2_O^+^·C_8_H_7_O_2_^−^	C_11_H_17_N_2_O^+^·C_7_H_6_NO_2_^−^	C_11_H_17_N_2_O^+^·C_7_H_4_NO_4_^−^
*M*_r_	393.28	440.27	328.40	329.39	359.38
Crystal system, space group	Orthorhombic, *P*2_1_2_1_2_1_	Orthorhombic, *P*2_1_2_1_2_1_	Triclinic, *P* 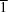	Monoclinic, *P*2_1_/*c*	Monoclinic, *P*2_1_/*c*
Temperature (K)	293	293	296	296	296
*a*, *b*, *c* (Å)	6.9824 (2), 13.2292 (4), 19.4903 (7)	7.0101 (4), 13.3796 (6), 19.5524 (6)	7.826 (1), 10.320 (2), 12.055 (3)	14.922 (1), 7.6951 (5), 15.560 (1)	7.5174 (5), 7.9761 (5), 29.860 (2)
α, β, γ (°)	90, 90, 90	90, 90, 90	78.37 (2), 78.27 (2), 73.83 (2)	90, 106.911 (8), 90	90, 97.322 (6), 90
*V* (Å^3^)	1800.35 (10)	1833.87 (14)	904.6 (3)	1709.4 (2)	1775.8 (2)
*Z*	4	4	2	4	4
Radiation type	Mo *K*α	Mo *K*α	Mo *K*α	Mo *K*α	Mo *K*α
μ (mm^−1^)	2.30	1.76	0.08	0.09	0.10
Crystal size (mm)	0.50 × 0.50 × 0.48	0.50 × 0.50 × 0.48	0.48 × 0.48 × 0.40	0.48 × 0.44 × 0.16	0.50 × 0.50 × 0.40

Data collection					
Diffractometer	Oxford Diffraction Xcalibur with Sapphire CCD	Oxford Diffraction Xcalibur with Sapphire CCD	Oxford Diffraction Xcalibur with Sapphire CCD	Oxford Diffraction Xcalibur with Sapphire CCD	Oxford Diffraction Xcalibur with Sapphire CCD
Absorption correction	Multi-scan (*CrysAlis RED*; Oxford Diffraction, 2009 ▸)	Multi-scan (*CrysAlis RED*; Oxford Diffraction, 2009 ▸)	Multi-scan (*CrysAlis RED*; Oxford Diffraction, 2009 ▸)	Multi-scan (*CrysAlis RED*; Oxford Diffraction, 2009 ▸)	Multi-scan (*CrysAlis RED*; Oxford Diffraction, 2009 ▸)
*T*_min_, *T*_max_	0.297, 0.331	0.373, 0.431	0.883, 0.968	0.830, 0.986	0.855, 0.961
No. of measured, independent and observed [*I* > 2σ(*I*)] reflections	13089, 3895, 2640	7500, 3735, 3036	6091, 3838, 2600	6720, 3668, 2606	13660, 3934, 2879
*R*_int_	0.033	0.019	0.013	0.014	0.019
(sin θ/λ)_max_ (Å^−1^)	0.654	0.655	0.653	0.651	0.658

Refinement					
*R*[*F* ^2^ > 2σ(*F* ^2^)], *wR*(*F* ^2^), *S*	0.035, 0.077, 0.94	0.032, 0.071, 1.05	0.042, 0.119, 1.06	0.039, 0.112, 1.10	0.040, 0.111, 1.03
No. of reflections	3895	3735	3838	3668	3934
No. of parameters	224	237	226	231	242
No. of restraints	0	17	0	0	0
H-atom treatment	H atoms treated by a mixture of independent and constrained refinement	H-atom parameters constrained	H atoms treated by a mixture of independent and constrained refinement	H atoms treated by a mixture of independent and constrained refinement	H atoms treated by a mixture of independent and constrained refinement
Δρ_max_, Δρ_min_ (e Å^−3^)	0.29, −0.53	0.46, −0.65	0.16, −0.16	0.15, −0.25	0.17, −0.15
Absolute structure	Flack *x* determined using 919 quotients [(*I* ^+^)−(*I* ^−^)]/[(*I* ^+^)+(*I* ^−^)] (Parsons *et al.*, 2013 ▸)	Flack *x* determined using 1045 quotients [(*I* ^+^)−(*I* ^−^)]/[(*I* ^+^)+(*I* ^−^)] (Parsons *et al.*, 2013 ▸)	–	–	–
Absolute structure parameter	0.004 (5)	0.004 (10)	–	–	–

**Table:** 

	(XI)	(XII)	(XIII)	(XIV)	(XV)
Crystal data					
Chemical formula	C_11_H_17_N_2_O^+^·C_7_H_3_N_2_O_6_^−^·2H_2_O	C_11_H_17_N_2_O^+^·C_6_H_2_N_3_O_7_^−^	C_11_H_17_N_2_O^+^·C_4_H_3_O_4_^−^	C_11_H_17_N_2_O^+^·C_4_H_3_O_4_^−^	C_11_H_17_N_2_O^+^·C_4_H_5_O_6_^−^·1.698H_2_O
*M*_r_	440.41	421.33	308.33	308.33	372.97
Crystal system, space group	Triclinic, *P* 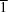	Triclinic, *P* 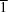	Triclinic, *P* 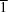	Triclinic, *P* 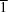	Monoclinic, *P*2_1_
Temperature (K)	296	296	296	296	296
*a*, *b*, *c* (Å)	7.8448 (6), 11.4635 (9), 12.0747 (9)	9.4151 (5), 9.8721 (5), 10.9572 (5)	11.1076 (6), 11.1164 (6), 13.7649 (7)	7.8546 (4), 8.9626 (6), 11.2056 (8)	7.479 (1), 7.065 (1), 17.788 (3)
α, β, γ (°)	94.406 (7), 105.075 (8), 93.717 (7)	77.524 (4), 81.360 (5), 81.002 (5)	80.353 (5), 78.353 (5), 74.406 (5)	79.043 (5), 87.715 (5), 85.840 (5)	90, 101.58 (2), 90
*V* (Å^3^)	1041.33 (14)	974.97 (9)	1591.76 (16)	772.15 (9)	920.8 (2)
*Z*	2	2	4	2	2
Radiation type	Mo *K*α	Mo *K*α	Mo *K*α	Mo *K*α	Mo *K*α
μ (mm^−1^)	0.11	0.12	0.10	0.10	0.11
Crystal size (mm)	0.48 × 0.48 × 0.44	0.48 × 0.48 × 0.24	0.48 × 0.40 × 0.36	0.48 × 0.48 × 0.34	0.36 × 0.32 × 0.12

Data collection					
Diffractometer	Oxford Diffraction Xcalibur with Sapphire CCD	Oxford Diffraction Xcalibur with Sapphire CCD	Oxford Diffraction Xcalibur with Sapphire CCD	Oxford Diffraction Xcalibur with Sapphire CCD	Oxford Diffraction Xcalibur with Sapphire CCD
Absorption correction	Multi-scan (*CrysAlis RED*; Oxford Diffraction, 2009 ▸)	Multi-scan (*CrysAlis RED*; Oxford Diffraction, 2009 ▸)	Multi-scan (*CrysAlis RED*; Oxford Diffraction, 2009 ▸)	Multi-scan (*CrysAlis RED*; Oxford Diffraction, 2009 ▸)	Multi-scan (*CrysAlis RED*; Oxford Diffraction, 2009 ▸)
*T*_min_, *T*_max_	0.892, 0.951	0.805, 0.973	0.863, 0.966	0.867, 0.967	0.956, 0.987
No. of measured, independent and observed [*I* > 2σ(*I*)] reflections	7353, 4419, 3409	12926, 4279, 3276	11727, 6817, 4221	5533, 3307, 2608	3655, 2895, 2062
*R*_int_	0.016	0.017	0.012	0.009	0.022
(sin θ/λ)_max_ (Å^−1^)	0.654	0.656	0.657	0.655	0.658

Refinement					
*R*[*F* ^2^ > 2σ(*F* ^2^)], *wR*(*F* ^2^), *S*	0.039, 0.108, 1.06	0.040, 0.119, 1.07	0.042, 0.121, 1.03	0.036, 0.105, 1.06	0.039, 0.081, 0.97
No. of reflections	4419	4279	6817	3307	2895
No. of parameters	300	317	415	240	263
No. of restraints	0	85	0	6	4
H-atom treatment	H atoms treated by a mixture of independent and constrained refinement	H atoms treated by a mixture of independent and constrained refinement	H atoms treated by a mixture of independent and constrained refinement	H-atom parameters constrained	H atoms treated by a mixture of independent and constrained refinement
Δρ_max_, Δρ_min_ (e Å^−3^)	0.23, −0.17	0.24, −0.27	0.15, −0.17	0.20, −0.15	0.14, −0.17

Computer programs: *CrysAlis CCD* and *CrysAlis RED* (Oxford Diffraction, 2009 ▸), *SHELXT* (Sheldrick, 2015*a* ▸), *SHELXL2014* (Sheldrick, 2015*b* ▸) and *PLATON* (Spek, 2020 ▸).

## supplementary crystallographic information

### 4-(2-Methoxyphenyl)piperazin-1-ium 4-chlorobenzoate (I) Crystal data

**Table:**

C_11_H_17_N_2_O^+^·C_7_H_4_ClO_2_^−^	*Z* = 2
*M**_r_* = 348.82	*F*(000) = 368
Triclinic, *P*1	*D*_x_ = 1.318 Mg m^−^^3^
*a* = 7.401 (1) Å	Mo *K*α radiation, λ = 0.71073 Å
*b* = 7.888 (1) Å	Cell parameters from 3770 reflections
*c* = 15.410 (3) Å	θ = 2.6–27.8°
α = 100.28 (2)°	µ = 0.24 mm^−^^1^
β = 94.40 (1)°	*T* = 296 K
γ = 94.14 (1)°	Plate, orange
*V* = 879.2 (2) Å^3^	0.44 × 0.28 × 0.16 mm

### 4-(2-Methoxyphenyl)piperazin-1-ium 4-chlorobenzoate (I) Data collection

**Table:**

Oxford Diffraction Xcalibur with Sapphire CCD diffractometer	3763 independent reflections
Radiation source: Enhance (Mo) X-ray Source	2318 reflections with *I* > 2σ(*I*)
Graphite monochromator	*R*_int_ = 0.019
ω scans	θ_max_ = 27.5°, θ_min_ = 2.6°
Absorption correction: multi-scan (CrysAlis RED; Oxford Diffraction, 2009)	*h* = −9→8
*T*_min_ = 0.884, *T*_max_ = 0.963	*k* = −10→6
6241 measured reflections	*l* = −19→19

### 4-(2-Methoxyphenyl)piperazin-1-ium 4-chlorobenzoate (I) Refinement

**Table:**

Refinement on *F*^2^	Primary atom site location: difference Fourier map
Least-squares matrix: full	Hydrogen site location: mixed
*R*[*F*^2^ > 2σ(*F*^2^)] = 0.053	H atoms treated by a mixture of independent and constrained refinement
*wR*(*F*^2^) = 0.131	*w* = 1/[σ^2^(*F*_o_^2^) + (0.0498*P*)^2^ + 0.3034*P*] where *P* = (*F*_o_^2^ + 2*F*_c_^2^)/3
*S* = 1.01	(Δ/σ)_max_ < 0.001
3763 reflections	Δρ_max_ = 0.19 e Å^−^^3^
224 parameters	Δρ_min_ = −0.27 e Å^−^^3^
0 restraints	

### 4-(2-Methoxyphenyl)piperazin-1-ium 4-chlorobenzoate (I) Special details

**Table:**

Experimental. Empirical absorption correction using spherical harmonics, implemented in SCALE3 ABSPACK scaling algorithm.
Geometry. All esds (except the esd in the dihedral angle between two l.s. planes) are estimated using the full covariance matrix. The cell esds are taken into account individually in the estimation of esds in distances, angles and torsion angles; correlations between esds in cell parameters are only used when they are defined by crystal symmetry. An approximate (isotropic) treatment of cell esds is used for estimating esds involving l.s. planes.

### 4-(2-Methoxyphenyl)piperazin-1-ium 4-chlorobenzoate (I) Fractional atomic coordinates and isotropic or equivalent isotropic displacement parameters (Å^2^)

**Table:**

	*x*	*y*	*z*	*U*_iso_*/*U*_eq_	
N1	0.3863 (3)	0.5719 (3)	0.61980 (13)	0.0537 (6)	
H11	0.349 (3)	0.654 (3)	0.5787 (16)	0.064*	
H12	0.494 (4)	0.526 (3)	0.6072 (17)	0.064*	
C2	0.2453 (4)	0.4243 (3)	0.61032 (16)	0.0595 (7)	
H2A	0.2389	0.3567	0.5509	0.071*	
H2B	0.1276	0.4677	0.6194	0.071*	
C3	0.2901 (4)	0.3104 (3)	0.67711 (14)	0.0492 (6)	
H3A	0.1964	0.2154	0.6709	0.059*	
H3B	0.4051	0.2623	0.6665	0.059*	
N4	0.3016 (3)	0.4131 (2)	0.76645 (11)	0.0417 (5)	
C5	0.4446 (3)	0.5568 (3)	0.77727 (15)	0.0456 (6)	
H5A	0.5614	0.5111	0.7685	0.055*	
H5B	0.4511	0.6234	0.8369	0.055*	
C6	0.4046 (4)	0.6728 (3)	0.71121 (15)	0.0489 (6)	
H6A	0.2928	0.7261	0.7232	0.059*	
H6B	0.5023	0.7641	0.7173	0.059*	
C21	0.3080 (3)	0.3202 (3)	0.83759 (14)	0.0402 (5)	
C22	0.2709 (3)	0.4034 (3)	0.92205 (14)	0.0401 (5)	
C23	0.2740 (3)	0.3164 (3)	0.99224 (16)	0.0500 (6)	
H23	0.2531	0.3740	1.0482	0.060*	
C24	0.3080 (4)	0.1439 (3)	0.97981 (17)	0.0565 (7)	
H24	0.3088	0.0854	1.0272	0.068*	
C25	0.3405 (4)	0.0595 (3)	0.89756 (18)	0.0588 (7)	
H25	0.3615	−0.0569	0.8889	0.071*	
C26	0.3420 (3)	0.1471 (3)	0.82735 (16)	0.0498 (6)	
H26	0.3663	0.0889	0.7721	0.060*	
O22	0.2258 (2)	0.5712 (2)	0.92836 (10)	0.0522 (4)	
C27	0.2117 (4)	0.6682 (3)	1.01422 (15)	0.0539 (6)	
H27A	0.1780	0.7816	1.0093	0.081*	
H27B	0.3267	0.6779	1.0488	0.081*	
H27C	0.1207	0.6107	1.0426	0.081*	
C31	0.2111 (3)	0.8543 (3)	0.37936 (15)	0.0450 (6)	
C32	0.1780 (4)	0.8004 (3)	0.28892 (17)	0.0597 (7)	
H32	0.1867	0.6850	0.2643	0.072*	
C33	0.1321 (4)	0.9151 (4)	0.23404 (17)	0.0622 (7)	
H33	0.1104	0.8772	0.1732	0.075*	
C34	0.1190 (3)	1.0848 (3)	0.27049 (16)	0.0498 (6)	
Cl34	0.06196 (11)	1.23102 (10)	0.20197 (5)	0.0724 (3)	
C35	0.1471 (5)	1.1410 (3)	0.35980 (18)	0.0712 (9)	
H35	0.1353	1.2559	0.3843	0.085*	
C36	0.1932 (5)	1.0247 (3)	0.41347 (17)	0.0697 (9)	
H36	0.2127	1.0631	0.4744	0.084*	
C37	0.2650 (4)	0.7306 (3)	0.43954 (18)	0.0529 (6)	
O31	0.2833 (4)	0.7908 (3)	0.52081 (13)	0.0955 (8)	
O32	0.2879 (3)	0.5797 (2)	0.40612 (13)	0.0704 (6)	

### 4-(2-Methoxyphenyl)piperazin-1-ium 4-chlorobenzoate (I) Atomic displacement parameters (Å^2^)

**Table:**

	*U*^11^	*U*^22^	*U*^33^	*U*^12^	*U*^13^	*U*^23^
N1	0.0757 (16)	0.0508 (13)	0.0397 (12)	0.0186 (11)	0.0112 (11)	0.0142 (10)
C2	0.079 (2)	0.0591 (16)	0.0380 (14)	0.0075 (14)	−0.0055 (13)	0.0050 (12)
C3	0.0626 (16)	0.0437 (13)	0.0383 (13)	0.0038 (12)	−0.0001 (11)	0.0014 (10)
N4	0.0525 (12)	0.0389 (10)	0.0326 (10)	0.0038 (9)	0.0011 (9)	0.0048 (8)
C5	0.0538 (15)	0.0426 (13)	0.0393 (13)	0.0030 (11)	0.0035 (11)	0.0057 (10)
C6	0.0641 (17)	0.0412 (13)	0.0419 (13)	0.0079 (12)	0.0088 (12)	0.0063 (10)
C21	0.0399 (13)	0.0386 (12)	0.0416 (13)	0.0046 (10)	0.0003 (10)	0.0072 (10)
C22	0.0420 (13)	0.0385 (12)	0.0402 (13)	0.0065 (10)	0.0027 (10)	0.0075 (10)
C23	0.0566 (16)	0.0540 (15)	0.0411 (14)	0.0043 (12)	0.0040 (11)	0.0131 (11)
C24	0.0690 (18)	0.0498 (15)	0.0544 (16)	0.0031 (13)	−0.0027 (13)	0.0240 (13)
C25	0.0725 (19)	0.0388 (13)	0.0652 (18)	0.0073 (12)	−0.0056 (14)	0.0143 (13)
C26	0.0602 (16)	0.0420 (13)	0.0458 (14)	0.0077 (11)	0.0015 (12)	0.0046 (11)
O22	0.0747 (12)	0.0445 (9)	0.0404 (9)	0.0210 (8)	0.0131 (8)	0.0070 (7)
C27	0.0599 (17)	0.0488 (14)	0.0502 (15)	0.0053 (12)	0.0115 (13)	−0.0012 (12)
C31	0.0465 (14)	0.0442 (13)	0.0452 (14)	0.0083 (11)	0.0072 (11)	0.0075 (11)
C32	0.0740 (19)	0.0551 (16)	0.0502 (16)	0.0264 (14)	0.0077 (14)	0.0010 (12)
C33	0.0737 (19)	0.0767 (19)	0.0376 (14)	0.0250 (15)	0.0068 (13)	0.0065 (13)
C34	0.0507 (15)	0.0572 (15)	0.0455 (14)	0.0055 (12)	0.0047 (12)	0.0197 (12)
Cl34	0.0783 (5)	0.0800 (5)	0.0671 (5)	0.0054 (4)	−0.0011 (4)	0.0394 (4)
C35	0.119 (3)	0.0433 (15)	0.0511 (17)	0.0156 (16)	−0.0029 (16)	0.0085 (12)
C36	0.120 (3)	0.0498 (15)	0.0375 (14)	0.0171 (16)	−0.0043 (15)	0.0048 (12)
C37	0.0586 (16)	0.0494 (15)	0.0546 (16)	0.0122 (12)	0.0095 (13)	0.0153 (12)
O31	0.175 (2)	0.0646 (13)	0.0529 (13)	0.0405 (14)	−0.0007 (14)	0.0189 (10)
O32	0.0899 (15)	0.0484 (11)	0.0781 (13)	0.0266 (10)	0.0171 (11)	0.0138 (10)

### 4-(2-Methoxyphenyl)piperazin-1-ium 4-chlorobenzoate (I) Geometric parameters (Å, º)

**Table:**

N1—C6	1.481 (3)	C24—C25	1.370 (4)
N1—C2	1.485 (3)	C24—H24	0.9300
N1—H11	1.02 (3)	C25—C26	1.385 (3)
N1—H12	0.92 (3)	C25—H25	0.9300
C2—C3	1.516 (3)	C26—H26	0.9300
C2—H2A	0.9700	O22—C27	1.421 (3)
C2—H2B	0.9700	C27—H27A	0.9600
C3—N4	1.461 (3)	C27—H27B	0.9600
C3—H3A	0.9700	C27—H27C	0.9600
C3—H3B	0.9700	C31—C36	1.373 (3)
N4—C21	1.423 (3)	C31—C32	1.380 (3)
N4—C5	1.471 (3)	C31—C37	1.515 (3)
C5—C6	1.512 (3)	C32—C33	1.386 (3)
C5—H5A	0.9700	C32—H32	0.9300
C5—H5B	0.9700	C33—C34	1.369 (4)
C6—H6A	0.9700	C33—H33	0.9300
C6—H6B	0.9700	C34—C35	1.364 (3)
C21—C26	1.389 (3)	C34—Cl34	1.750 (2)
C21—C22	1.406 (3)	C35—C36	1.384 (3)
C22—O22	1.377 (2)	C35—H35	0.9300
C22—C23	1.380 (3)	C36—H36	0.9300
C23—C24	1.384 (3)	C37—O32	1.239 (3)
C23—H23	0.9300	C37—O31	1.251 (3)
			
C6—N1—C2	110.65 (19)	C22—C23—H23	119.8
C6—N1—H11	107.0 (14)	C24—C23—H23	119.8
C2—N1—H11	109.8 (15)	C25—C24—C23	119.8 (2)
C6—N1—H12	109.8 (17)	C25—C24—H24	120.1
C2—N1—H12	106.9 (16)	C23—C24—H24	120.1
H11—N1—H12	113 (2)	C24—C25—C26	120.1 (2)
N1—C2—C3	110.5 (2)	C24—C25—H25	120.0
N1—C2—H2A	109.6	C26—C25—H25	120.0
C3—C2—H2A	109.6	C25—C26—C21	121.5 (2)
N1—C2—H2B	109.6	C25—C26—H26	119.3
C3—C2—H2B	109.6	C21—C26—H26	119.3
H2A—C2—H2B	108.1	C22—O22—C27	117.86 (17)
N4—C3—C2	109.35 (19)	O22—C27—H27A	109.5
N4—C3—H3A	109.8	O22—C27—H27B	109.5
C2—C3—H3A	109.8	H27A—C27—H27B	109.5
N4—C3—H3B	109.8	O22—C27—H27C	109.5
C2—C3—H3B	109.8	H27A—C27—H27C	109.5
H3A—C3—H3B	108.3	H27B—C27—H27C	109.5
C21—N4—C3	116.59 (17)	C36—C31—C32	117.8 (2)
C21—N4—C5	113.53 (18)	C36—C31—C37	120.7 (2)
C3—N4—C5	110.53 (18)	C32—C31—C37	121.5 (2)
N4—C5—C6	110.35 (19)	C31—C32—C33	121.2 (2)
N4—C5—H5A	109.6	C31—C32—H32	119.4
C6—C5—H5A	109.6	C33—C32—H32	119.4
N4—C5—H5B	109.6	C34—C33—C32	119.2 (2)
C6—C5—H5B	109.6	C34—C33—H33	120.4
H5A—C5—H5B	108.1	C32—C33—H33	120.4
N1—C6—C5	110.38 (19)	C35—C34—C33	120.9 (2)
N1—C6—H6A	109.6	C35—C34—Cl34	119.4 (2)
C5—C6—H6A	109.6	C33—C34—Cl34	119.7 (2)
N1—C6—H6B	109.6	C34—C35—C36	119.0 (2)
C5—C6—H6B	109.6	C34—C35—H35	120.5
H6A—C6—H6B	108.1	C36—C35—H35	120.5
C26—C21—C22	117.5 (2)	C31—C36—C35	121.9 (2)
C26—C21—N4	123.2 (2)	C31—C36—H36	119.1
C22—C21—N4	119.20 (19)	C35—C36—H36	119.1
O22—C22—C23	123.4 (2)	O32—C37—O31	124.7 (2)
O22—C22—C21	115.91 (18)	O32—C37—C31	119.0 (2)
C23—C22—C21	120.7 (2)	O31—C37—C31	116.3 (2)
C22—C23—C24	120.4 (2)		
			
C6—N1—C2—C3	−56.8 (3)	C24—C25—C26—C21	−1.1 (4)
N1—C2—C3—N4	58.6 (3)	C22—C21—C26—C25	−0.3 (4)
C2—C3—N4—C21	168.3 (2)	N4—C21—C26—C25	−177.6 (2)
C2—C3—N4—C5	−60.1 (3)	C23—C22—O22—C27	10.9 (3)
C21—N4—C5—C6	−167.25 (18)	C21—C22—O22—C27	−171.5 (2)
C3—N4—C5—C6	59.6 (2)	C36—C31—C32—C33	1.3 (4)
C2—N1—C6—C5	55.6 (3)	C37—C31—C32—C33	−178.7 (2)
N4—C5—C6—N1	−56.8 (3)	C31—C32—C33—C34	−0.2 (4)
C3—N4—C21—C26	13.9 (3)	C32—C33—C34—C35	−1.3 (4)
C5—N4—C21—C26	−116.3 (2)	C32—C33—C34—Cl34	179.8 (2)
C3—N4—C21—C22	−163.3 (2)	C33—C34—C35—C36	1.4 (5)
C5—N4—C21—C22	66.5 (3)	Cl34—C34—C35—C36	−179.6 (2)
C26—C21—C22—O22	−175.8 (2)	C32—C31—C36—C35	−1.2 (5)
N4—C21—C22—O22	1.6 (3)	C37—C31—C36—C35	178.9 (3)
C26—C21—C22—C23	1.9 (3)	C34—C35—C36—C31	−0.2 (5)
N4—C21—C22—C23	179.3 (2)	C36—C31—C37—O32	−176.7 (3)
O22—C22—C23—C24	175.4 (2)	C32—C31—C37—O32	3.3 (4)
C21—C22—C23—C24	−2.1 (4)	C36—C31—C37—O31	3.2 (4)
C22—C23—C24—C25	0.6 (4)	C32—C31—C37—O31	−176.7 (3)
C23—C24—C25—C26	1.0 (4)		

### 4-(2-Methoxyphenyl)piperazin-1-ium 4-chlorobenzoate (I) Hydrogen-bond geometry (Å, º)

**Table:**

*D*—H···*A*	*D*—H	H···*A*	*D*···*A*	*D*—H···*A*
N1—H11···O31	1.02 (2)	1.60 (2)	2.616 (3)	176 (2)
N1—H12···O32^i^	0.92 (3)	1.88 (3)	2.792 (3)	173 (2)
C3—H3*A*···*Cg*1^i^	0.97	2.96	3.881 (3)	160

Symmetry code: (i) −*x*+1, −*y*+1, −*z*+1.

### 4-(2-Methoxyphenyl)piperazin-1-ium 4-bromobenzoate (II) Crystal data

**Table:**

C_11_H_17_N_2_O^+^·C_7_H_4_BrO_2_^−^	*Z* = 2
*M**_r_* = 393.27	*F*(000) = 404
Triclinic, *P*1	*D*_x_ = 1.468 Mg m^−^^3^
*a* = 7.4313 (5) Å	Mo *K*α radiation, λ = 0.71073 Å
*b* = 7.9163 (5) Å	Cell parameters from 3779 reflections
*c* = 15.5212 (9) Å	θ = 2.6–27.6°
α = 101.565 (5)°	µ = 2.33 mm^−^^1^
β = 94.780 (5)°	*T* = 296 K
γ = 92.691 (5)°	Plate, yellow
*V* = 889.54 (10) Å^3^	0.42 × 0.42 × 0.12 mm

### 4-(2-Methoxyphenyl)piperazin-1-ium 4-bromobenzoate (II) Data collection

**Table:**

Oxford Diffraction Xcalibur with Sapphire CCD diffractometer	3739 independent reflections
Radiation source: Enhance (Mo) X-ray Source	2989 reflections with *I* > 2σ(*I*)
Graphite monochromator	*R*_int_ = 0.018
ω scans	θ_max_ = 27.6°, θ_min_ = 2.6°
Absorption correction: multi-scan (CrysAlis RED; Oxford Diffraction, 2009)	*h* = −9→8
*T*_min_ = 0.258, *T*_max_ = 0.756	*k* = −10→10
5996 measured reflections	*l* = −19→16

### 4-(2-Methoxyphenyl)piperazin-1-ium 4-bromobenzoate (II) Refinement

**Table:**

Refinement on *F*^2^	Primary atom site location: difference Fourier map
Least-squares matrix: full	Hydrogen site location: mixed
*R*[*F*^2^ > 2σ(*F*^2^)] = 0.043	H atoms treated by a mixture of independent and constrained refinement
*wR*(*F*^2^) = 0.115	*w* = 1/[σ^2^(*F*_o_^2^) + (0.0576*P*)^2^ + 0.570*P*] where *P* = (*F*_o_^2^ + 2*F*_c_^2^)/3
*S* = 1.05	(Δ/σ)_max_ < 0.001
3739 reflections	Δρ_max_ = 0.84 e Å^−^^3^
224 parameters	Δρ_min_ = −0.55 e Å^−^^3^
0 restraints	

### 4-(2-Methoxyphenyl)piperazin-1-ium 4-bromobenzoate (II) Special details

**Table:**

Experimental. Empirical absorption correction using spherical harmonics, implemented in SCALE3 ABSPACK scaling algorithm.
Geometry. All esds (except the esd in the dihedral angle between two l.s. planes) are estimated using the full covariance matrix. The cell esds are taken into account individually in the estimation of esds in distances, angles and torsion angles; correlations between esds in cell parameters are only used when they are defined by crystal symmetry. An approximate (isotropic) treatment of cell esds is used for estimating esds involving l.s. planes.

### 4-(2-Methoxyphenyl)piperazin-1-ium 4-bromobenzoate (II) Fractional atomic coordinates and isotropic or equivalent isotropic displacement parameters (Å^2^)

**Table:**

	*x*	*y*	*z*	*U*_iso_*/*U*_eq_	
N1	0.3878 (4)	0.5725 (3)	0.62065 (16)	0.0514 (6)	
H11	0.361 (5)	0.638 (5)	0.582 (2)	0.062*	
H12	0.494 (5)	0.532 (5)	0.610 (2)	0.062*	
C2	0.2490 (5)	0.4279 (4)	0.61017 (19)	0.0557 (8)	
H2A	0.2421	0.3600	0.5504	0.067*	
H2B	0.1318	0.4735	0.6198	0.067*	
C3	0.2949 (4)	0.3139 (4)	0.67528 (18)	0.0471 (7)	
H3A	0.2023	0.2204	0.6683	0.057*	
H3B	0.4096	0.2641	0.6642	0.057*	
N4	0.3062 (3)	0.4172 (3)	0.76485 (13)	0.0385 (5)	
C5	0.4474 (4)	0.5584 (3)	0.77722 (17)	0.0424 (6)	
H5A	0.5640	0.5108	0.7685	0.051*	
H5B	0.4531	0.6256	0.8371	0.051*	
C6	0.4075 (4)	0.6739 (4)	0.71261 (18)	0.0470 (7)	
H6A	0.2968	0.7303	0.7252	0.056*	
H6B	0.5051	0.7627	0.7193	0.056*	
C21	0.3137 (3)	0.3252 (3)	0.83448 (17)	0.0375 (5)	
C22	0.2745 (3)	0.4102 (3)	0.91928 (17)	0.0387 (6)	
C23	0.2796 (4)	0.3234 (4)	0.98826 (19)	0.0470 (6)	
H23	0.2571	0.3812	1.0443	0.056*	
C24	0.3181 (4)	0.1505 (4)	0.9745 (2)	0.0549 (8)	
H24	0.3210	0.0927	1.0212	0.066*	
C25	0.3515 (5)	0.0658 (4)	0.8927 (2)	0.0556 (8)	
H25	0.3747	−0.0506	0.8832	0.067*	
C26	0.3511 (4)	0.1526 (4)	0.8233 (2)	0.0473 (7)	
H26	0.3765	0.0935	0.7680	0.057*	
O22	0.2276 (3)	0.5771 (2)	0.92653 (12)	0.0505 (5)	
C27	0.2084 (4)	0.6742 (4)	1.01265 (19)	0.0504 (7)	
H27A	0.1755	0.7884	1.0087	0.076*	
H27B	0.3209	0.6814	1.0488	0.076*	
H27C	0.1156	0.6182	1.0386	0.076*	
C31	0.2104 (4)	0.8486 (4)	0.38246 (18)	0.0423 (6)	
C32	0.1691 (5)	0.7880 (4)	0.2931 (2)	0.0552 (8)	
H32	0.1715	0.6704	0.2697	0.066*	
C33	0.1240 (5)	0.8992 (4)	0.2376 (2)	0.0574 (8)	
H33	0.0956	0.8571	0.1774	0.069*	
C34	0.1217 (4)	1.0726 (4)	0.27254 (19)	0.0457 (6)	
Br34	0.06068 (5)	1.22764 (5)	0.19700 (2)	0.06693 (16)	
C35	0.1596 (6)	1.1360 (4)	0.3608 (2)	0.0713 (11)	
H35	0.1558	1.2533	0.3841	0.086*	
C36	0.2042 (6)	1.0211 (4)	0.4153 (2)	0.0703 (11)	
H36	0.2305	1.0633	0.4756	0.084*	
C37	0.2644 (4)	0.7278 (4)	0.4432 (2)	0.0506 (7)	
O31	0.2859 (5)	0.7932 (3)	0.52394 (16)	0.0910 (10)	
O32	0.2844 (4)	0.5748 (3)	0.41048 (16)	0.0653 (6)	

### 4-(2-Methoxyphenyl)piperazin-1-ium 4-bromobenzoate (II) Atomic displacement parameters (Å^2^)

**Table:**

	*U*^11^	*U*^22^	*U*^33^	*U*^12^	*U*^13^	*U*^23^
N1	0.0781 (19)	0.0471 (14)	0.0325 (12)	0.0163 (13)	0.0077 (12)	0.0123 (10)
C2	0.080 (2)	0.0522 (17)	0.0310 (14)	0.0068 (15)	−0.0080 (14)	0.0033 (12)
C3	0.0635 (18)	0.0421 (15)	0.0322 (13)	0.0027 (13)	−0.0021 (12)	0.0020 (11)
N4	0.0514 (13)	0.0347 (11)	0.0269 (10)	0.0017 (9)	−0.0025 (9)	0.0031 (8)
C5	0.0555 (16)	0.0378 (14)	0.0320 (13)	0.0012 (12)	0.0000 (11)	0.0043 (11)
C6	0.0682 (19)	0.0364 (14)	0.0366 (14)	0.0074 (13)	0.0075 (13)	0.0059 (11)
C21	0.0396 (13)	0.0370 (13)	0.0347 (13)	0.0021 (10)	−0.0032 (10)	0.0077 (10)
C22	0.0404 (13)	0.0393 (14)	0.0361 (13)	0.0049 (11)	0.0001 (11)	0.0080 (11)
C23	0.0519 (16)	0.0534 (16)	0.0365 (14)	0.0014 (13)	−0.0001 (12)	0.0134 (12)
C24	0.0651 (19)	0.0513 (17)	0.0516 (18)	−0.0022 (14)	−0.0066 (14)	0.0251 (14)
C25	0.072 (2)	0.0348 (14)	0.059 (2)	0.0056 (13)	−0.0080 (15)	0.0128 (13)
C26	0.0577 (17)	0.0378 (14)	0.0437 (15)	0.0041 (12)	−0.0048 (13)	0.0056 (12)
O22	0.0738 (14)	0.0445 (11)	0.0337 (10)	0.0191 (10)	0.0067 (9)	0.0052 (8)
C27	0.0581 (18)	0.0478 (16)	0.0412 (15)	0.0055 (13)	0.0079 (13)	−0.0025 (12)
C31	0.0473 (15)	0.0421 (14)	0.0384 (14)	0.0069 (11)	0.0050 (11)	0.0090 (11)
C32	0.070 (2)	0.0458 (16)	0.0469 (17)	0.0186 (14)	0.0023 (15)	0.0005 (13)
C33	0.070 (2)	0.067 (2)	0.0331 (15)	0.0162 (16)	0.0044 (14)	0.0033 (14)
C34	0.0459 (15)	0.0520 (16)	0.0416 (15)	0.0018 (12)	−0.0004 (12)	0.0175 (13)
Br34	0.0726 (2)	0.0759 (3)	0.0590 (2)	−0.00290 (17)	−0.00806 (16)	0.03751 (18)
C35	0.125 (3)	0.0384 (16)	0.0471 (18)	0.0068 (18)	−0.0112 (19)	0.0089 (14)
C36	0.129 (3)	0.0435 (17)	0.0343 (16)	0.0120 (19)	−0.0120 (18)	0.0038 (13)
C37	0.0537 (17)	0.0488 (17)	0.0534 (18)	0.0084 (13)	0.0048 (14)	0.0190 (14)
O31	0.171 (3)	0.0586 (15)	0.0454 (14)	0.0315 (17)	−0.0072 (16)	0.0173 (11)
O32	0.0879 (17)	0.0400 (12)	0.0704 (15)	0.0200 (11)	0.0089 (13)	0.0127 (10)

### 4-(2-Methoxyphenyl)piperazin-1-ium 4-bromobenzoate (II) Geometric parameters (Å, º)

**Table:**

N1—C2	1.479 (4)	C24—C25	1.361 (5)
N1—C6	1.483 (4)	C24—H24	0.9300
N1—H11	0.89 (4)	C25—C26	1.388 (4)
N1—H12	0.88 (4)	C25—H25	0.9300
C2—C3	1.513 (4)	C26—H26	0.9300
C2—H2A	0.9700	O22—C27	1.424 (3)
C2—H2B	0.9700	C27—H27A	0.9600
C3—N4	1.458 (3)	C27—H27B	0.9600
C3—H3A	0.9700	C27—H27C	0.9600
C3—H3B	0.9700	C31—C36	1.363 (4)
N4—C21	1.418 (3)	C31—C32	1.377 (4)
N4—C5	1.470 (4)	C31—C37	1.515 (4)
C5—C6	1.508 (4)	C32—C33	1.384 (4)
C5—H5A	0.9700	C32—H32	0.9300
C5—H5B	0.9700	C33—C34	1.372 (4)
C6—H6A	0.9700	C33—H33	0.9300
C6—H6B	0.9700	C34—C35	1.361 (4)
C21—C26	1.387 (4)	C34—Br34	1.905 (3)
C21—C22	1.413 (4)	C35—C36	1.394 (4)
C22—O22	1.367 (3)	C35—H35	0.9300
C22—C23	1.382 (4)	C36—H36	0.9300
C23—C24	1.390 (4)	C37—O32	1.237 (4)
C23—H23	0.9300	C37—O31	1.251 (4)
			
C2—N1—C6	110.8 (2)	C22—C23—H23	119.8
C2—N1—H11	110 (2)	C24—C23—H23	119.8
C6—N1—H11	112 (2)	C25—C24—C23	119.9 (3)
C2—N1—H12	110 (2)	C25—C24—H24	120.1
C6—N1—H12	107 (2)	C23—C24—H24	120.1
H11—N1—H12	106 (3)	C24—C25—C26	120.2 (3)
N1—C2—C3	110.6 (2)	C24—C25—H25	119.9
N1—C2—H2A	109.5	C26—C25—H25	119.9
C3—C2—H2A	109.5	C21—C26—C25	121.6 (3)
N1—C2—H2B	109.5	C21—C26—H26	119.2
C3—C2—H2B	109.5	C25—C26—H26	119.2
H2A—C2—H2B	108.1	C22—O22—C27	117.7 (2)
N4—C3—C2	109.2 (2)	O22—C27—H27A	109.5
N4—C3—H3A	109.8	O22—C27—H27B	109.5
C2—C3—H3A	109.8	H27A—C27—H27B	109.5
N4—C3—H3B	109.8	O22—C27—H27C	109.5
C2—C3—H3B	109.8	H27A—C27—H27C	109.5
H3A—C3—H3B	108.3	H27B—C27—H27C	109.5
C21—N4—C3	116.6 (2)	C36—C31—C32	118.4 (3)
C21—N4—C5	113.3 (2)	C36—C31—C37	120.4 (3)
C3—N4—C5	110.7 (2)	C32—C31—C37	121.2 (3)
N4—C5—C6	110.4 (2)	C31—C32—C33	121.0 (3)
N4—C5—H5A	109.6	C31—C32—H32	119.5
C6—C5—H5A	109.6	C33—C32—H32	119.5
N4—C5—H5B	109.6	C34—C33—C32	119.1 (3)
C6—C5—H5B	109.6	C34—C33—H33	120.4
H5A—C5—H5B	108.1	C32—C33—H33	120.4
N1—C6—C5	110.7 (2)	C35—C34—C33	121.2 (3)
N1—C6—H6A	109.5	C35—C34—Br34	119.1 (2)
C5—C6—H6A	109.5	C33—C34—Br34	119.7 (2)
N1—C6—H6B	109.5	C34—C35—C36	118.5 (3)
C5—C6—H6B	109.5	C34—C35—H35	120.7
H6A—C6—H6B	108.1	C36—C35—H35	120.7
C26—C21—C22	117.5 (2)	C31—C36—C35	121.7 (3)
C26—C21—N4	123.4 (2)	C31—C36—H36	119.1
C22—C21—N4	119.0 (2)	C35—C36—H36	119.1
O22—C22—C23	123.8 (2)	O32—C37—O31	124.9 (3)
O22—C22—C21	115.9 (2)	O32—C37—C31	118.9 (3)
C23—C22—C21	120.3 (2)	O31—C37—C31	116.2 (3)
C22—C23—C24	120.5 (3)		
			
C6—N1—C2—C3	−56.5 (3)	C22—C21—C26—C25	−0.5 (4)
N1—C2—C3—N4	58.8 (3)	N4—C21—C26—C25	−177.9 (3)
C2—C3—N4—C21	168.3 (2)	C24—C25—C26—C21	−1.2 (5)
C2—C3—N4—C5	−60.2 (3)	C23—C22—O22—C27	9.3 (4)
C21—N4—C5—C6	−167.6 (2)	C21—C22—O22—C27	−172.6 (2)
C3—N4—C5—C6	59.3 (3)	C36—C31—C32—C33	0.6 (5)
C2—N1—C6—C5	55.0 (3)	C37—C31—C32—C33	−178.5 (3)
N4—C5—C6—N1	−56.0 (3)	C31—C32—C33—C34	0.4 (5)
C3—N4—C21—C26	14.8 (4)	C32—C33—C34—C35	−1.3 (5)
C5—N4—C21—C26	−115.4 (3)	C32—C33—C34—Br34	179.8 (2)
C3—N4—C21—C22	−162.6 (2)	C33—C34—C35—C36	1.1 (6)
C5—N4—C21—C22	67.1 (3)	Br34—C34—C35—C36	−179.9 (3)
C26—C21—C22—O22	−176.2 (2)	C32—C31—C36—C35	−0.7 (6)
N4—C21—C22—O22	1.4 (4)	C37—C31—C36—C35	178.3 (4)
C26—C21—C22—C23	2.0 (4)	C34—C35—C36—C31	−0.1 (7)
N4—C21—C22—C23	179.6 (2)	C36—C31—C37—O32	−173.7 (3)
O22—C22—C23—C24	176.1 (3)	C32—C31—C37—O32	5.4 (5)
C21—C22—C23—C24	−1.9 (4)	C36—C31—C37—O31	6.2 (5)
C22—C23—C24—C25	0.2 (5)	C32—C31—C37—O31	−174.8 (3)
C23—C24—C25—C26	1.4 (5)		

### 4-(2-Methoxyphenyl)piperazin-1-ium 4-bromobenzoate (II) Hydrogen-bond geometry (Å, º)

**Table:**

*D*—H···*A*	*D*—H	H···*A*	*D*···*A*	*D*—H···*A*
N1—H11···O31	0.89 (4)	1.75 (4)	2.620 (4)	168 (3)
N1—H12···O32^i^	0.88 (4)	1.91 (4)	2.786 (4)	175 (4)

Symmetry code: (i) −*x*+1, −*y*+1, −*z*+1.

### 4-(2-Methoxyphenyl)piperazin-1-ium 4-iodobenzoate (III) Crystal data

**Table:**

C_11_H_17_N_2_O^+^·C_7_H_4_IO_2_^−^	*Z* = 2
*M**_r_* = 440.27	*F*(000) = 440
Triclinic, *P*1	*D*_x_ = 1.609 Mg m^−^^3^
*a* = 7.1129 (4) Å	Mo *K*α radiation, λ = 0.71073 Å
*b* = 11.2722 (7) Å	Cell parameters from 3897 reflections
*c* = 12.5923 (8) Å	θ = 3.0–28.0°
α = 69.852 (5)°	µ = 1.78 mm^−^^1^
β = 74.681 (5)°	*T* = 296 K
γ = 79.121 (5)°	Needle, orange
*V* = 908.82 (10) Å^3^	0.48 × 0.24 × 0.14 mm

### 4-(2-Methoxyphenyl)piperazin-1-ium 4-iodobenzoate (III) Data collection

**Table:**

Oxford Diffraction Xcalibur with Sapphire CCD diffractometer	3897 independent reflections
Radiation source: Enhance (Mo) X-ray Source	3203 reflections with *I* > 2σ(*I*)
Graphite monochromator	*R*_int_ = 0.012
ω scans	θ_max_ = 28.0°, θ_min_ = 3.0°
Absorption correction: multi-scan (CrysAlis RED; Oxford Diffraction, 2009)	*h* = −6→9
*T*_min_ = 0.534, *T*_max_ = 0.779	*k* = −14→14
6342 measured reflections	*l* = −16→16

### 4-(2-Methoxyphenyl)piperazin-1-ium 4-iodobenzoate (III) Refinement

**Table:**

Refinement on *F*^2^	Primary atom site location: difference Fourier map
Least-squares matrix: full	Hydrogen site location: mixed
*R*[*F*^2^ > 2σ(*F*^2^)] = 0.026	H atoms treated by a mixture of independent and constrained refinement
*wR*(*F*^2^) = 0.065	*w* = 1/[σ^2^(*F*_o_^2^) + (0.029*P*)^2^ + 0.4404*P*] where *P* = (*F*_o_^2^ + 2*F*_c_^2^)/3
*S* = 1.02	(Δ/σ)_max_ = 0.003
3897 reflections	Δρ_max_ = 0.52 e Å^−^^3^
224 parameters	Δρ_min_ = −0.70 e Å^−^^3^
0 restraints	

### 4-(2-Methoxyphenyl)piperazin-1-ium 4-iodobenzoate (III) Special details

**Table:**

Experimental. Empirical absorption correction using spherical harmonics, implemented in SCALE3 ABSPACK scaling algorithm.
Geometry. All esds (except the esd in the dihedral angle between two l.s. planes) are estimated using the full covariance matrix. The cell esds are taken into account individually in the estimation of esds in distances, angles and torsion angles; correlations between esds in cell parameters are only used when they are defined by crystal symmetry. An approximate (isotropic) treatment of cell esds is used for estimating esds involving l.s. planes.

### 4-(2-Methoxyphenyl)piperazin-1-ium 4-iodobenzoate (III) Fractional atomic coordinates and isotropic or equivalent isotropic displacement parameters (Å^2^)

**Table:**

	*x*	*y*	*z*	*U*_iso_*/*U*_eq_	
N1	0.6379 (3)	0.34876 (17)	0.41481 (15)	0.0371 (4)	
H11	0.736 (3)	0.364 (2)	0.437 (2)	0.045*	
H12	0.545 (4)	0.417 (2)	0.405 (2)	0.045*	
C2	0.5540 (3)	0.2342 (2)	0.50290 (17)	0.0393 (4)	
H2A	0.4957	0.2524	0.5752	0.047*	
H2B	0.6580	0.1651	0.5168	0.047*	
C3	0.4004 (3)	0.1942 (2)	0.46316 (17)	0.0405 (4)	
H3A	0.3499	0.1181	0.5208	0.049*	
H3B	0.2923	0.2609	0.4536	0.049*	
N4	0.4884 (2)	0.16937 (15)	0.35326 (13)	0.0345 (3)	
C5	0.5578 (3)	0.2859 (2)	0.26462 (17)	0.0414 (5)	
H5A	0.4490	0.3523	0.2555	0.050*	
H5B	0.6108	0.2700	0.1909	0.050*	
C6	0.7146 (3)	0.3286 (2)	0.30021 (18)	0.0430 (5)	
H6A	0.8271	0.2646	0.3039	0.052*	
H6B	0.7570	0.4071	0.2428	0.052*	
C21	0.3825 (3)	0.10145 (18)	0.31654 (17)	0.0354 (4)	
C22	0.4854 (3)	0.0392 (2)	0.23523 (18)	0.0420 (5)	
C23	0.3903 (4)	−0.0354 (2)	0.2031 (2)	0.0544 (6)	
H23	0.4592	−0.0769	0.1499	0.065*	
C24	0.1939 (4)	−0.0483 (2)	0.2498 (2)	0.0605 (7)	
H24	0.1313	−0.0995	0.2288	0.073*	
C25	0.0906 (4)	0.0141 (3)	0.3269 (2)	0.0594 (7)	
H25	−0.0424	0.0064	0.3570	0.071*	
C26	0.1844 (3)	0.0888 (2)	0.3602 (2)	0.0465 (5)	
H26	0.1132	0.1309	0.4126	0.056*	
O22	0.6793 (2)	0.05587 (18)	0.19524 (16)	0.0594 (5)	
C27	0.7839 (4)	0.0197 (3)	0.0974 (2)	0.0694 (8)	
H27A	0.9125	0.0472	0.0736	0.104*	
H27B	0.7156	0.0583	0.0354	0.104*	
H27C	0.7949	−0.0711	0.1166	0.104*	
C31	0.9029 (3)	0.37755 (18)	0.70670 (17)	0.0344 (4)	
C32	1.1047 (3)	0.3626 (2)	0.69034 (18)	0.0395 (4)	
H32	1.1812	0.3648	0.6174	0.047*	
C33	1.1957 (3)	0.3443 (2)	0.78032 (18)	0.0411 (5)	
H33	1.3317	0.3347	0.7681	0.049*	
C34	1.0812 (3)	0.34062 (19)	0.88832 (17)	0.0405 (5)	
I34	1.21476 (3)	0.30958 (2)	1.02693 (2)	0.07326 (9)	
C35	0.8796 (4)	0.3559 (2)	0.90646 (19)	0.0528 (6)	
H35	0.8034	0.3533	0.9795	0.063*	
C36	0.7913 (3)	0.3751 (2)	0.8158 (2)	0.0469 (5)	
H36	0.6552	0.3865	0.8279	0.056*	
C37	0.8053 (3)	0.39540 (18)	0.60869 (18)	0.0382 (4)	
O31	0.9072 (2)	0.36274 (15)	0.52331 (13)	0.0466 (3)	
O32	0.6270 (2)	0.43759 (15)	0.62045 (15)	0.0520 (4)	

### 4-(2-Methoxyphenyl)piperazin-1-ium 4-iodobenzoate (III) Atomic displacement parameters (Å^2^)

**Table:**

	*U*^11^	*U*^22^	*U*^33^	*U*^12^	*U*^13^	*U*^23^
N1	0.0383 (9)	0.0379 (9)	0.0422 (9)	0.0006 (7)	−0.0142 (7)	−0.0195 (7)
C2	0.0428 (11)	0.0447 (11)	0.0327 (10)	−0.0010 (9)	−0.0109 (8)	−0.0150 (8)
C3	0.0397 (11)	0.0504 (12)	0.0336 (10)	−0.0068 (9)	−0.0035 (8)	−0.0182 (9)
N4	0.0372 (8)	0.0381 (9)	0.0310 (8)	−0.0066 (7)	−0.0049 (7)	−0.0147 (7)
C5	0.0568 (13)	0.0390 (11)	0.0309 (10)	−0.0104 (9)	−0.0090 (9)	−0.0118 (8)
C6	0.0477 (12)	0.0423 (12)	0.0398 (11)	−0.0116 (9)	−0.0019 (9)	−0.0160 (9)
C21	0.0393 (10)	0.0338 (10)	0.0348 (9)	−0.0033 (8)	−0.0131 (8)	−0.0095 (8)
C22	0.0502 (12)	0.0396 (11)	0.0415 (11)	−0.0021 (9)	−0.0174 (9)	−0.0151 (9)
C23	0.0783 (17)	0.0410 (12)	0.0566 (14)	−0.0021 (11)	−0.0329 (13)	−0.0197 (10)
C24	0.0796 (19)	0.0472 (14)	0.0684 (16)	−0.0216 (13)	−0.0396 (15)	−0.0095 (12)
C25	0.0530 (14)	0.0625 (16)	0.0630 (15)	−0.0234 (12)	−0.0246 (12)	−0.0021 (12)
C26	0.0418 (12)	0.0507 (13)	0.0459 (12)	−0.0068 (10)	−0.0123 (9)	−0.0105 (10)
O22	0.0477 (9)	0.0821 (12)	0.0644 (11)	−0.0033 (8)	−0.0030 (8)	−0.0515 (10)
C27	0.0792 (19)	0.0738 (18)	0.0601 (16)	−0.0034 (15)	0.0021 (14)	−0.0420 (14)
C31	0.0364 (10)	0.0296 (9)	0.0380 (10)	−0.0024 (8)	−0.0091 (8)	−0.0114 (8)
C32	0.0393 (11)	0.0443 (11)	0.0350 (10)	−0.0024 (9)	−0.0068 (8)	−0.0146 (9)
C33	0.0371 (11)	0.0426 (11)	0.0443 (11)	−0.0024 (9)	−0.0133 (9)	−0.0121 (9)
C34	0.0551 (13)	0.0344 (10)	0.0342 (10)	−0.0054 (9)	−0.0167 (9)	−0.0079 (8)
I34	0.09118 (16)	0.08904 (16)	0.04736 (11)	−0.00653 (11)	−0.03522 (9)	−0.01745 (9)
C35	0.0542 (14)	0.0662 (15)	0.0340 (11)	−0.0080 (12)	0.0000 (10)	−0.0171 (10)
C36	0.0369 (11)	0.0584 (14)	0.0439 (12)	−0.0037 (10)	−0.0036 (9)	−0.0184 (10)
C37	0.0392 (11)	0.0319 (10)	0.0461 (11)	−0.0028 (8)	−0.0159 (9)	−0.0110 (9)
O31	0.0426 (8)	0.0614 (10)	0.0433 (8)	−0.0034 (7)	−0.0137 (7)	−0.0232 (7)
O32	0.0399 (8)	0.0546 (9)	0.0664 (10)	0.0106 (7)	−0.0220 (7)	−0.0254 (8)

### 4-(2-Methoxyphenyl)piperazin-1-ium 4-iodobenzoate (III) Geometric parameters (Å, º)

**Table:**

N1—C6	1.482 (3)	C24—C25	1.369 (4)
N1—C2	1.486 (3)	C24—H24	0.9300
N1—H11	0.88 (2)	C25—C26	1.391 (3)
N1—H12	0.91 (2)	C25—H25	0.9300
C2—C3	1.510 (3)	C26—H26	0.9300
C2—H2A	0.9700	O22—C27	1.409 (3)
C2—H2B	0.9700	C27—H27A	0.9600
C3—N4	1.459 (2)	C27—H27B	0.9600
C3—H3A	0.9700	C27—H27C	0.9600
C3—H3B	0.9700	C31—C32	1.381 (3)
N4—C21	1.419 (2)	C31—C36	1.387 (3)
N4—C5	1.469 (3)	C31—C37	1.508 (3)
C5—C6	1.514 (3)	C32—C33	1.386 (3)
C5—H5A	0.9700	C32—H32	0.9300
C5—H5B	0.9700	C33—C34	1.379 (3)
C6—H6A	0.9700	C33—H33	0.9300
C6—H6B	0.9700	C34—C35	1.378 (3)
C21—C26	1.385 (3)	C34—I34	2.098 (2)
C21—C22	1.407 (3)	C35—C36	1.379 (3)
C22—O22	1.363 (3)	C35—H35	0.9300
C22—C23	1.387 (3)	C36—H36	0.9300
C23—C24	1.379 (4)	C37—O31	1.256 (3)
C23—H23	0.9300	C37—O32	1.256 (2)
			
C6—N1—C2	111.24 (15)	C24—C23—H23	119.9
C6—N1—H11	108.4 (15)	C22—C23—H23	119.9
C2—N1—H11	108.5 (15)	C25—C24—C23	120.2 (2)
C6—N1—H12	106.8 (15)	C25—C24—H24	119.9
C2—N1—H12	110.9 (15)	C23—C24—H24	119.9
H11—N1—H12	111 (2)	C24—C25—C26	120.1 (2)
N1—C2—C3	111.04 (16)	C24—C25—H25	120.0
N1—C2—H2A	109.4	C26—C25—H25	120.0
C3—C2—H2A	109.4	C21—C26—C25	121.0 (2)
N1—C2—H2B	109.4	C21—C26—H26	119.5
C3—C2—H2B	109.4	C25—C26—H26	119.5
H2A—C2—H2B	108.0	C22—O22—C27	119.10 (19)
N4—C3—C2	109.09 (16)	O22—C27—H27A	109.5
N4—C3—H3A	109.9	O22—C27—H27B	109.5
C2—C3—H3A	109.9	H27A—C27—H27B	109.5
N4—C3—H3B	109.9	O22—C27—H27C	109.5
C2—C3—H3B	109.9	H27A—C27—H27C	109.5
H3A—C3—H3B	108.3	H27B—C27—H27C	109.5
C21—N4—C3	117.03 (15)	C32—C31—C36	118.49 (19)
C21—N4—C5	114.74 (15)	C32—C31—C37	120.99 (18)
C3—N4—C5	109.97 (16)	C36—C31—C37	120.52 (18)
N4—C5—C6	109.74 (17)	C31—C32—C33	121.39 (19)
N4—C5—H5A	109.7	C31—C32—H32	119.3
C6—C5—H5A	109.7	C33—C32—H32	119.3
N4—C5—H5B	109.7	C34—C33—C32	118.95 (19)
C6—C5—H5B	109.7	C34—C33—H33	120.5
H5A—C5—H5B	108.2	C32—C33—H33	120.5
N1—C6—C5	110.39 (17)	C35—C34—C33	120.63 (19)
N1—C6—H6A	109.6	C35—C34—I34	119.56 (15)
C5—C6—H6A	109.6	C33—C34—I34	119.81 (16)
N1—C6—H6B	109.6	C34—C35—C36	119.7 (2)
C5—C6—H6B	109.6	C34—C35—H35	120.1
H6A—C6—H6B	108.1	C36—C35—H35	120.1
C26—C21—C22	118.24 (19)	C35—C36—C31	120.8 (2)
C26—C21—N4	123.33 (18)	C35—C36—H36	119.6
C22—C21—N4	118.37 (17)	C31—C36—H36	119.6
O22—C22—C23	124.4 (2)	O31—C37—O32	125.09 (19)
O22—C22—C21	115.38 (17)	O31—C37—C31	117.28 (17)
C23—C22—C21	120.2 (2)	O32—C37—C31	117.58 (18)
C24—C23—C22	120.3 (2)		
			
C6—N1—C2—C3	−54.5 (2)	C22—C21—C26—C25	1.6 (3)
N1—C2—C3—N4	58.0 (2)	N4—C21—C26—C25	−175.6 (2)
C2—C3—N4—C21	164.91 (17)	C24—C25—C26—C21	−0.1 (4)
C2—C3—N4—C5	−61.8 (2)	C23—C22—O22—C27	14.5 (4)
C21—N4—C5—C6	−163.68 (17)	C21—C22—O22—C27	−167.1 (2)
C3—N4—C5—C6	61.9 (2)	C36—C31—C32—C33	0.6 (3)
C2—N1—C6—C5	53.9 (2)	C37—C31—C32—C33	−179.05 (19)
N4—C5—C6—N1	−57.3 (2)	C31—C32—C33—C34	0.2 (3)
C3—N4—C21—C26	18.2 (3)	C32—C33—C34—C35	−0.5 (3)
C5—N4—C21—C26	−112.9 (2)	C32—C33—C34—I34	178.83 (15)
C3—N4—C21—C22	−159.00 (18)	C33—C34—C35—C36	0.0 (4)
C5—N4—C21—C22	69.9 (2)	I34—C34—C35—C36	−179.37 (18)
C26—C21—C22—O22	179.72 (19)	C34—C35—C36—C31	0.9 (4)
N4—C21—C22—O22	−2.9 (3)	C32—C31—C36—C35	−1.1 (3)
C26—C21—C22—C23	−1.8 (3)	C37—C31—C36—C35	178.5 (2)
N4—C21—C22—C23	175.53 (19)	C32—C31—C37—O31	18.7 (3)
O22—C22—C23—C24	178.9 (2)	C36—C31—C37—O31	−161.0 (2)
C21—C22—C23—C24	0.6 (3)	C32—C31—C37—O32	−163.71 (19)
C22—C23—C24—C25	1.0 (4)	C36—C31—C37—O32	16.7 (3)
C23—C24—C25—C26	−1.2 (4)		

### 4-(2-Methoxyphenyl)piperazin-1-ium 4-iodobenzoate (III) Hydrogen-bond geometry (Å, º)

**Table:**

*D*—H···*A*	*D*—H	H···*A*	*D*···*A*	*D*—H···*A*
N1—H11···O31	0.88 (2)	1.83 (2)	2.684 (3)	163 (2)
N1—H11···O32	0.88 (2)	2.60 (2)	3.060 (3)	113.6 (17)
N1—H12···O32^i^	0.91 (3)	1.84 (3)	2.746 (3)	176 (3)
C33—H33···O32^ii^	0.93	2.57	3.327 (3)	139
C2—H2*B*···*Cg*2^iii^	0.97	2.77	3.482 (2)	131

Symmetry codes: (i) −*x*+1, −*y*+1, −*z*+1; (ii) *x*+1, *y*, *z*; (iii) −*x*+1, −*y*, −*z*+1.

### 4-(2-Methoxyphenyl)piperazin-1-ium 2-fluorobenzoate (IV) Crystal data

**Table:**

C_11_H_17_N_2_O^+^·C_7_H_4_FO_2_^−^	*F*(000) = 704
*M**_r_* = 332.37	*D*_x_ = 1.270 Mg m^−^^3^
Monoclinic, *C**c*	Mo *K*α radiation, λ = 0.71073 Å
*a* = 19.940 (1) Å	Cell parameters from 3343 reflections
*b* = 10.2705 (7) Å	θ = 3.0–27.9°
*c* = 9.0148 (7) Å	µ = 0.09 mm^−^^1^
β = 109.663 (8)°	*T* = 296 K
*V* = 1738.5 (2) Å^3^	Block, orange
*Z* = 4	0.48 × 0.36 × 0.22 mm

### 4-(2-Methoxyphenyl)piperazin-1-ium 2-fluorobenzoate (IV) Data collection

**Table:**

Oxford Diffraction Xcalibur with Sapphire CCD diffractometer	3343 independent reflections
Radiation source: Enhance (Mo) X-ray Source	2786 reflections with *I* > 2σ(*I*)
Graphite monochromator	*R*_int_ = 0.012
ω scans	θ_max_ = 27.9°, θ_min_ = 3.0°
Absorption correction: multi-scan (CrysAlis RED; Oxford Diffraction, 2009)	*h* = −25→25
*T*_min_ = 0.884, *T*_max_ = 0.963	*k* = −13→13
6204 measured reflections	*l* = −11→11

### 4-(2-Methoxyphenyl)piperazin-1-ium 2-fluorobenzoate (IV) Refinement

**Table:**

Refinement on *F*^2^	H atoms treated by a mixture of independent and constrained refinement
Least-squares matrix: full	*w* = 1/[σ^2^(*F*_o_^2^) + (0.0593*P*)^2^ + 0.1911*P*] where *P* = (*F*_o_^2^ + 2*F*_c_^2^)/3
*R*[*F*^2^ > 2σ(*F*^2^)] = 0.036	(Δ/σ)_max_ < 0.001
*wR*(*F*^2^) = 0.100	Δρ_max_ = 0.24 e Å^−^^3^
*S* = 1.02	Δρ_min_ = −0.14 e Å^−^^3^
3343 reflections	Extinction correction: SHELXL, Fc^*^=kFc[1+0.001xFc^2^λ^3^/sin(2θ)]^-1/4^
256 parameters	Extinction coefficient: 0.0111 (16)
25 restraints	Absolute structure: Flack *x* determined using 1089 quotients [(*I*^+^)-(*I*^-^)]/[(*I*^+^)+(*I*^-^)] (Parsons *et al.*, 2013)
Primary atom site location: difference Fourier map	Absolute structure parameter: 0.2 (3)
Hydrogen site location: mixed	

### 4-(2-Methoxyphenyl)piperazin-1-ium 2-fluorobenzoate (IV) Special details

**Table:**

Experimental. Empirical absorption correction using spherical harmonics, implemented in SCALE3 ABSPACK scaling algorithm.
Geometry. All esds (except the esd in the dihedral angle between two l.s. planes) are estimated using the full covariance matrix. The cell esds are taken into account individually in the estimation of esds in distances, angles and torsion angles; correlations between esds in cell parameters are only used when they are defined by crystal symmetry. An approximate (isotropic) treatment of cell esds is used for estimating esds involving l.s. planes.

### 4-(2-Methoxyphenyl)piperazin-1-ium 2-fluorobenzoate (IV) Fractional atomic coordinates and isotropic or equivalent isotropic displacement parameters (Å^2^)

**Table:**

	*x*	*y*	*z*	*U*_iso_*/*U*_eq_	Occ. (<1)
N1	0.40896 (13)	0.4278 (2)	0.1329 (3)	0.0536 (5)	
H11	0.3741 (17)	0.499 (3)	0.119 (3)	0.064*	
H12	0.4026 (16)	0.383 (3)	0.046 (4)	0.064*	
C2	0.39924 (15)	0.3361 (3)	0.2509 (3)	0.0554 (6)	
H2A	0.3543	0.2906	0.2060	0.067*	
H2B	0.3975	0.3846	0.3419	0.067*	
C3	0.45902 (15)	0.2383 (3)	0.3025 (4)	0.0564 (6)	
H3A	0.4530	0.1831	0.3844	0.068*	
H3B	0.4579	0.1834	0.2140	0.068*	
N4	0.52785 (12)	0.30576 (19)	0.3622 (3)	0.0482 (5)	
C5	0.53817 (15)	0.3831 (3)	0.2368 (3)	0.0553 (6)	
H5A	0.5357	0.3275	0.1481	0.066*	
H5B	0.5848	0.4237	0.2737	0.066*	
C6	0.48133 (16)	0.4861 (3)	0.1862 (3)	0.0569 (7)	
H6A	0.4861	0.5445	0.2737	0.068*	
H6B	0.4877	0.5367	0.1011	0.068*	
C21	0.58610 (13)	0.2258 (2)	0.4497 (3)	0.0476 (6)	
C22	0.58740 (14)	0.1743 (3)	0.5946 (3)	0.0511 (6)	
C23	0.64515 (17)	0.1007 (3)	0.6840 (4)	0.0635 (7)	
H23	0.6453	0.0653	0.7791	0.076*	
C24	0.70234 (17)	0.0792 (3)	0.6336 (4)	0.0702 (8)	
H24	0.7407	0.0296	0.6946	0.084*	
C25	0.70252 (16)	0.1304 (3)	0.4950 (4)	0.0659 (8)	
H25	0.7414	0.1168	0.4618	0.079*	
C26	0.64492 (14)	0.2029 (3)	0.4028 (3)	0.0561 (6)	
H26	0.6455	0.2369	0.3077	0.067*	
O22	0.53102 (11)	0.2053 (2)	0.6414 (2)	0.0672 (6)	
C27	0.5288 (2)	0.1521 (5)	0.7843 (5)	0.1014 (14)	
H27A	0.5278	0.0588	0.7775	0.152*	
H27B	0.4868	0.1822	0.8031	0.152*	
H27C	0.5702	0.1791	0.8694	0.152*	
C31	0.30015 (19)	0.8223 (3)	0.2163 (4)	0.0505 (8)	0.907 (8)
C32	0.3262 (2)	0.9222 (4)	0.3203 (5)	0.0663 (12)	0.907 (8)
F32	0.39194 (19)	0.9110 (3)	0.4272 (4)	0.1101 (11)	0.907 (8)
C33	0.2910 (3)	1.0368 (4)	0.3207 (6)	0.0868 (15)	0.907 (8)
H33	0.3118	1.1029	0.3919	0.104*	0.907 (8)
C34	0.2242 (3)	1.0503 (5)	0.2129 (6)	0.0952 (19)	0.907 (8)
H34	0.1988	1.1265	0.2108	0.114*	0.907 (8)
C35	0.1943 (3)	0.9527 (7)	0.1077 (6)	0.0984 (19)	0.907 (8)
H35	0.1487	0.9633	0.0356	0.118*	0.907 (8)
C36	0.2312 (2)	0.8384 (5)	0.1072 (5)	0.0735 (12)	0.907 (8)
H36	0.2105	0.7728	0.0352	0.088*	0.907 (8)
C37	0.34049 (18)	0.6986 (3)	0.2127 (4)	0.0511 (9)	0.907 (8)
O31	0.3266 (2)	0.6424 (3)	0.0810 (3)	0.0663 (9)	0.907 (8)
O32	0.38350 (17)	0.6562 (4)	0.3377 (4)	0.0779 (12)	0.907 (8)
C41	0.2765 (15)	0.794 (3)	0.179 (4)	0.0505 (8)	0.093 (8)
C42	0.2927 (19)	0.900 (3)	0.276 (4)	0.0663 (12)	0.093 (8)
F42	0.3612 (19)	0.929 (4)	0.357 (6)	0.1101 (11)	0.093 (8)
C43	0.248 (2)	1.005 (4)	0.262 (6)	0.0868 (15)	0.093 (8)
H43	0.2599	1.0754	0.3298	0.104*	0.093 (8)
C44	0.184 (3)	0.997 (4)	0.142 (6)	0.0952 (19)	0.093 (8)
H44	0.1518	1.0657	0.1284	0.114*	0.093 (8)
C45	0.166 (2)	0.894 (4)	0.042 (5)	0.0984 (19)	0.093 (8)
H45	0.1219	0.8954	−0.0390	0.118*	0.093 (8)
C46	0.2094 (17)	0.786 (4)	0.054 (4)	0.0735 (12)	0.093 (8)
H46	0.1963	0.7151	−0.0135	0.088*	0.093 (8)
C47	0.3262 (19)	0.679 (3)	0.198 (4)	0.0511 (9)	0.093 (8)
O41	0.3491 (19)	0.664 (4)	0.084 (4)	0.0663 (9)	0.093 (8)
O42	0.359 (2)	0.640 (5)	0.334 (4)	0.0779 (12)	0.093 (8)

### 4-(2-Methoxyphenyl)piperazin-1-ium 2-fluorobenzoate (IV) Atomic displacement parameters (Å^2^)

**Table:**

	*U*^11^	*U*^22^	*U*^33^	*U*^12^	*U*^13^	*U*^23^
N1	0.0552 (13)	0.0560 (13)	0.0418 (11)	0.0094 (11)	0.0060 (9)	−0.0028 (9)
C2	0.0443 (13)	0.0595 (15)	0.0548 (14)	0.0023 (12)	0.0065 (11)	0.0043 (12)
C3	0.0451 (13)	0.0491 (14)	0.0664 (15)	−0.0031 (11)	0.0074 (11)	0.0015 (13)
N4	0.0420 (10)	0.0461 (11)	0.0512 (11)	−0.0012 (9)	0.0086 (8)	0.0033 (9)
C5	0.0546 (15)	0.0621 (15)	0.0475 (13)	−0.0023 (13)	0.0151 (11)	0.0010 (12)
C6	0.0664 (17)	0.0532 (15)	0.0472 (13)	0.0001 (13)	0.0139 (12)	0.0060 (12)
C21	0.0434 (13)	0.0395 (12)	0.0529 (13)	0.0002 (11)	0.0070 (10)	−0.0050 (11)
C22	0.0458 (13)	0.0413 (13)	0.0625 (15)	0.0009 (10)	0.0134 (11)	0.0024 (11)
C23	0.0618 (17)	0.0523 (15)	0.0712 (17)	0.0109 (13)	0.0154 (13)	0.0138 (14)
C24	0.0528 (16)	0.0609 (17)	0.083 (2)	0.0184 (14)	0.0049 (14)	0.0046 (16)
C25	0.0456 (15)	0.0724 (19)	0.0751 (18)	0.0090 (14)	0.0142 (13)	−0.0172 (16)
C26	0.0470 (14)	0.0636 (16)	0.0541 (14)	0.0009 (12)	0.0121 (11)	−0.0126 (13)
O22	0.0646 (12)	0.0695 (13)	0.0737 (13)	0.0176 (10)	0.0314 (10)	0.0251 (10)
C27	0.102 (3)	0.109 (3)	0.115 (3)	0.029 (2)	0.064 (2)	0.052 (3)
C31	0.0524 (19)	0.0539 (18)	0.0516 (18)	0.0091 (15)	0.0257 (15)	0.0149 (15)
C32	0.061 (3)	0.075 (2)	0.069 (3)	0.014 (2)	0.030 (3)	0.0084 (19)
F32	0.098 (2)	0.119 (2)	0.098 (2)	0.0153 (17)	0.0134 (17)	−0.0298 (18)
C33	0.118 (4)	0.071 (2)	0.093 (3)	0.019 (3)	0.064 (3)	0.005 (2)
C34	0.121 (5)	0.089 (3)	0.100 (4)	0.052 (3)	0.069 (4)	0.040 (3)
C35	0.083 (3)	0.134 (5)	0.088 (4)	0.058 (3)	0.042 (3)	0.055 (3)
C36	0.062 (2)	0.093 (3)	0.071 (3)	0.028 (2)	0.0298 (19)	0.034 (2)
C37	0.0469 (19)	0.0538 (18)	0.0535 (16)	0.0080 (15)	0.0180 (13)	0.0166 (14)
O31	0.064 (2)	0.0682 (17)	0.0588 (12)	0.0137 (15)	0.0102 (14)	0.0001 (12)
O32	0.092 (3)	0.087 (2)	0.0537 (12)	0.041 (2)	0.0226 (16)	0.0226 (12)
C41	0.0524 (19)	0.0539 (18)	0.0516 (18)	0.0091 (15)	0.0257 (15)	0.0149 (15)
C42	0.061 (3)	0.075 (2)	0.069 (3)	0.014 (2)	0.030 (3)	0.0084 (19)
F42	0.098 (2)	0.119 (2)	0.098 (2)	0.0153 (17)	0.0134 (17)	−0.0298 (18)
C43	0.118 (4)	0.071 (2)	0.093 (3)	0.019 (3)	0.064 (3)	0.005 (2)
C44	0.121 (5)	0.089 (3)	0.100 (4)	0.052 (3)	0.069 (4)	0.040 (3)
C45	0.083 (3)	0.134 (5)	0.088 (4)	0.058 (3)	0.042 (3)	0.055 (3)
C46	0.062 (2)	0.093 (3)	0.071 (3)	0.028 (2)	0.0298 (19)	0.034 (2)
C47	0.0469 (19)	0.0538 (18)	0.0535 (16)	0.0080 (15)	0.0180 (13)	0.0166 (14)
O41	0.064 (2)	0.0682 (17)	0.0588 (12)	0.0137 (15)	0.0102 (14)	0.0001 (12)
O42	0.092 (3)	0.087 (2)	0.0537 (12)	0.041 (2)	0.0226 (16)	0.0226 (12)

### 4-(2-Methoxyphenyl)piperazin-1-ium 2-fluorobenzoate (IV) Geometric parameters (Å, º)

**Table:**

N1—C2	1.482 (4)	C27—H27B	0.9600
N1—C6	1.485 (4)	C27—H27C	0.9600
N1—H11	0.98 (3)	C31—C32	1.369 (6)
N1—H12	0.88 (4)	C31—C36	1.405 (5)
C2—C3	1.508 (4)	C31—C37	1.510 (4)
C2—H2A	0.9700	C32—F32	1.345 (5)
C2—H2B	0.9700	C32—C33	1.371 (5)
C3—N4	1.468 (3)	C33—C34	1.366 (7)
C3—H3A	0.9700	C33—H33	0.9300
C3—H3B	0.9700	C34—C35	1.370 (8)
N4—C21	1.424 (3)	C34—H34	0.9300
N4—C5	1.452 (3)	C35—C36	1.387 (7)
C5—C6	1.504 (4)	C35—H35	0.9300
C5—H5A	0.9700	C36—H36	0.9300
C5—H5B	0.9700	C37—O32	1.243 (3)
C6—H6A	0.9700	C37—O31	1.264 (4)
C6—H6B	0.9700	C41—C42	1.365 (13)
C21—C26	1.394 (4)	C41—C46	1.432 (13)
C21—C22	1.402 (4)	C41—C47	1.518 (12)
C22—O22	1.365 (3)	C42—F42	1.347 (14)
C22—C23	1.387 (4)	C42—C43	1.376 (13)
C23—C24	1.380 (5)	C43—C44	1.364 (15)
C23—H23	0.9300	C43—H43	0.9300
C24—C25	1.357 (5)	C44—C45	1.365 (15)
C24—H24	0.9300	C44—H44	0.9300
C25—C26	1.386 (4)	C45—C46	1.388 (14)
C25—H25	0.9300	C45—H45	0.9300
C26—H26	0.9300	C46—H46	0.9300
O22—C27	1.414 (4)	C47—O42	1.245 (13)
C27—H27A	0.9600	C47—O41	1.263 (13)
			
C2—N1—C6	111.80 (18)	C21—C26—H26	119.3
C2—N1—H11	107.6 (17)	C22—O22—C27	118.3 (2)
C6—N1—H11	108.1 (18)	O22—C27—H27A	109.5
C2—N1—H12	107 (2)	O22—C27—H27B	109.5
C6—N1—H12	109 (2)	H27A—C27—H27B	109.5
H11—N1—H12	113 (3)	O22—C27—H27C	109.5
N1—C2—C3	111.3 (2)	H27A—C27—H27C	109.5
N1—C2—H2A	109.4	H27B—C27—H27C	109.5
C3—C2—H2A	109.4	C32—C31—C36	116.6 (3)
N1—C2—H2B	109.4	C32—C31—C37	124.3 (3)
C3—C2—H2B	109.4	C36—C31—C37	119.1 (3)
H2A—C2—H2B	108.0	F32—C32—C31	118.7 (3)
N4—C3—C2	110.1 (2)	F32—C32—C33	116.6 (4)
N4—C3—H3A	109.6	C31—C32—C33	124.7 (4)
C2—C3—H3A	109.6	C34—C33—C32	117.5 (5)
N4—C3—H3B	109.6	C34—C33—H33	121.2
C2—C3—H3B	109.6	C32—C33—H33	121.2
H3A—C3—H3B	108.2	C33—C34—C35	120.8 (4)
C21—N4—C5	116.3 (2)	C33—C34—H34	119.6
C21—N4—C3	114.73 (19)	C35—C34—H34	119.6
C5—N4—C3	109.4 (2)	C34—C35—C36	120.9 (4)
N4—C5—C6	109.3 (2)	C34—C35—H35	119.6
N4—C5—H5A	109.8	C36—C35—H35	119.6
C6—C5—H5A	109.8	C35—C36—C31	119.5 (5)
N4—C5—H5B	109.8	C35—C36—H36	120.2
C6—C5—H5B	109.8	C31—C36—H36	120.2
H5A—C5—H5B	108.3	O32—C37—O31	123.9 (3)
N1—C6—C5	111.5 (2)	O32—C37—C31	119.0 (3)
N1—C6—H6A	109.3	O31—C37—C31	117.0 (3)
C5—C6—H6A	109.3	C42—C41—C46	120.5 (14)
N1—C6—H6B	109.3	C42—C41—C47	123.0 (16)
C5—C6—H6B	109.3	C46—C41—C47	116.5 (15)
H6A—C6—H6B	108.0	F42—C42—C41	120.1 (19)
C26—C21—C22	117.7 (2)	F42—C42—C43	113 (2)
C26—C21—N4	122.8 (2)	C41—C42—C43	123.7 (16)
C22—C21—N4	119.3 (2)	C44—C43—C42	115.8 (17)
O22—C22—C23	123.8 (3)	C44—C43—H43	122.1
O22—C22—C21	116.2 (2)	C42—C43—H43	122.1
C23—C22—C21	119.9 (2)	C43—C44—C45	122.5 (17)
C24—C23—C22	120.8 (3)	C43—C44—H44	118.8
C24—C23—H23	119.6	C45—C44—H44	118.8
C22—C23—H23	119.6	C44—C45—C46	123.1 (17)
C25—C24—C23	120.0 (3)	C44—C45—H45	118.4
C25—C24—H24	120.0	C46—C45—H45	118.4
C23—C24—H24	120.0	C45—C46—C41	114.4 (16)
C24—C25—C26	120.1 (3)	C45—C46—H46	122.8
C24—C25—H25	119.9	C41—C46—H46	122.8
C26—C25—H25	119.9	O42—C47—O41	123 (2)
C25—C26—C21	121.4 (3)	O42—C47—C41	118.2 (19)
C25—C26—H26	119.3	O41—C47—C41	113.7 (19)
			
C6—N1—C2—C3	−50.7 (3)	C37—C31—C32—C33	177.5 (3)
N1—C2—C3—N4	55.7 (3)	F32—C32—C33—C34	179.8 (4)
C2—C3—N4—C21	165.0 (2)	C31—C32—C33—C34	2.2 (6)
C2—C3—N4—C5	−62.2 (3)	C32—C33—C34—C35	−0.6 (6)
C21—N4—C5—C6	−165.1 (2)	C33—C34—C35—C36	−0.5 (7)
C3—N4—C5—C6	62.9 (3)	C34—C35—C36—C31	0.1 (6)
C2—N1—C6—C5	51.9 (3)	C32—C31—C36—C35	1.3 (5)
N4—C5—C6—N1	−57.9 (3)	C37—C31—C36—C35	−178.8 (4)
C5—N4—C21—C26	−11.4 (3)	C32—C31—C37—O32	31.1 (5)
C3—N4—C21—C26	118.2 (3)	C36—C31—C37—O32	−148.9 (4)
C5—N4—C21—C22	164.2 (2)	C32—C31—C37—O31	−150.8 (4)
C3—N4—C21—C22	−66.3 (3)	C36—C31—C37—O31	29.3 (5)
C26—C21—C22—O22	176.1 (2)	C46—C41—C42—F42	−159 (5)
N4—C21—C22—O22	0.3 (3)	C47—C41—C42—F42	23 (6)
C26—C21—C22—C23	−1.6 (3)	C46—C41—C42—C43	0 (6)
N4—C21—C22—C23	−177.4 (2)	C47—C41—C42—C43	−179 (4)
O22—C22—C23—C24	−176.3 (3)	F42—C42—C43—C44	160 (5)
C21—C22—C23—C24	1.3 (4)	C41—C42—C43—C44	0 (7)
C22—C23—C24—C25	0.0 (5)	C42—C43—C44—C45	0 (8)
C23—C24—C25—C26	−0.9 (4)	C43—C44—C45—C46	1 (9)
C24—C25—C26—C21	0.5 (4)	C44—C45—C46—C41	−2 (7)
C22—C21—C26—C25	0.8 (4)	C42—C41—C46—C45	1 (6)
N4—C21—C26—C25	176.4 (2)	C47—C41—C46—C45	180 (4)
C23—C22—O22—C27	−4.5 (5)	C42—C41—C47—O42	40 (6)
C21—C22—O22—C27	177.9 (3)	C46—C41—C47—O42	−138 (5)
C36—C31—C32—F32	179.9 (4)	C42—C41—C47—O41	−115 (4)
C37—C31—C32—F32	0.0 (5)	C46—C41—C47—O41	66 (5)
C36—C31—C32—C33	−2.6 (5)		

### 4-(2-Methoxyphenyl)piperazin-1-ium 2-fluorobenzoate (IV) Hydrogen-bond geometry (Å, º)

**Table:**

*D*—H···*A*	*D*—H	H···*A*	*D*···*A*	*D*—H···*A*
N1—H11···O31	0.99 (3)	1.72 (3)	2.694 (4)	167 (3)
N1—H11···O32	0.99 (3)	2.51 (3)	3.131 (4)	120.9 (19)
N1—H12···O32^i^	0.88 (3)	1.83 (3)	2.679 (4)	161 (3)
N1—H11···O41	0.99 (3)	1.77 (5)	2.67 (4)	151 (3)
N1—H11···O42	0.99 (3)	2.52 (5)	3.20 (4)	126 (2)
N1—H12···O42^i^	0.88 (3)	1.83 (5)	2.63 (4)	151 (3)
C34—H34···*Cg*2^ii^	0.93	2.74	3.543 (5)	145
C44—H44···*Cg*2^ii^	0.93	2.99	3.73 (4)	137
C26—H26···*Cg*3^iii^	0.93	2.96	3.754 (17)	144

Symmetry codes: (i) *x*, −*y*+1, *z*−1/2; (ii) *x*−1/2, −*y*+3/2, *z*−1/2; (iii) *x*+1/2, *y*−1/2, *z*.

### 4-(2-Methoxyphenyl)piperazin-1-ium 2-chlorobenzoate (V) Crystal data

**Table:**

C_11_H_17_N_2_O^+^·C_7_H_4_ClO_2_^−^	*F*(000) = 736
*M**_r_* = 348.82	*D*_x_ = 1.272 Mg m^−^^3^
Monoclinic, *P*2_1_/*c*	Mo *K*α radiation, λ = 0.71073 Å
*a* = 7.9974 (8) Å	Cell parameters from 4043 reflections
*b* = 27.611 (2) Å	θ = 2.6–28.0°
*c* = 8.5972 (9) Å	µ = 0.23 mm^−^^1^
β = 106.40 (1)°	*T* = 296 K
*V* = 1821.2 (3) Å^3^	Needle, orange
*Z* = 4	0.48 × 0.20 × 0.12 mm

### 4-(2-Methoxyphenyl)piperazin-1-ium 2-chlorobenzoate (V) Data collection

**Table:**

Oxford Diffraction Xcalibur with Sapphire CCD diffractometer	3410 independent reflections
Radiation source: Enhance (Mo) X-ray Source	2060 reflections with *I* > 2σ(*I*)
Graphite monochromator	*R*_int_ = 0.030
ω scans	θ_max_ = 25.6°, θ_min_ = 2.6°
Absorption correction: multi-scan (CrysAlis RED; Oxford Diffraction, 2009)	*h* = −9→9
*T*_min_ = 0.747, *T*_max_ = 0.973	*k* = −33→33
13275 measured reflections	*l* = −10→10

### 4-(2-Methoxyphenyl)piperazin-1-ium 2-chlorobenzoate (V) Refinement

**Table:**

Refinement on *F*^2^	Primary atom site location: difference Fourier map
Least-squares matrix: full	Hydrogen site location: mixed
*R*[*F*^2^ > 2σ(*F*^2^)] = 0.067	H atoms treated by a mixture of independent and constrained refinement
*wR*(*F*^2^) = 0.216	*w* = 1/[σ^2^(*F*_o_^2^) + (0.1138*P*)^2^ + 0.7483*P*] where *P* = (*F*_o_^2^ + 2*F*_c_^2^)/3
*S* = 1.03	(Δ/σ)_max_ < 0.001
3410 reflections	Δρ_max_ = 1.15 e Å^−^^3^
223 parameters	Δρ_min_ = −0.29 e Å^−^^3^
0 restraints	

### 4-(2-Methoxyphenyl)piperazin-1-ium 2-chlorobenzoate (V) Special details

**Table:**

Experimental. Empirical absorption correction using spherical harmonics, implemented in SCALE3 ABSPACK scaling algorithm.
Geometry. All esds (except the esd in the dihedral angle between two l.s. planes) are estimated using the full covariance matrix. The cell esds are taken into account individually in the estimation of esds in distances, angles and torsion angles; correlations between esds in cell parameters are only used when they are defined by crystal symmetry. An approximate (isotropic) treatment of cell esds is used for estimating esds involving l.s. planes.

### 4-(2-Methoxyphenyl)piperazin-1-ium 2-chlorobenzoate (V) Fractional atomic coordinates and isotropic or equivalent isotropic displacement parameters (Å^2^)

**Table:**

	*x*	*y*	*z*	*U*_iso_*/*U*_eq_	
N1	0.4620 (4)	0.44249 (11)	0.3578 (4)	0.0640 (8)	
H11	0.368 (5)	0.4525 (14)	0.402 (4)	0.077*	
H12	0.511 (5)	0.4709 (16)	0.336 (4)	0.077*	
C2	0.3954 (4)	0.41253 (14)	0.2112 (5)	0.0684 (10)	
H2A	0.3246	0.4323	0.1241	0.082*	
H2B	0.3225	0.3869	0.2333	0.082*	
C3	0.5449 (4)	0.39067 (13)	0.1593 (4)	0.0614 (9)	
H3A	0.4993	0.3699	0.0659	0.074*	
H3B	0.6122	0.4163	0.1284	0.074*	
N4	0.6572 (3)	0.36247 (10)	0.2916 (3)	0.0545 (7)	
C5	0.7325 (5)	0.39472 (14)	0.4289 (4)	0.0666 (10)	
H5A	0.7975	0.4204	0.3957	0.080*	
H5B	0.8118	0.3767	0.5159	0.080*	
C6	0.5886 (5)	0.41589 (16)	0.4871 (5)	0.0747 (11)	
H6A	0.5290	0.3901	0.5265	0.090*	
H6B	0.6379	0.4377	0.5769	0.090*	
C21	0.7769 (4)	0.33160 (12)	0.2452 (4)	0.0538 (8)	
C22	0.7097 (5)	0.29139 (12)	0.1470 (4)	0.0607 (9)	
C23	0.8208 (7)	0.26032 (14)	0.0999 (5)	0.0798 (12)	
H23	0.7765	0.2342	0.0326	0.096*	
C24	1.0003 (7)	0.26825 (19)	0.1536 (6)	0.0962 (16)	
H24	1.0751	0.2470	0.1223	0.115*	
C25	1.0676 (6)	0.30630 (18)	0.2506 (6)	0.0869 (13)	
H25	1.1875	0.3109	0.2868	0.104*	
C26	0.9558 (5)	0.33835 (14)	0.2954 (4)	0.0672 (10)	
H26	1.0018	0.3648	0.3602	0.081*	
O22	0.5338 (3)	0.28580 (9)	0.1060 (3)	0.0770 (8)	
C27	0.4570 (7)	0.24698 (17)	0.0025 (6)	0.1036 (16)	
H27A	0.3330	0.2476	−0.0157	0.155*	
H27B	0.4835	0.2502	−0.0992	0.155*	
H27C	0.5027	0.2168	0.0522	0.155*	
C31	0.1308 (4)	0.43466 (12)	0.7353 (4)	0.0541 (8)	
C32	0.1418 (4)	0.38566 (13)	0.7729 (4)	0.0606 (9)	
Cl32	0.29281 (16)	0.35006 (4)	0.71550 (13)	0.0871 (4)	
C33	0.0387 (6)	0.36423 (19)	0.8551 (5)	0.0886 (13)	
H33	0.0482	0.3312	0.8774	0.106*	
C34	−0.0776 (7)	0.3916 (3)	0.9036 (6)	0.1056 (17)	
H34	−0.1480	0.3772	0.9600	0.127*	
C35	−0.0935 (6)	0.4406 (2)	0.8710 (5)	0.0955 (15)	
H35	−0.1735	0.4591	0.9055	0.115*	
C36	0.0107 (5)	0.46182 (16)	0.7865 (5)	0.0760 (11)	
H36	0.0000	0.4948	0.7637	0.091*	
C37	0.2417 (5)	0.45733 (12)	0.6402 (5)	0.0643 (9)	
O31	0.1985 (3)	0.45110 (11)	0.4917 (3)	0.0810 (8)	
O32	0.3701 (5)	0.48041 (13)	0.7200 (4)	0.1152 (12)	

### 4-(2-Methoxyphenyl)piperazin-1-ium 2-chlorobenzoate (V) Atomic displacement parameters (Å^2^)

**Table:**

	*U*^11^	*U*^22^	*U*^33^	*U*^12^	*U*^13^	*U*^23^
N1	0.0672 (19)	0.0561 (18)	0.075 (2)	−0.0144 (15)	0.0306 (17)	−0.0193 (16)
C2	0.058 (2)	0.067 (2)	0.075 (2)	−0.0070 (17)	0.0105 (18)	−0.0203 (19)
C3	0.063 (2)	0.065 (2)	0.051 (2)	0.0015 (17)	0.0071 (16)	−0.0152 (16)
N4	0.0560 (16)	0.0569 (16)	0.0480 (15)	−0.0057 (12)	0.0104 (13)	−0.0120 (12)
C5	0.066 (2)	0.073 (2)	0.055 (2)	−0.0103 (18)	0.0071 (17)	−0.0133 (17)
C6	0.084 (3)	0.083 (3)	0.058 (2)	−0.012 (2)	0.022 (2)	−0.024 (2)
C21	0.060 (2)	0.0538 (18)	0.0486 (18)	−0.0018 (15)	0.0169 (15)	0.0073 (15)
C22	0.079 (2)	0.0516 (19)	0.057 (2)	0.0033 (18)	0.0281 (18)	0.0030 (16)
C23	0.113 (4)	0.056 (2)	0.082 (3)	0.013 (2)	0.045 (3)	0.0067 (19)
C24	0.113 (4)	0.080 (3)	0.114 (4)	0.045 (3)	0.062 (3)	0.038 (3)
C25	0.072 (3)	0.084 (3)	0.108 (3)	0.025 (2)	0.029 (2)	0.042 (3)
C26	0.061 (2)	0.067 (2)	0.067 (2)	0.0059 (18)	0.0090 (18)	0.0218 (18)
O22	0.0822 (18)	0.0675 (16)	0.0825 (18)	−0.0186 (13)	0.0251 (14)	−0.0303 (14)
C27	0.129 (4)	0.072 (3)	0.111 (4)	−0.036 (3)	0.037 (3)	−0.041 (3)
C31	0.0585 (19)	0.0577 (19)	0.0435 (17)	−0.0075 (15)	0.0100 (15)	0.0004 (14)
C32	0.072 (2)	0.062 (2)	0.0431 (18)	−0.0134 (17)	0.0088 (16)	0.0058 (15)
Cl32	0.1206 (9)	0.0538 (6)	0.0861 (8)	0.0079 (5)	0.0279 (6)	0.0040 (5)
C33	0.109 (4)	0.092 (3)	0.065 (3)	−0.020 (3)	0.026 (3)	0.020 (2)
C34	0.103 (4)	0.151 (5)	0.070 (3)	−0.021 (4)	0.036 (3)	0.033 (3)
C35	0.080 (3)	0.148 (5)	0.064 (3)	0.012 (3)	0.031 (2)	0.007 (3)
C36	0.076 (2)	0.086 (3)	0.069 (2)	0.007 (2)	0.025 (2)	0.004 (2)
C37	0.072 (2)	0.0407 (17)	0.085 (3)	−0.0034 (17)	0.030 (2)	0.0005 (18)
O31	0.0823 (18)	0.101 (2)	0.0683 (18)	0.0104 (15)	0.0350 (15)	0.0168 (15)
O32	0.130 (3)	0.094 (2)	0.132 (3)	−0.062 (2)	0.053 (2)	−0.024 (2)

### 4-(2-Methoxyphenyl)piperazin-1-ium 2-chlorobenzoate (V) Geometric parameters (Å, º)

**Table:**

N1—C6	1.472 (5)	C24—C25	1.355 (7)
N1—C2	1.475 (4)	C24—H24	0.9300
N1—H11	0.97 (4)	C25—C26	1.387 (6)
N1—H12	0.92 (4)	C25—H25	0.9300
C2—C3	1.515 (5)	C26—H26	0.9300
C2—H2A	0.9700	O22—C27	1.418 (4)
C2—H2B	0.9700	C27—H27A	0.9600
C3—N4	1.460 (4)	C27—H27B	0.9600
C3—H3A	0.9700	C27—H27C	0.9600
C3—H3B	0.9700	C31—C36	1.385 (5)
N4—C21	1.421 (4)	C31—C32	1.388 (5)
N4—C5	1.465 (4)	C31—C37	1.503 (5)
C5—C6	1.497 (5)	C32—C33	1.363 (5)
C5—H5A	0.9700	C32—Cl32	1.733 (4)
C5—H5B	0.9700	C33—C34	1.354 (7)
C6—H6A	0.9700	C33—H33	0.9300
C6—H6B	0.9700	C34—C35	1.378 (7)
C21—C26	1.385 (5)	C34—H34	0.9300
C21—C22	1.407 (5)	C35—C36	1.381 (6)
C22—O22	1.359 (4)	C35—H35	0.9300
C22—C23	1.375 (5)	C36—H36	0.9300
C23—C24	1.395 (6)	C37—O31	1.237 (5)
C23—H23	0.9300	C37—O32	1.238 (5)
			
C6—N1—C2	111.8 (3)	C22—C23—H23	120.2
C6—N1—H11	107 (2)	C24—C23—H23	120.2
C2—N1—H11	111 (2)	C25—C24—C23	121.2 (4)
C6—N1—H12	110 (2)	C25—C24—H24	119.4
C2—N1—H12	112 (2)	C23—C24—H24	119.4
H11—N1—H12	105 (3)	C24—C25—C26	119.4 (4)
N1—C2—C3	110.5 (3)	C24—C25—H25	120.3
N1—C2—H2A	109.5	C26—C25—H25	120.3
C3—C2—H2A	109.5	C21—C26—C25	121.2 (4)
N1—C2—H2B	109.5	C21—C26—H26	119.4
C3—C2—H2B	109.5	C25—C26—H26	119.4
H2A—C2—H2B	108.1	C22—O22—C27	118.8 (3)
N4—C3—C2	110.3 (3)	O22—C27—H27A	109.5
N4—C3—H3A	109.6	O22—C27—H27B	109.5
C2—C3—H3A	109.6	H27A—C27—H27B	109.5
N4—C3—H3B	109.6	O22—C27—H27C	109.5
C2—C3—H3B	109.6	H27A—C27—H27C	109.5
H3A—C3—H3B	108.1	H27B—C27—H27C	109.5
C21—N4—C3	114.6 (2)	C36—C31—C32	117.3 (3)
C21—N4—C5	115.8 (3)	C36—C31—C37	121.1 (3)
C3—N4—C5	109.0 (3)	C32—C31—C37	121.6 (3)
N4—C5—C6	109.1 (3)	C33—C32—C31	122.4 (4)
N4—C5—H5A	109.9	C33—C32—Cl32	118.2 (3)
C6—C5—H5A	109.9	C31—C32—Cl32	119.4 (3)
N4—C5—H5B	109.9	C34—C33—C32	119.1 (5)
C6—C5—H5B	109.9	C34—C33—H33	120.5
H5A—C5—H5B	108.3	C32—C33—H33	120.5
N1—C6—C5	111.8 (3)	C33—C34—C35	121.1 (4)
N1—C6—H6A	109.2	C33—C34—H34	119.4
C5—C6—H6A	109.2	C35—C34—H34	119.4
N1—C6—H6B	109.2	C34—C35—C36	119.3 (4)
C5—C6—H6B	109.2	C34—C35—H35	120.4
H6A—C6—H6B	107.9	C36—C35—H35	120.4
C26—C21—C22	118.6 (3)	C35—C36—C31	120.8 (4)
C26—C21—N4	123.4 (3)	C35—C36—H36	119.6
C22—C21—N4	118.0 (3)	C31—C36—H36	119.6
O22—C22—C23	124.1 (4)	O31—C37—O32	126.2 (4)
O22—C22—C21	115.9 (3)	O31—C37—C31	117.9 (3)
C23—C22—C21	120.0 (4)	O32—C37—C31	115.9 (4)
C22—C23—C24	119.6 (4)		
			
C6—N1—C2—C3	−51.8 (4)	C22—C21—C26—C25	−0.1 (5)
N1—C2—C3—N4	56.7 (4)	N4—C21—C26—C25	178.0 (3)
C2—C3—N4—C21	166.2 (3)	C24—C25—C26—C21	1.3 (6)
C2—C3—N4—C5	−62.2 (3)	C23—C22—O22—C27	−2.9 (5)
C21—N4—C5—C6	−166.8 (3)	C21—C22—O22—C27	177.8 (3)
C3—N4—C5—C6	62.2 (4)	C36—C31—C32—C33	0.7 (5)
C2—N1—C6—C5	53.2 (4)	C37—C31—C32—C33	−178.1 (4)
N4—C5—C6—N1	−58.0 (4)	C36—C31—C32—Cl32	−178.7 (3)
C3—N4—C21—C26	112.9 (3)	C37—C31—C32—Cl32	2.4 (4)
C5—N4—C21—C26	−15.3 (4)	C31—C32—C33—C34	−0.7 (6)
C3—N4—C21—C22	−68.9 (4)	Cl32—C32—C33—C34	178.7 (3)
C5—N4—C21—C22	162.8 (3)	C32—C33—C34—C35	0.2 (7)
C26—C21—C22—O22	177.9 (3)	C33—C34—C35—C36	0.3 (7)
N4—C21—C22—O22	−0.3 (4)	C34—C35—C36—C31	−0.3 (6)
C26—C21—C22—C23	−1.4 (5)	C32—C31—C36—C35	−0.2 (5)
N4—C21—C22—C23	−179.6 (3)	C37—C31—C36—C35	178.6 (4)
O22—C22—C23—C24	−177.6 (4)	C36—C31—C37—O31	−101.0 (4)
C21—C22—C23—C24	1.7 (5)	C32—C31—C37—O31	77.8 (4)
C22—C23—C24—C25	−0.5 (6)	C36—C31—C37—O32	79.5 (4)
C23—C24—C25—C26	−0.9 (6)	C32—C31—C37—O32	−101.7 (4)

### 4-(2-Methoxyphenyl)piperazin-1-ium 2-chlorobenzoate (V) Hydrogen-bond geometry (Å, º)

**Table:**

*D*—H···*A*	*D*—H	H···*A*	*D*···*A*	*D*—H···*A*
N1—H11···O31	0.97 (4)	1.74 (3)	2.682 (4)	162 (3)
N1—H12···O32^i^	0.92 (4)	1.79 (4)	2.700 (5)	170 (4)
C5—H5*B*···*Cg*1^ii^	0.97	2.87	3.554 (4)	128
C34—H34···*Cg*2^iii^	0.93	2.93	3.658 (7)	136

Symmetry codes: (i) −*x*+1, −*y*+1, −*z*+1; (ii) *x*+1, *y*, *z*; (iii) *x*−1, *y*, *z*+1.

### 4-(2-Methoxyphenyl)piperazin-1-ium 2-bromobenzoate (VI) Crystal data

**Table:**

C_11_H_17_N_2_O^+^·C_7_H_4_BrO_2_^−^	*D*_x_ = 1.451 Mg m^−^^3^
*M**_r_* = 393.28	Mo *K*α radiation, λ = 0.71073 Å
Orthorhombic, *P*2_1_2_1_2_1_	Cell parameters from 3863 reflections
*a* = 6.9824 (2) Å	θ = 5.6–89.3°
*b* = 13.2292 (4) Å	µ = 2.30 mm^−^^1^
*c* = 19.4903 (7) Å	*T* = 293 K
*V* = 1800.35 (10) Å^3^	Block, orange
*Z* = 4	0.50 × 0.50 × 0.48 mm
*F*(000) = 808	

### 4-(2-Methoxyphenyl)piperazin-1-ium 2-bromobenzoate (VI) Data collection

**Table:**

Oxford Diffraction Xcalibur with Sapphire CCD diffractometer	3895 independent reflections
Radiation source: Enhance (Mo) X-ray Source	2640 reflections with *I* > 2σ(*I*)
Graphite monochromator	*R*_int_ = 0.033
ω scans	θ_max_ = 27.7°, θ_min_ = 2.6°
Absorption correction: multi-scan (CrysAlis RED; Oxford Diffraction, 2009)	*h* = −9→9
*T*_min_ = 0.297, *T*_max_ = 0.331	*k* = −16→17
13089 measured reflections	*l* = −24→23

### 4-(2-Methoxyphenyl)piperazin-1-ium 2-bromobenzoate (VI) Refinement

**Table:**

Refinement on *F*^2^	Hydrogen site location: mixed
Least-squares matrix: full	H atoms treated by a mixture of independent and constrained refinement
*R*[*F*^2^ > 2σ(*F*^2^)] = 0.035	*w* = 1/[σ^2^(*F*_o_^2^) + (0.0418*P*)^2^] where *P* = (*F*_o_^2^ + 2*F*_c_^2^)/3
*wR*(*F*^2^) = 0.077	(Δ/σ)_max_ = 0.001
*S* = 0.94	Δρ_max_ = 0.28 e Å^−^^3^
3895 reflections	Δρ_min_ = −0.53 e Å^−^^3^
224 parameters	Absolute structure: Flack *x* determined using 919 quotients [(*I*^+^)-(*I*^-^)]/[(*I*^+^)+(*I*^-^)] (Parsons *et al.*, 2013)
0 restraints	Absolute structure parameter: 0.004 (5)
Primary atom site location: difference Fourier map	

### 4-(2-Methoxyphenyl)piperazin-1-ium 2-bromobenzoate (VI) Special details

**Table:**

Experimental. Empirical absorption correction using spherical harmonics, implemented in SCALE3 ABSPACK scaling algorithm.
Geometry. All esds (except the esd in the dihedral angle between two l.s. planes) are estimated using the full covariance matrix. The cell esds are taken into account individually in the estimation of esds in distances, angles and torsion angles; correlations between esds in cell parameters are only used when they are defined by crystal symmetry. An approximate (isotropic) treatment of cell esds is used for estimating esds involving l.s. planes.

### 4-(2-Methoxyphenyl)piperazin-1-ium 2-bromobenzoate (VI) Fractional atomic coordinates and isotropic or equivalent isotropic displacement parameters (Å^2^)

**Table:**

	*x*	*y*	*z*	*U*_iso_*/*U*_eq_	
N1	0.2366 (5)	0.3584 (2)	0.50360 (15)	0.0385 (7)	
H11	0.156 (5)	0.338 (3)	0.5251 (19)	0.046*	
H12	0.271 (5)	0.307 (2)	0.4774 (17)	0.046*	
C2	0.4021 (5)	0.3893 (3)	0.54630 (18)	0.0422 (9)	
H2A	0.4540	0.3309	0.5699	0.051*	
H2B	0.3611	0.4380	0.5805	0.051*	
C3	0.5535 (5)	0.4355 (2)	0.50113 (17)	0.0351 (8)	
H3A	0.6611	0.4572	0.5290	0.042*	
H3B	0.5993	0.3855	0.4686	0.042*	
N4	0.4745 (4)	0.52259 (19)	0.46388 (14)	0.0323 (7)	
C5	0.3155 (5)	0.4908 (2)	0.42030 (18)	0.0356 (9)	
H5A	0.3607	0.4421	0.3868	0.043*	
H5B	0.2651	0.5488	0.3957	0.043*	
C6	0.1594 (5)	0.4441 (3)	0.4630 (2)	0.0446 (10)	
H6A	0.1064	0.4946	0.4938	0.054*	
H6B	0.0574	0.4203	0.4334	0.054*	
C21	0.6096 (5)	0.5876 (2)	0.43291 (17)	0.0318 (8)	
C22	0.7393 (5)	0.6421 (2)	0.47504 (16)	0.0353 (7)	
C23	0.8583 (5)	0.7139 (3)	0.4459 (2)	0.0493 (10)	
H23	0.9422	0.7502	0.4735	0.059*	
C24	0.8539 (6)	0.7323 (3)	0.3762 (2)	0.0574 (11)	
H24	0.9346	0.7808	0.3573	0.069*	
C25	0.7323 (5)	0.6800 (3)	0.33479 (18)	0.0482 (9)	
H25	0.7286	0.6931	0.2879	0.058*	
C26	0.6140 (5)	0.6070 (3)	0.36316 (18)	0.0392 (9)	
H26	0.5348	0.5698	0.3343	0.047*	
O22	0.7279 (4)	0.62323 (15)	0.54385 (11)	0.0412 (6)	
C27	0.8382 (6)	0.6854 (3)	0.58800 (19)	0.0531 (11)	
H27A	0.9718	0.6758	0.5784	0.080*	
H27B	0.8126	0.6675	0.6348	0.080*	
H27C	0.8048	0.7549	0.5806	0.080*	
C31	0.0331 (5)	0.3548 (2)	0.67134 (16)	0.0357 (8)	
C32	0.1964 (5)	0.3120 (3)	0.69802 (18)	0.0444 (9)	
Br32	0.23614 (7)	0.16981 (3)	0.68887 (3)	0.0746 (2)	
C33	0.3352 (6)	0.3685 (4)	0.7308 (2)	0.0629 (13)	
H33	0.4435	0.3377	0.7491	0.076*	
C34	0.3097 (7)	0.4713 (4)	0.7360 (2)	0.0712 (15)	
H34	0.4033	0.5110	0.7566	0.085*	
C35	0.1467 (8)	0.5153 (4)	0.7107 (2)	0.0685 (14)	
H35	0.1279	0.5845	0.7158	0.082*	
C36	0.0120 (6)	0.4583 (3)	0.6783 (2)	0.0527 (10)	
H36	−0.0964	0.4895	0.6605	0.063*	
C37	−0.1145 (5)	0.2952 (3)	0.6315 (2)	0.0397 (9)	
O31	−0.0853 (3)	0.29029 (17)	0.56785 (12)	0.0409 (6)	
O32	−0.2506 (4)	0.2563 (2)	0.66127 (13)	0.0792 (9)	

### 4-(2-Methoxyphenyl)piperazin-1-ium 2-bromobenzoate (VI) Atomic displacement parameters (Å^2^)

**Table:**

	*U*^11^	*U*^22^	*U*^33^	*U*^12^	*U*^13^	*U*^23^
N1	0.0415 (17)	0.0396 (15)	0.0344 (17)	−0.0058 (17)	0.0099 (16)	−0.0041 (12)
C2	0.048 (2)	0.044 (2)	0.035 (2)	0.0010 (17)	0.0020 (18)	0.0005 (17)
C3	0.0376 (18)	0.0328 (18)	0.035 (2)	−0.0018 (16)	−0.0022 (16)	−0.0011 (16)
N4	0.0320 (15)	0.0302 (15)	0.0348 (18)	−0.0001 (13)	−0.0053 (13)	0.0004 (13)
C5	0.037 (2)	0.0373 (19)	0.033 (2)	−0.0035 (15)	−0.0070 (15)	0.0017 (15)
C6	0.0376 (19)	0.045 (2)	0.051 (2)	−0.0019 (17)	−0.0041 (18)	0.0004 (19)
C21	0.0355 (18)	0.0291 (16)	0.031 (2)	0.0035 (15)	0.0023 (16)	−0.0034 (15)
C22	0.0340 (17)	0.0379 (17)	0.034 (2)	−0.0008 (18)	−0.0025 (18)	−0.0017 (14)
C23	0.041 (2)	0.057 (2)	0.050 (3)	−0.0128 (19)	−0.0041 (18)	0.001 (2)
C24	0.051 (2)	0.069 (3)	0.052 (3)	−0.017 (2)	0.006 (2)	0.014 (2)
C25	0.045 (2)	0.070 (2)	0.0304 (19)	−0.005 (2)	0.0032 (18)	0.0057 (17)
C26	0.039 (2)	0.045 (2)	0.034 (2)	−0.0004 (17)	−0.0016 (17)	−0.0041 (17)
O22	0.0488 (14)	0.0450 (12)	0.0297 (13)	−0.0095 (13)	−0.0065 (13)	−0.0008 (10)
C27	0.059 (2)	0.061 (3)	0.039 (2)	−0.008 (2)	−0.0174 (19)	−0.0053 (19)
C31	0.0362 (19)	0.046 (2)	0.025 (2)	−0.0065 (16)	0.0051 (15)	−0.0024 (15)
C32	0.0384 (19)	0.065 (2)	0.030 (2)	−0.0035 (17)	0.0008 (16)	0.0064 (19)
Br32	0.0640 (3)	0.0691 (3)	0.0907 (4)	0.0123 (3)	−0.0067 (3)	0.0203 (3)
C33	0.045 (2)	0.107 (4)	0.037 (2)	−0.014 (3)	−0.0080 (19)	0.012 (2)
C34	0.070 (3)	0.103 (4)	0.040 (3)	−0.045 (3)	0.006 (2)	−0.022 (3)
C35	0.087 (3)	0.060 (3)	0.058 (3)	−0.023 (3)	0.016 (3)	−0.015 (2)
C36	0.058 (2)	0.051 (2)	0.049 (3)	−0.004 (2)	0.002 (2)	−0.006 (2)
C37	0.0325 (19)	0.048 (2)	0.039 (2)	0.0034 (17)	−0.0004 (17)	−0.0047 (18)
O31	0.0475 (14)	0.0471 (14)	0.0283 (14)	−0.0077 (12)	0.0019 (12)	−0.0046 (12)
O32	0.0561 (18)	0.131 (3)	0.0509 (17)	−0.044 (2)	0.0165 (16)	−0.0198 (16)

### 4-(2-Methoxyphenyl)piperazin-1-ium 2-bromobenzoate (VI) Geometric parameters (Å, º)

**Table:**

N1—C2	1.481 (5)	C24—C25	1.360 (5)
N1—C6	1.484 (4)	C24—H24	0.9300
N1—H11	0.75 (4)	C25—C26	1.386 (5)
N1—H12	0.89 (3)	C25—H25	0.9300
C2—C3	1.506 (5)	C26—H26	0.9300
C2—H2A	0.9700	O22—C27	1.417 (4)
C2—H2B	0.9700	C27—H27A	0.9600
C3—N4	1.469 (4)	C27—H27B	0.9600
C3—H3A	0.9700	C27—H27C	0.9600
C3—H3B	0.9700	C31—C32	1.375 (5)
N4—C21	1.412 (4)	C31—C36	1.383 (4)
N4—C5	1.460 (4)	C31—C37	1.512 (5)
C5—C6	1.505 (5)	C32—C33	1.381 (5)
C5—H5A	0.9700	C32—Br32	1.910 (4)
C5—H5B	0.9700	C33—C34	1.375 (7)
C6—H6A	0.9700	C33—H33	0.9300
C6—H6B	0.9700	C34—C35	1.370 (7)
C21—C26	1.384 (5)	C34—H34	0.9300
C21—C22	1.419 (4)	C35—C36	1.361 (6)
C22—O22	1.366 (4)	C35—H35	0.9300
C22—C23	1.385 (5)	C36—H36	0.9300
C23—C24	1.380 (5)	C37—O32	1.226 (4)
C23—H23	0.9300	C37—O31	1.260 (4)
			
C2—N1—C6	111.8 (3)	C24—C23—H23	119.6
C2—N1—H11	112 (3)	C22—C23—H23	119.6
C6—N1—H11	107 (3)	C25—C24—C23	120.5 (4)
C2—N1—H12	109 (2)	C25—C24—H24	119.7
C6—N1—H12	112 (2)	C23—C24—H24	119.7
H11—N1—H12	104 (3)	C24—C25—C26	119.3 (3)
N1—C2—C3	109.3 (3)	C24—C25—H25	120.3
N1—C2—H2A	109.8	C26—C25—H25	120.3
C3—C2—H2A	109.8	C21—C26—C25	122.3 (3)
N1—C2—H2B	109.8	C21—C26—H26	118.8
C3—C2—H2B	109.8	C25—C26—H26	118.8
H2A—C2—H2B	108.3	C22—O22—C27	117.3 (3)
N4—C3—C2	110.1 (3)	O22—C27—H27A	109.5
N4—C3—H3A	109.6	O22—C27—H27B	109.5
C2—C3—H3A	109.6	H27A—C27—H27B	109.5
N4—C3—H3B	109.6	O22—C27—H27C	109.5
C2—C3—H3B	109.6	H27A—C27—H27C	109.5
H3A—C3—H3B	108.2	H27B—C27—H27C	109.5
C21—N4—C5	115.8 (3)	C32—C31—C36	117.3 (3)
C21—N4—C3	116.0 (3)	C32—C31—C37	123.0 (3)
C5—N4—C3	110.3 (3)	C36—C31—C37	119.6 (3)
N4—C5—C6	110.3 (3)	C31—C32—C33	122.3 (4)
N4—C5—H5A	109.6	C31—C32—Br32	119.4 (3)
C6—C5—H5A	109.6	C33—C32—Br32	118.3 (3)
N4—C5—H5B	109.6	C34—C33—C32	118.6 (4)
C6—C5—H5B	109.6	C34—C33—H33	120.7
H5A—C5—H5B	108.1	C32—C33—H33	120.7
N1—C6—C5	110.2 (3)	C35—C34—C33	120.1 (4)
N1—C6—H6A	109.6	C35—C34—H34	119.9
C5—C6—H6A	109.6	C33—C34—H34	119.9
N1—C6—H6B	109.6	C36—C35—C34	120.3 (4)
C5—C6—H6B	109.6	C36—C35—H35	119.8
H6A—C6—H6B	108.1	C34—C35—H35	119.8
C26—C21—N4	123.2 (3)	C35—C36—C31	121.4 (4)
C26—C21—C22	117.4 (3)	C35—C36—H36	119.3
N4—C21—C22	119.2 (3)	C31—C36—H36	119.3
O22—C22—C23	124.3 (3)	O32—C37—O31	124.8 (3)
O22—C22—C21	116.0 (3)	O32—C37—C31	120.3 (3)
C23—C22—C21	119.6 (3)	O31—C37—C31	114.9 (3)
C24—C23—C22	120.7 (3)		
			
C6—N1—C2—C3	−56.0 (4)	N4—C21—C26—C25	172.1 (3)
N1—C2—C3—N4	58.1 (4)	C22—C21—C26—C25	−3.2 (5)
C2—C3—N4—C21	165.1 (3)	C24—C25—C26—C21	2.4 (5)
C2—C3—N4—C5	−60.7 (3)	C23—C22—O22—C27	3.5 (5)
C21—N4—C5—C6	−166.1 (3)	C21—C22—O22—C27	−172.2 (3)
C3—N4—C5—C6	59.7 (4)	C36—C31—C32—C33	0.2 (5)
C2—N1—C6—C5	55.3 (4)	C37—C31—C32—C33	176.2 (3)
N4—C5—C6—N1	−56.5 (4)	C36—C31—C32—Br32	−179.1 (3)
C5—N4—C21—C26	−11.1 (4)	C37—C31—C32—Br32	−3.1 (4)
C3—N4—C21—C26	120.6 (3)	C31—C32—C33—C34	−1.0 (6)
C5—N4—C21—C22	164.2 (3)	Br32—C32—C33—C34	178.3 (3)
C3—N4—C21—C22	−64.2 (4)	C32—C33—C34—C35	2.0 (6)
C26—C21—C22—O22	178.3 (3)	C33—C34—C35—C36	−2.3 (7)
N4—C21—C22—O22	2.8 (4)	C34—C35—C36—C31	1.5 (6)
C26—C21—C22—C23	2.4 (5)	C32—C31—C36—C35	−0.5 (6)
N4—C21—C22—C23	−173.2 (3)	C37—C31—C36—C35	−176.6 (4)
O22—C22—C23—C24	−176.4 (4)	C32—C31—C37—O32	91.3 (4)
C21—C22—C23—C24	−0.8 (5)	C36—C31—C37—O32	−92.8 (5)
C22—C23—C24—C25	0.0 (6)	C32—C31—C37—O31	−89.1 (4)
C23—C24—C25—C26	−0.7 (6)	C36—C31—C37—O31	86.8 (4)

### 4-(2-Methoxyphenyl)piperazin-1-ium 2-bromobenzoate (VI) Hydrogen-bond geometry (Å, º)

**Table:**

*D*—H···*A*	*D*—H	H···*A*	*D*···*A*	*D*—H···*A*
N1—H11···O31	0.75 (4)	1.98 (4)	2.726 (4)	170 (4)
N1—H12···O31^i^	0.88 (3)	1.86 (3)	2.712 (4)	163 (3)
C25—H25···O32^ii^	0.93	2.56	3.488 (4)	173
C26—H26···*Cg*1^ii^	0.93	2.93	3.697 (4)	141

Symmetry codes: (i) *x*+1/2, −*y*+1/2, −*z*+1; (ii) −*x*+1/2, −*y*+1, *z*−1/2.

### 4-(2-Methoxyphenyl)piperazin-1-ium 2-iodobenzoate (VII) Crystal data

**Table:**

C_11_H_17_N_2_O^+^·C_7_H_4_IO_2_^−^	*D*_x_ = 1.595 Mg m^−^^3^
*M**_r_* = 440.27	Mo *K*α radiation, λ = 0.71073 Å
Orthorhombic, *P*2_1_2_1_2_1_	Cell parameters from 3735 reflections
*a* = 7.0101 (4) Å	θ = 2.6–27.8°
*b* = 13.3796 (6) Å	µ = 1.76 mm^−^^1^
*c* = 19.5524 (6) Å	*T* = 293 K
*V* = 1833.87 (14) Å^3^	Block, orange
*Z* = 4	0.50 × 0.50 × 0.48 mm
*F*(000) = 880	

### 4-(2-Methoxyphenyl)piperazin-1-ium 2-iodobenzoate (VII) Data collection

**Table:**

Oxford Diffraction Xcalibur with Sapphire CCD diffractometer	3735 independent reflections
Radiation source: Enhance (Mo) X-ray Source	3036 reflections with *I* > 2σ(*I*)
Graphite monochromator	*R*_int_ = 0.019
ω scans	θ_max_ = 27.8°, θ_min_ = 2.6°
Absorption correction: multi-scan (CrysAlis RED; Oxford Diffraction, 2009)	*h* = −5→9
*T*_min_ = 0.373, *T*_max_ = 0.431	*k* = −17→16
7500 measured reflections	*l* = −25→25

### 4-(2-Methoxyphenyl)piperazin-1-ium 2-iodobenzoate (VII) Refinement

**Table:**

Refinement on *F*^2^	Hydrogen site location: inferred from neighbouring sites
Least-squares matrix: full	H-atom parameters constrained
*R*[*F*^2^ > 2σ(*F*^2^)] = 0.032	*w* = 1/[σ^2^(*F*_o_^2^) + (0.0306*P*)^2^ + 0.7308*P*] where *P* = (*F*_o_^2^ + 2*F*_c_^2^)/3
*wR*(*F*^2^) = 0.071	(Δ/σ)_max_ < 0.001
*S* = 1.05	Δρ_max_ = 0.46 e Å^−^^3^
3735 reflections	Δρ_min_ = −0.65 e Å^−^^3^
237 parameters	Absolute structure: Flack *x* determined using 1045 quotients [(*I*^+^)-(*I*^-^)]/[(*I*^+^)+(*I*^-^)] (Parsons *et al.*, 2013)
17 restraints	Absolute structure parameter: 0.004 (10)
Primary atom site location: difference Fourier map	

### 4-(2-Methoxyphenyl)piperazin-1-ium 2-iodobenzoate (VII) Special details

**Table:**

Experimental. Empirical absorption correction using spherical harmonics, implemented in SCALE3 ABSPACK scaling algorithm.
Geometry. All esds (except the esd in the dihedral angle between two l.s. planes) are estimated using the full covariance matrix. The cell esds are taken into account individually in the estimation of esds in distances, angles and torsion angles; correlations between esds in cell parameters are only used when they are defined by crystal symmetry. An approximate (isotropic) treatment of cell esds is used for estimating esds involving l.s. planes.

### 4-(2-Methoxyphenyl)piperazin-1-ium 2-iodobenzoate (VII) Fractional atomic coordinates and isotropic or equivalent isotropic displacement parameters (Å^2^)

**Table:**

	*x*	*y*	*z*	*U*_iso_*/*U*_eq_	Occ. (<1)
N1	0.7532 (6)	0.6404 (2)	0.49690 (15)	0.0402 (7)	
H11	0.8436	0.6650	0.4697	0.048*	
H12	0.7154	0.6890	0.5249	0.048*	
C2	0.5901 (6)	0.6069 (4)	0.4550 (2)	0.0423 (11)	
H2A	0.6330	0.5584	0.4215	0.051*	
H2B	0.5361	0.6634	0.4307	0.051*	
C3	0.4399 (6)	0.5604 (3)	0.5005 (2)	0.0387 (10)	
H3A	0.3923	0.6100	0.5324	0.046*	
H3B	0.3338	0.5374	0.4728	0.046*	
N4	0.5212 (5)	0.4761 (3)	0.53831 (17)	0.0339 (8)	
C5	0.6810 (6)	0.5097 (3)	0.5809 (2)	0.0396 (10)	
H5A	0.7349	0.4530	0.6051	0.048*	
H5B	0.6356	0.5574	0.6145	0.048*	
C6	0.8318 (7)	0.5575 (4)	0.5374 (3)	0.0487 (12)	
H6A	0.9335	0.5823	0.5664	0.058*	
H6B	0.8857	0.5078	0.5068	0.058*	
C21	0.3893 (5)	0.4115 (3)	0.5700 (2)	0.0338 (9)	
C22	0.2609 (8)	0.3565 (3)	0.52887 (19)	0.0372 (9)	
C23	0.1420 (7)	0.2860 (4)	0.5579 (3)	0.0517 (12)	
H23	0.0584	0.2496	0.5307	0.062*	
C24	0.1478 (8)	0.2697 (4)	0.6279 (3)	0.0583 (14)	
H24	0.0678	0.2219	0.6472	0.070*	
C25	0.2675 (8)	0.3219 (4)	0.6684 (2)	0.0527 (12)	
H25	0.2700	0.3102	0.7153	0.063*	
C26	0.3866 (6)	0.3931 (3)	0.6399 (2)	0.0418 (11)	
H26	0.4670	0.4296	0.6683	0.050*	
O22	0.2722 (6)	0.3732 (2)	0.45961 (14)	0.0432 (7)	
C27	0.1621 (8)	0.3095 (4)	0.4164 (3)	0.0574 (14)	
H27A	0.0289	0.3190	0.4257	0.086*	
H27B	0.1959	0.2411	0.4249	0.086*	
H27C	0.1874	0.3255	0.3694	0.086*	
C31	0.9641 (6)	0.6382 (4)	0.3324 (2)	0.0387 (10)	
C32	0.8007 (6)	0.6775 (4)	0.3027 (2)	0.0421 (11)	
I32	0.74794 (6)	0.83201 (2)	0.30799 (2)	0.06407 (13)	
C33	0.6680 (8)	0.6168 (5)	0.2703 (3)	0.0596 (15)	
H33	0.5591	0.6443	0.2506	0.072*	
C34	0.7001 (8)	0.5157 (5)	0.2677 (3)	0.0689 (19)	
H34	0.6110	0.4741	0.2470	0.083*	
C35	0.8613 (10)	0.4758 (5)	0.2953 (3)	0.0660 (16)	
H35	0.8834	0.4075	0.2922	0.079*	
C36	0.9913 (7)	0.5360 (4)	0.3277 (3)	0.0536 (13)	
H36	1.0999	0.5075	0.3469	0.064*	
C37	1.1071 (6)	0.7004 (3)	0.3715 (2)	0.0389 (10)	0.54 (9)
O31	1.072 (4)	0.699 (3)	0.4352 (5)	0.033 (3)	0.54 (9)
O32	1.233 (4)	0.750 (3)	0.3440 (12)	0.065 (5)	0.54 (9)
C38	1.1071 (6)	0.7004 (3)	0.3715 (2)	0.0389 (10)	0.46 (9)
O33	1.070 (4)	0.726 (3)	0.4325 (7)	0.030 (4)	0.46 (9)
O34	1.258 (3)	0.716 (4)	0.3417 (13)	0.065 (6)	0.46 (9)

### 4-(2-Methoxyphenyl)piperazin-1-ium 2-iodobenzoate (VII) Atomic displacement parameters (Å^2^)

**Table:**

	*U*^11^	*U*^22^	*U*^33^	*U*^12^	*U*^13^	*U*^23^
N1	0.0418 (17)	0.0403 (16)	0.0384 (16)	−0.005 (3)	0.013 (2)	−0.0056 (13)
C2	0.045 (3)	0.047 (3)	0.035 (2)	−0.002 (2)	0.002 (2)	−0.002 (2)
C3	0.042 (2)	0.036 (2)	0.038 (2)	−0.003 (2)	−0.005 (2)	0.001 (2)
N4	0.0337 (19)	0.0347 (19)	0.0334 (18)	−0.0026 (16)	−0.0045 (15)	−0.0008 (16)
C5	0.037 (2)	0.044 (2)	0.038 (2)	−0.0003 (19)	−0.0061 (19)	−0.004 (2)
C6	0.040 (2)	0.053 (3)	0.054 (3)	−0.001 (2)	−0.005 (2)	−0.006 (3)
C21	0.031 (2)	0.034 (2)	0.036 (2)	0.0025 (18)	0.0003 (19)	−0.0023 (19)
C22	0.037 (2)	0.038 (2)	0.0362 (19)	0.002 (3)	0.000 (3)	0.0002 (15)
C23	0.044 (3)	0.057 (3)	0.054 (3)	−0.010 (2)	−0.004 (2)	0.004 (3)
C24	0.052 (3)	0.068 (4)	0.055 (3)	−0.016 (3)	0.008 (3)	0.017 (3)
C25	0.050 (3)	0.068 (3)	0.041 (2)	−0.003 (4)	0.006 (3)	0.010 (2)
C26	0.042 (2)	0.050 (3)	0.033 (2)	0.000 (2)	0.002 (2)	−0.004 (2)
O22	0.0477 (19)	0.0469 (15)	0.0350 (13)	−0.0054 (18)	−0.0046 (17)	0.0000 (12)
C27	0.063 (3)	0.067 (3)	0.043 (3)	−0.015 (3)	−0.014 (3)	−0.007 (3)
C31	0.035 (2)	0.051 (3)	0.030 (2)	−0.003 (2)	0.0036 (18)	−0.004 (2)
C32	0.041 (3)	0.055 (3)	0.030 (2)	−0.0081 (19)	0.0015 (18)	0.004 (2)
I32	0.05832 (19)	0.0631 (2)	0.0708 (2)	0.0097 (3)	−0.0032 (3)	0.01731 (17)
C33	0.049 (3)	0.090 (4)	0.041 (3)	−0.014 (3)	−0.010 (2)	0.003 (3)
C34	0.073 (5)	0.087 (5)	0.047 (3)	−0.037 (3)	0.004 (3)	−0.017 (3)
C35	0.078 (4)	0.058 (3)	0.062 (4)	−0.014 (3)	0.016 (3)	−0.020 (3)
C36	0.050 (3)	0.054 (3)	0.056 (3)	−0.001 (2)	0.001 (2)	−0.009 (3)
C37	0.032 (2)	0.046 (3)	0.039 (3)	−0.002 (2)	0.000 (2)	−0.003 (2)
O31	0.051 (5)	0.015 (9)	0.034 (4)	−0.012 (7)	0.003 (4)	−0.005 (3)
O32	0.066 (8)	0.072 (11)	0.057 (6)	−0.031 (7)	0.019 (7)	−0.022 (6)
C38	0.032 (2)	0.046 (3)	0.039 (3)	−0.002 (2)	0.000 (2)	−0.003 (2)
O33	0.042 (5)	0.010 (9)	0.038 (5)	−0.009 (7)	0.002 (4)	−0.004 (4)
O34	0.039 (6)	0.105 (16)	0.053 (6)	−0.020 (9)	0.009 (6)	−0.037 (9)

### 4-(2-Methoxyphenyl)piperazin-1-ium 2-iodobenzoate (VII) Geometric parameters (Å, º)

**Table:**

N1—C6	1.470 (6)	C24—C25	1.350 (7)
N1—C2	1.476 (6)	C24—H24	0.9300
N1—H11	0.8900	C25—C26	1.383 (6)
N1—H12	0.8900	C25—H25	0.9300
C2—C3	1.512 (6)	C26—H26	0.9300
C2—H2A	0.9700	O22—C27	1.428 (6)
C2—H2B	0.9700	C27—H27A	0.9600
C3—N4	1.464 (5)	C27—H27B	0.9600
C3—H3A	0.9700	C27—H27C	0.9600
C3—H3B	0.9700	C31—C36	1.384 (7)
N4—C21	1.410 (5)	C31—C32	1.387 (6)
N4—C5	1.466 (6)	C31—C37	1.511 (6)
C5—C6	1.500 (7)	C32—C33	1.388 (7)
C5—H5A	0.9700	C32—I32	2.103 (5)
C5—H5B	0.9700	C33—C34	1.372 (9)
C6—H6A	0.9700	C33—H33	0.9300
C6—H6B	0.9700	C34—C35	1.361 (8)
C21—C26	1.389 (6)	C34—H34	0.9300
C21—C22	1.413 (6)	C35—C36	1.370 (8)
C22—O22	1.375 (5)	C35—H35	0.9300
C22—C23	1.382 (6)	C36—H36	0.9300
C23—C24	1.385 (7)	C37—O32	1.226 (9)
C23—H23	0.9300	C37—O31	1.269 (8)
			
C6—N1—C2	111.1 (3)	C22—C23—H23	120.1
C6—N1—H11	109.4	C24—C23—H23	120.1
C2—N1—H11	109.4	C25—C24—C23	121.1 (5)
C6—N1—H12	109.4	C25—C24—H24	119.5
C2—N1—H12	109.4	C23—C24—H24	119.5
H11—N1—H12	108.0	C24—C25—C26	119.7 (4)
N1—C2—C3	109.8 (3)	C24—C25—H25	120.2
N1—C2—H2A	109.7	C26—C25—H25	120.2
C3—C2—H2A	109.7	C25—C26—C21	121.8 (4)
N1—C2—H2B	109.7	C25—C26—H26	119.1
C3—C2—H2B	109.7	C21—C26—H26	119.1
H2A—C2—H2B	108.2	C22—O22—C27	117.1 (4)
N4—C3—C2	110.1 (4)	O22—C27—H27A	109.5
N4—C3—H3A	109.6	O22—C27—H27B	109.5
C2—C3—H3A	109.6	H27A—C27—H27B	109.5
N4—C3—H3B	109.6	O22—C27—H27C	109.5
C2—C3—H3B	109.6	H27A—C27—H27C	109.5
H3A—C3—H3B	108.2	H27B—C27—H27C	109.5
C21—N4—C3	116.0 (3)	C36—C31—C32	117.4 (4)
C21—N4—C5	116.1 (3)	C36—C31—C37	119.1 (4)
C3—N4—C5	110.4 (3)	C32—C31—C37	123.4 (4)
N4—C5—C6	110.3 (4)	C31—C32—C33	121.5 (5)
N4—C5—H5A	109.6	C31—C32—I32	119.8 (3)
C6—C5—H5A	109.6	C33—C32—I32	118.6 (4)
N4—C5—H5B	109.6	C34—C33—C32	118.9 (5)
C6—C5—H5B	109.6	C34—C33—H33	120.6
H5A—C5—H5B	108.1	C32—C33—H33	120.6
N1—C6—C5	111.3 (4)	C35—C34—C33	120.6 (5)
N1—C6—H6A	109.4	C35—C34—H34	119.7
C5—C6—H6A	109.4	C33—C34—H34	119.7
N1—C6—H6B	109.4	C34—C35—C36	120.3 (6)
C5—C6—H6B	109.4	C34—C35—H35	119.8
H6A—C6—H6B	108.0	C36—C35—H35	119.8
C26—C21—N4	123.4 (4)	C35—C36—C31	121.3 (5)
C26—C21—C22	117.3 (4)	C35—C36—H36	119.4
N4—C21—C22	119.1 (4)	C31—C36—H36	119.4
O22—C22—C23	123.4 (4)	O32—C37—O31	125.5 (12)
O22—C22—C21	116.0 (4)	O32—C37—C31	123.5 (13)
C23—C22—C21	120.4 (4)	O31—C37—C31	111.0 (13)
C22—C23—C24	119.7 (5)		
			
C6—N1—C2—C3	56.3 (5)	C24—C25—C26—C21	−1.1 (8)
N1—C2—C3—N4	−58.4 (5)	N4—C21—C26—C25	−173.6 (4)
C2—C3—N4—C21	−165.5 (4)	C22—C21—C26—C25	1.8 (7)
C2—C3—N4—C5	59.7 (5)	C23—C22—O22—C27	−3.7 (7)
C21—N4—C5—C6	167.0 (4)	C21—C22—O22—C27	172.1 (4)
C3—N4—C5—C6	−58.3 (5)	C36—C31—C32—C33	0.8 (6)
C2—N1—C6—C5	−55.6 (5)	C37—C31—C32—C33	−176.6 (4)
N4—C5—C6—N1	56.2 (5)	C36—C31—C32—I32	179.7 (3)
C3—N4—C21—C26	−121.0 (4)	C37—C31—C32—I32	2.3 (5)
C5—N4—C21—C26	11.1 (6)	C31—C32—C33—C34	0.0 (7)
C3—N4—C21—C22	63.7 (5)	I32—C32—C33—C34	−178.9 (4)
C5—N4—C21—C22	−164.2 (4)	C32—C33—C34—C35	−1.4 (8)
C26—C21—C22—O22	−177.4 (4)	C33—C34—C35—C36	1.8 (8)
N4—C21—C22—O22	−1.8 (6)	C34—C35—C36—C31	−0.9 (8)
C26—C21—C22—C23	−1.5 (6)	C32—C31—C36—C35	−0.4 (7)
N4—C21—C22—C23	174.1 (4)	C37—C31—C36—C35	177.1 (5)
O22—C22—C23—C24	176.1 (5)	C36—C31—C37—O32	101 (2)
C21—C22—C23—C24	0.5 (8)	C32—C31—C37—O32	−82 (2)
C22—C23—C24—C25	0.2 (9)	C36—C31—C37—O31	−82.8 (18)
C23—C24—C25—C26	0.1 (9)	C32—C31—C37—O31	94.6 (18)

### 4-(2-Methoxyphenyl)piperazin-1-ium 2-iodobenzoate (VII) Hydrogen-bond geometry (Å, º)

**Table:**

*D*—H···*A*	*D*—H	H···*A*	*D*···*A*	*D*—H···*A*
N1—H11···O31	0.89	1.80	2.66 (3)	162
N1—H11···O33	0.89	1.93	2.80 (3)	165
N1—H12···O31^i^	0.89	1.97	2.83 (3)	162
N1—H12···O33^i^	0.89	1.74	2.60 (3)	161
C25—H25···O34^ii^	0.93	2.50	3.43 (3)	174
C26—H26···*Cg*1^ii^	0.93	2.93	3.716 (5)	143

Symmetry codes: (i) *x*−1/2, −*y*+3/2, −*z*+1; (ii) −*x*+3/2, −*y*+1, *z*+1/2.

### 4-(2-Methoxyphenyl)piperazin-1-ium 2-methylbenzoate (VIII) Crystal data

**Table:**

C_11_H_17_N_2_O^+^·C_8_H_7_O_2_^−^	*Z* = 2
*M**_r_* = 328.40	*F*(000) = 352
Triclinic, *P*1	*D*_x_ = 1.206 Mg m^−^^3^
*a* = 7.826 (1) Å	Mo *K*α radiation, λ = 0.71073 Å
*b* = 10.320 (2) Å	Cell parameters from 3838 reflections
*c* = 12.055 (3) Å	θ = 2.7–27.7°
α = 78.37 (2)°	µ = 0.08 mm^−^^1^
β = 78.27 (2)°	*T* = 296 K
γ = 73.83 (2)°	Block, colourless
*V* = 904.6 (3) Å^3^	0.48 × 0.48 × 0.40 mm

### 4-(2-Methoxyphenyl)piperazin-1-ium 2-methylbenzoate (VIII) Data collection

**Table:**

Oxford Diffraction Xcalibur with Sapphire CCD diffractometer	3838 independent reflections
Radiation source: Enhance (Mo) X-ray Source	2600 reflections with *I* > 2σ(*I*)
Graphite monochromator	*R*_int_ = 0.013
ω scans	θ_max_ = 27.7°, θ_min_ = 2.7°
Absorption correction: multi-scan (CrysAlis RED; Oxford Diffraction, 2009)	*h* = −5→10
*T*_min_ = 0.883, *T*_max_ = 0.968	*k* = −11→13
6091 measured reflections	*l* = −15→15

### 4-(2-Methoxyphenyl)piperazin-1-ium 2-methylbenzoate (VIII) Refinement

**Table:**

Refinement on *F*^2^	Hydrogen site location: mixed
Least-squares matrix: full	H atoms treated by a mixture of independent and constrained refinement
*R*[*F*^2^ > 2σ(*F*^2^)] = 0.042	*w* = 1/[σ^2^(*F*_o_^2^) + (0.0611*P*)^2^ + 0.0332*P*] where *P* = (*F*_o_^2^ + 2*F*_c_^2^)/3
*wR*(*F*^2^) = 0.119	(Δ/σ)_max_ < 0.001
*S* = 1.06	Δρ_max_ = 0.16 e Å^−^^3^
3838 reflections	Δρ_min_ = −0.16 e Å^−^^3^
226 parameters	Extinction correction: SHELXL, Fc^*^=kFc[1+0.001xFc^2^λ^3^/sin(2θ)]^-1/4^
0 restraints	Extinction coefficient: 0.057 (5)
Primary atom site location: difference Fourier map	

### 4-(2-Methoxyphenyl)piperazin-1-ium 2-methylbenzoate (VIII) Special details

**Table:**

Experimental. Empirical absorption correction using spherical harmonics, implemented in SCALE3 ABSPACK scaling algorithm.
Geometry. All esds (except the esd in the dihedral angle between two l.s. planes) are estimated using the full covariance matrix. The cell esds are taken into account individually in the estimation of esds in distances, angles and torsion angles; correlations between esds in cell parameters are only used when they are defined by crystal symmetry. An approximate (isotropic) treatment of cell esds is used for estimating esds involving l.s. planes.

### 4-(2-Methoxyphenyl)piperazin-1-ium 2-methylbenzoate (VIII) Fractional atomic coordinates and isotropic or equivalent isotropic displacement parameters (Å^2^)

**Table:**

	*x*	*y*	*z*	*U*_iso_*/*U*_eq_	
N1	0.42898 (16)	0.55387 (12)	0.64034 (10)	0.0429 (3)	
H11	0.3320 (19)	0.5684 (15)	0.5920 (12)	0.051*	
H12	0.5095 (19)	0.4646 (16)	0.6376 (12)	0.051*	
C2	0.33370 (18)	0.55987 (15)	0.75988 (11)	0.0449 (3)	
H2A	0.2790	0.4832	0.7859	0.054*	
H2B	0.2384	0.6433	0.7619	0.054*	
C3	0.46231 (18)	0.55582 (14)	0.83911 (11)	0.0448 (3)	
H3A	0.3967	0.5651	0.9157	0.054*	
H3B	0.5512	0.4688	0.8430	0.054*	
N4	0.55258 (15)	0.66676 (12)	0.79702 (9)	0.0445 (3)	
C5	0.6584 (2)	0.64945 (17)	0.68405 (12)	0.0534 (4)	
H5A	0.7448	0.5613	0.6881	0.064*	
H5B	0.7241	0.7198	0.6581	0.064*	
C6	0.5332 (2)	0.65903 (17)	0.60108 (12)	0.0542 (4)	
H6A	0.4510	0.7490	0.5945	0.065*	
H6B	0.6027	0.6466	0.5259	0.065*	
C21	0.63967 (18)	0.69298 (14)	0.87963 (11)	0.0434 (3)	
C22	0.53136 (18)	0.75748 (14)	0.97177 (12)	0.0453 (3)	
C23	0.6112 (2)	0.78557 (16)	1.05340 (13)	0.0562 (4)	
H23	0.5396	0.8262	1.1149	0.067*	
C24	0.7965 (2)	0.75375 (18)	1.04446 (14)	0.0628 (4)	
H24	0.8488	0.7740	1.0995	0.075*	
C25	0.9036 (2)	0.6926 (2)	0.95519 (15)	0.0693 (5)	
H25	1.0283	0.6715	0.9492	0.083*	
C26	0.8242 (2)	0.66219 (18)	0.87324 (14)	0.0599 (4)	
H26	0.8973	0.6202	0.8129	0.072*	
O22	0.34998 (13)	0.78566 (11)	0.97381 (9)	0.0602 (3)	
C27	0.2347 (2)	0.85827 (19)	1.06138 (16)	0.0734 (5)	
H27A	0.1119	0.8784	1.0492	0.110*	
H27B	0.2676	0.9419	1.0587	0.110*	
H27C	0.2472	0.8032	1.1350	0.110*	
C31	0.02692 (17)	0.80006 (14)	0.37927 (11)	0.0434 (3)	
C32	0.0231 (2)	0.93671 (15)	0.33658 (14)	0.0571 (4)	
C33	−0.1295 (3)	1.01830 (17)	0.29036 (17)	0.0752 (5)	
H33	−0.1323	1.1089	0.2594	0.090*	
C34	−0.2735 (2)	0.9696 (2)	0.28923 (16)	0.0774 (6)	
H34	−0.3733	1.0266	0.2588	0.093*	
C35	−0.2708 (2)	0.8359 (2)	0.33318 (14)	0.0660 (5)	
H35	−0.3690	0.8019	0.3331	0.079*	
C36	−0.12087 (18)	0.75165 (16)	0.37783 (12)	0.0523 (4)	
H36	−0.1195	0.6609	0.4074	0.063*	
C37	0.18760 (19)	0.70027 (14)	0.42484 (13)	0.0482 (4)	
O31	0.15924 (14)	0.62406 (13)	0.51830 (10)	0.0692 (3)	
O32	0.34037 (13)	0.69583 (10)	0.36497 (10)	0.0615 (3)	
C38	0.1751 (3)	0.99828 (18)	0.3413 (2)	0.0863 (6)	
H38A	0.1307	1.0954	0.3377	0.130*	
H38B	0.2688	0.9785	0.2775	0.130*	
H38C	0.2222	0.9600	0.4116	0.130*	

### 4-(2-Methoxyphenyl)piperazin-1-ium 2-methylbenzoate (VIII) Atomic displacement parameters (Å^2^)

**Table:**

	*U*^11^	*U*^22^	*U*^33^	*U*^12^	*U*^13^	*U*^23^
N1	0.0422 (6)	0.0442 (7)	0.0423 (7)	−0.0085 (5)	−0.0076 (5)	−0.0088 (5)
C2	0.0437 (7)	0.0458 (8)	0.0438 (8)	−0.0139 (6)	−0.0032 (6)	−0.0033 (6)
C3	0.0488 (8)	0.0453 (8)	0.0400 (7)	−0.0170 (6)	−0.0048 (6)	−0.0008 (6)
N4	0.0510 (7)	0.0487 (7)	0.0364 (6)	−0.0207 (5)	−0.0022 (5)	−0.0057 (5)
C5	0.0586 (9)	0.0655 (10)	0.0425 (8)	−0.0319 (8)	0.0027 (7)	−0.0102 (7)
C6	0.0678 (10)	0.0605 (9)	0.0385 (8)	−0.0287 (8)	−0.0011 (7)	−0.0065 (7)
C21	0.0503 (8)	0.0426 (7)	0.0391 (7)	−0.0187 (6)	−0.0051 (6)	−0.0021 (6)
C22	0.0480 (8)	0.0441 (8)	0.0441 (8)	−0.0156 (6)	−0.0054 (6)	−0.0032 (6)
C23	0.0658 (10)	0.0620 (10)	0.0452 (9)	−0.0237 (8)	−0.0026 (7)	−0.0134 (7)
C24	0.0649 (10)	0.0836 (12)	0.0523 (9)	−0.0336 (9)	−0.0114 (8)	−0.0148 (9)
C25	0.0497 (9)	0.0982 (13)	0.0662 (11)	−0.0248 (9)	−0.0088 (8)	−0.0183 (10)
C26	0.0517 (9)	0.0763 (11)	0.0549 (10)	−0.0196 (8)	−0.0007 (7)	−0.0200 (8)
O22	0.0496 (6)	0.0720 (7)	0.0595 (7)	−0.0088 (5)	−0.0046 (5)	−0.0237 (6)
C27	0.0582 (10)	0.0747 (12)	0.0849 (13)	−0.0085 (9)	0.0044 (9)	−0.0336 (10)
C31	0.0417 (7)	0.0445 (8)	0.0389 (7)	−0.0049 (6)	−0.0018 (6)	−0.0075 (6)
C32	0.0583 (9)	0.0425 (8)	0.0643 (10)	−0.0064 (7)	0.0005 (8)	−0.0127 (7)
C33	0.0775 (12)	0.0437 (9)	0.0853 (13)	0.0025 (8)	−0.0062 (10)	0.0020 (9)
C34	0.0550 (10)	0.0801 (13)	0.0749 (12)	0.0088 (9)	−0.0136 (9)	0.0064 (10)
C35	0.0473 (9)	0.0850 (13)	0.0590 (10)	−0.0117 (8)	−0.0116 (7)	0.0010 (9)
C36	0.0460 (8)	0.0585 (9)	0.0491 (9)	−0.0137 (7)	−0.0082 (6)	0.0011 (7)
C37	0.0462 (8)	0.0445 (8)	0.0573 (9)	−0.0121 (6)	−0.0131 (7)	−0.0091 (7)
O31	0.0594 (7)	0.0788 (8)	0.0667 (8)	−0.0227 (6)	−0.0250 (6)	0.0177 (6)
O32	0.0422 (6)	0.0544 (7)	0.0800 (8)	−0.0033 (5)	−0.0046 (5)	−0.0094 (6)
C38	0.0885 (13)	0.0510 (10)	0.1240 (18)	−0.0242 (10)	−0.0133 (12)	−0.0174 (11)

### 4-(2-Methoxyphenyl)piperazin-1-ium 2-methylbenzoate (VIII) Geometric parameters (Å, º)

**Table:**

N1—C6	1.4820 (18)	C25—C26	1.394 (2)
N1—C2	1.4866 (18)	C25—H25	0.9300
N1—H11	1.010 (15)	C26—H26	0.9300
N1—H12	0.963 (16)	O22—C27	1.4300 (19)
C2—C3	1.5094 (19)	C27—H27A	0.9600
C2—H2A	0.9700	C27—H27B	0.9600
C2—H2B	0.9700	C27—H27C	0.9600
C3—N4	1.4621 (17)	C31—C36	1.3861 (19)
C3—H3A	0.9700	C31—C32	1.395 (2)
C3—H3B	0.9700	C31—C37	1.508 (2)
N4—C21	1.4202 (18)	C32—C33	1.402 (2)
N4—C5	1.4592 (18)	C32—C38	1.511 (2)
C5—C6	1.508 (2)	C33—C34	1.359 (3)
C5—H5A	0.9700	C33—H33	0.9300
C5—H5B	0.9700	C34—C35	1.370 (3)
C6—H6A	0.9700	C34—H34	0.9300
C6—H6B	0.9700	C35—C36	1.387 (2)
C21—C26	1.379 (2)	C35—H35	0.9300
C21—C22	1.413 (2)	C36—H36	0.9300
C22—O22	1.3634 (16)	C37—O31	1.2552 (17)
C22—C23	1.381 (2)	C37—O32	1.2587 (16)
C23—C24	1.382 (2)	C38—H38A	0.9600
C23—H23	0.9300	C38—H38B	0.9600
C24—C25	1.368 (2)	C38—H38C	0.9600
C24—H24	0.9300		
			
C6—N1—C2	111.69 (11)	C25—C24—H24	119.8
C6—N1—H11	111.2 (8)	C23—C24—H24	119.8
C2—N1—H11	105.7 (8)	C24—C25—C26	119.46 (15)
C6—N1—H12	109.4 (8)	C24—C25—H25	120.3
C2—N1—H12	108.3 (9)	C26—C25—H25	120.3
H11—N1—H12	110.4 (12)	C21—C26—C25	121.52 (15)
N1—C2—C3	110.86 (11)	C21—C26—H26	119.2
N1—C2—H2A	109.5	C25—C26—H26	119.2
C3—C2—H2A	109.5	C22—O22—C27	117.89 (12)
N1—C2—H2B	109.5	O22—C27—H27A	109.5
C3—C2—H2B	109.5	O22—C27—H27B	109.5
H2A—C2—H2B	108.1	H27A—C27—H27B	109.5
N4—C3—C2	109.85 (11)	O22—C27—H27C	109.5
N4—C3—H3A	109.7	H27A—C27—H27C	109.5
C2—C3—H3A	109.7	H27B—C27—H27C	109.5
N4—C3—H3B	109.7	C36—C31—C32	119.20 (14)
C2—C3—H3B	109.7	C36—C31—C37	117.89 (13)
H3A—C3—H3B	108.2	C32—C31—C37	122.89 (13)
C21—N4—C5	117.07 (11)	C31—C32—C33	117.94 (15)
C21—N4—C3	113.60 (10)	C31—C32—C38	122.05 (15)
C5—N4—C3	109.82 (11)	C33—C32—C38	119.99 (16)
N4—C5—C6	109.00 (12)	C34—C33—C32	122.27 (17)
N4—C5—H5A	109.9	C34—C33—H33	118.9
C6—C5—H5A	109.9	C32—C33—H33	118.9
N4—C5—H5B	109.9	C33—C34—C35	119.66 (17)
C6—C5—H5B	109.9	C33—C34—H34	120.2
H5A—C5—H5B	108.3	C35—C34—H34	120.2
N1—C6—C5	110.75 (12)	C34—C35—C36	119.66 (17)
N1—C6—H6A	109.5	C34—C35—H35	120.2
C5—C6—H6A	109.5	C36—C35—H35	120.2
N1—C6—H6B	109.5	C31—C36—C35	121.23 (15)
C5—C6—H6B	109.5	C31—C36—H36	119.4
H6A—C6—H6B	108.1	C35—C36—H36	119.4
C26—C21—C22	118.20 (13)	O31—C37—O32	124.33 (14)
C26—C21—N4	123.60 (13)	O31—C37—C31	117.71 (13)
C22—C21—N4	118.18 (12)	O32—C37—C31	117.92 (13)
O22—C22—C23	124.39 (13)	C32—C38—H38A	109.5
O22—C22—C21	115.73 (13)	C32—C38—H38B	109.5
C23—C22—C21	119.88 (13)	H38A—C38—H38B	109.5
C22—C23—C24	120.54 (15)	C32—C38—H38C	109.5
C22—C23—H23	119.7	H38A—C38—H38C	109.5
C24—C23—H23	119.7	H38B—C38—H38C	109.5
C25—C24—C23	120.39 (15)		
			
C6—N1—C2—C3	−52.32 (16)	C22—C21—C26—C25	0.3 (2)
N1—C2—C3—N4	56.11 (15)	N4—C21—C26—C25	178.59 (14)
C2—C3—N4—C21	164.70 (11)	C24—C25—C26—C21	0.3 (3)
C2—C3—N4—C5	−62.04 (15)	C23—C22—O22—C27	4.9 (2)
C21—N4—C5—C6	−165.56 (12)	C21—C22—O22—C27	−176.31 (13)
C3—N4—C5—C6	62.98 (16)	C36—C31—C32—C33	−2.0 (2)
C2—N1—C6—C5	53.64 (16)	C37—C31—C32—C33	176.40 (15)
N4—C5—C6—N1	−58.51 (17)	C36—C31—C32—C38	176.65 (15)
C5—N4—C21—C26	−20.7 (2)	C37—C31—C32—C38	−4.9 (2)
C3—N4—C21—C26	109.01 (15)	C31—C32—C33—C34	2.0 (3)
C5—N4—C21—C22	157.56 (13)	C38—C32—C33—C34	−176.74 (18)
C3—N4—C21—C22	−72.74 (15)	C32—C33—C34—C35	−0.8 (3)
C26—C21—C22—O22	179.95 (13)	C33—C34—C35—C36	−0.3 (3)
N4—C21—C22—O22	1.60 (18)	C32—C31—C36—C35	1.0 (2)
C26—C21—C22—C23	−1.2 (2)	C37—C31—C36—C35	−177.50 (14)
N4—C21—C22—C23	−179.54 (12)	C34—C35—C36—C31	0.2 (2)
O22—C22—C23—C24	−179.86 (14)	C36—C31—C37—O31	−47.61 (19)
C21—C22—C23—C24	1.4 (2)	C32—C31—C37—O31	133.92 (15)
C22—C23—C24—C25	−0.7 (2)	C36—C31—C37—O32	130.15 (14)
C23—C24—C25—C26	−0.2 (3)	C32—C31—C37—O32	−48.3 (2)

### 4-(2-Methoxyphenyl)piperazin-1-ium 2-methylbenzoate (VIII) Hydrogen-bond geometry (Å, º)

**Table:**

*D*—H···*A*	*D*—H	H···*A*	*D*···*A*	*D*—H···*A*
N1—H11···O31	1.010 (15)	1.673 (15)	2.6696 (19)	168.6 (13)
N1—H12···O32^i^	0.963 (16)	1.745 (16)	2.7077 (17)	178.2 (10)

Symmetry code: (i) −*x*+1, −*y*+1, −*z*+1.

### 4-(2-Methoxyphenyl)piperazin-1-ium 4-aminobenzoate (IX) Crystal data

**Table:**

C_11_H_17_N_2_O^+^·C_7_H_6_NO_2_^−^	*F*(000) = 704
*M**_r_* = 329.39	*D*_x_ = 1.280 Mg m^−^^3^
Monoclinic, *P*2_1_/*c*	Mo *K*α radiation, λ = 0.71073 Å
*a* = 14.922 (1) Å	Cell parameters from 3671 reflections
*b* = 7.6951 (5) Å	θ = 2.7–27.8°
*c* = 15.560 (1) Å	µ = 0.09 mm^−^^1^
β = 106.911 (8)°	*T* = 296 K
*V* = 1709.4 (2) Å^3^	Plate, orange
*Z* = 4	0.48 × 0.44 × 0.16 mm

### 4-(2-Methoxyphenyl)piperazin-1-ium 4-aminobenzoate (IX) Data collection

**Table:**

Oxford Diffraction Xcalibur with Sapphire CCD diffractometer	3668 independent reflections
Radiation source: Enhance (Mo) X-ray Source	2606 reflections with *I* > 2σ(*I*)
Graphite monochromator	*R*_int_ = 0.014
ω scans	θ_max_ = 27.6°, θ_min_ = 2.7°
Absorption correction: multi-scan (CrysAlis RED; Oxford Diffraction, 2009)	*h* = −18→15
*T*_min_ = 0.830, *T*_max_ = 0.986	*k* = −5→9
6720 measured reflections	*l* = −17→20

### 4-(2-Methoxyphenyl)piperazin-1-ium 4-aminobenzoate (IX) Refinement

**Table:**

Refinement on *F*^2^	Hydrogen site location: mixed
Least-squares matrix: full	H atoms treated by a mixture of independent and constrained refinement
*R*[*F*^2^ > 2σ(*F*^2^)] = 0.039	*w* = 1/[σ^2^(*F*_o_^2^) + (0.0619*P*)^2^ + 0.0395*P*] where *P* = (*F*_o_^2^ + 2*F*_c_^2^)/3
*wR*(*F*^2^) = 0.112	(Δ/σ)_max_ < 0.001
*S* = 1.10	Δρ_max_ = 0.15 e Å^−^^3^
3668 reflections	Δρ_min_ = −0.24 e Å^−^^3^
231 parameters	Extinction correction: SHELXL, Fc^*^=kFc[1+0.001xFc^2^λ^3^/sin(2θ)]^-1/4^
0 restraints	Extinction coefficient: 0.019 (2)
Primary atom site location: difference Fourier map	

### 4-(2-Methoxyphenyl)piperazin-1-ium 4-aminobenzoate (IX) Special details

**Table:**

Experimental. Empirical absorption correction using spherical harmonics, implemented in SCALE3 ABSPACK scaling algorithm.
Geometry. All esds (except the esd in the dihedral angle between two l.s. planes) are estimated using the full covariance matrix. The cell esds are taken into account individually in the estimation of esds in distances, angles and torsion angles; correlations between esds in cell parameters are only used when they are defined by crystal symmetry. An approximate (isotropic) treatment of cell esds is used for estimating esds involving l.s. planes.

### 4-(2-Methoxyphenyl)piperazin-1-ium 4-aminobenzoate (IX) Fractional atomic coordinates and isotropic or equivalent isotropic displacement parameters (Å^2^)

**Table:**

	*x*	*y*	*z*	*U*_iso_*/*U*_eq_	
N1	0.60709 (8)	0.26835 (15)	0.52995 (8)	0.0431 (3)	
H11	0.5586 (10)	0.2606 (17)	0.4643 (10)	0.052*	
H12	0.5953 (10)	0.3661 (19)	0.5615 (10)	0.052*	
C2	0.70246 (9)	0.28650 (18)	0.51932 (9)	0.0442 (3)	
H2A	0.7056	0.3915	0.4858	0.053*	
H2B	0.7155	0.1883	0.4857	0.053*	
C3	0.77450 (9)	0.29453 (16)	0.60945 (8)	0.0385 (3)	
H3A	0.8364	0.3050	0.6015	0.046*	
H3B	0.7635	0.3963	0.6417	0.046*	
N4	0.77041 (7)	0.13685 (13)	0.66227 (7)	0.0376 (3)	
C5	0.67672 (9)	0.11904 (18)	0.67358 (9)	0.0433 (3)	
H5A	0.6642	0.2174	0.7074	0.052*	
H5B	0.6741	0.0143	0.7074	0.052*	
C6	0.60307 (9)	0.11039 (17)	0.58382 (9)	0.0458 (3)	
H6A	0.6131	0.0079	0.5515	0.055*	
H6B	0.5416	0.1013	0.5928	0.055*	
C21	0.84580 (9)	0.13068 (16)	0.74309 (9)	0.0409 (3)	
C22	0.93798 (9)	0.11685 (17)	0.73737 (10)	0.0460 (3)	
C23	1.01328 (10)	0.1198 (2)	0.81461 (11)	0.0600 (4)	
H23	1.0740	0.1142	0.8102	0.072*	
C24	0.99867 (13)	0.1311 (2)	0.89743 (11)	0.0714 (5)	
H24	1.0495	0.1345	0.9489	0.086*	
C25	0.90983 (13)	0.1373 (3)	0.90449 (11)	0.0732 (5)	
H25	0.9001	0.1412	0.9608	0.088*	
C26	0.83373 (11)	0.1379 (2)	0.82761 (9)	0.0559 (4)	
H26	0.7734	0.1432	0.8332	0.067*	
O22	0.94654 (7)	0.10004 (14)	0.65293 (7)	0.0603 (3)	
C27	1.03816 (12)	0.1063 (3)	0.64265 (13)	0.0762 (5)	
H27A	1.0342	0.0973	0.5801	0.114*	
H27B	1.0747	0.0114	0.6749	0.114*	
H27C	1.0675	0.2142	0.6660	0.114*	
C31	0.38418 (8)	0.30821 (15)	0.23323 (8)	0.0341 (3)	
C32	0.30092 (9)	0.39385 (17)	0.19135 (9)	0.0410 (3)	
H32	0.2734	0.4636	0.2255	0.049*	
C33	0.25815 (9)	0.37762 (17)	0.10033 (9)	0.0429 (3)	
H33	0.2021	0.4355	0.0741	0.051*	
C34	0.29810 (9)	0.27553 (16)	0.04752 (8)	0.0373 (3)	
C35	0.38187 (9)	0.19061 (17)	0.08919 (8)	0.0395 (3)	
H35	0.4102	0.1226	0.0551	0.047*	
C36	0.42342 (9)	0.20608 (16)	0.18038 (9)	0.0382 (3)	
H36	0.4789	0.1468	0.2069	0.046*	
C37	0.43113 (9)	0.32513 (16)	0.33203 (8)	0.0379 (3)	
O31	0.50355 (7)	0.23691 (14)	0.36411 (6)	0.0627 (3)	
O32	0.39544 (7)	0.42404 (13)	0.37713 (6)	0.0526 (3)	
N34	0.25457 (10)	0.25415 (18)	−0.04368 (8)	0.0501 (3)	
H341	0.2158 (11)	0.340 (2)	−0.0658 (11)	0.060*	
H342	0.2928 (11)	0.211 (2)	−0.0750 (10)	0.060*	

### 4-(2-Methoxyphenyl)piperazin-1-ium 4-aminobenzoate (IX) Atomic displacement parameters (Å^2^)

**Table:**

	*U*^11^	*U*^22^	*U*^33^	*U*^12^	*U*^13^	*U*^23^
N1	0.0392 (6)	0.0463 (7)	0.0372 (6)	0.0090 (5)	0.0006 (5)	−0.0055 (5)
C2	0.0476 (8)	0.0477 (8)	0.0356 (7)	0.0105 (6)	0.0093 (6)	0.0029 (6)
C3	0.0364 (7)	0.0412 (7)	0.0369 (7)	0.0019 (5)	0.0090 (5)	0.0051 (5)
N4	0.0319 (5)	0.0418 (6)	0.0361 (6)	0.0015 (4)	0.0054 (4)	0.0063 (5)
C5	0.0370 (7)	0.0447 (7)	0.0471 (8)	−0.0015 (5)	0.0106 (6)	0.0064 (6)
C6	0.0352 (7)	0.0449 (8)	0.0523 (9)	0.0009 (5)	0.0048 (6)	−0.0043 (6)
C21	0.0396 (7)	0.0402 (7)	0.0385 (7)	0.0019 (5)	0.0045 (6)	0.0068 (6)
C22	0.0402 (7)	0.0477 (8)	0.0459 (8)	0.0075 (6)	0.0061 (6)	0.0066 (6)
C23	0.0402 (8)	0.0653 (10)	0.0642 (11)	0.0072 (7)	−0.0012 (7)	0.0102 (8)
C24	0.0632 (11)	0.0846 (13)	0.0482 (10)	0.0057 (9)	−0.0124 (8)	0.0141 (9)
C25	0.0752 (12)	0.0985 (14)	0.0378 (9)	0.0081 (10)	0.0037 (8)	0.0151 (9)
C26	0.0529 (9)	0.0727 (10)	0.0402 (8)	0.0057 (7)	0.0105 (7)	0.0130 (7)
O22	0.0416 (6)	0.0838 (8)	0.0561 (7)	0.0162 (5)	0.0150 (5)	0.0020 (6)
C27	0.0525 (10)	0.0953 (14)	0.0880 (14)	0.0157 (9)	0.0320 (9)	0.0088 (11)
C31	0.0373 (6)	0.0343 (6)	0.0318 (7)	−0.0043 (5)	0.0119 (5)	−0.0014 (5)
C32	0.0437 (7)	0.0454 (7)	0.0371 (7)	0.0048 (5)	0.0165 (6)	−0.0029 (6)
C33	0.0371 (7)	0.0497 (8)	0.0402 (8)	0.0052 (6)	0.0086 (6)	0.0023 (6)
C34	0.0392 (7)	0.0411 (7)	0.0311 (6)	−0.0106 (5)	0.0093 (5)	−0.0014 (5)
C35	0.0405 (7)	0.0435 (7)	0.0375 (7)	−0.0041 (5)	0.0162 (6)	−0.0115 (6)
C36	0.0346 (6)	0.0407 (7)	0.0380 (7)	0.0009 (5)	0.0086 (5)	−0.0050 (6)
C37	0.0433 (7)	0.0375 (6)	0.0326 (7)	−0.0062 (5)	0.0108 (6)	−0.0023 (5)
O31	0.0634 (7)	0.0731 (7)	0.0387 (6)	0.0183 (5)	−0.0053 (5)	−0.0091 (5)
O32	0.0664 (7)	0.0579 (6)	0.0364 (5)	0.0017 (5)	0.0197 (5)	−0.0099 (4)
N34	0.0537 (8)	0.0590 (8)	0.0337 (6)	−0.0036 (6)	0.0067 (5)	−0.0047 (6)

### 4-(2-Methoxyphenyl)piperazin-1-ium 4-aminobenzoate (IX) Geometric parameters (Å, º)

**Table:**

N1—C2	1.4864 (18)	C24—H24	0.9300
N1—C6	1.4875 (18)	C25—C26	1.389 (2)
N1—H11	1.067 (14)	C25—H25	0.9300
N1—H12	0.942 (15)	C26—H26	0.9300
C2—C3	1.4997 (16)	O22—C27	1.4228 (18)
C2—H2A	0.9700	C27—H27A	0.9600
C2—H2B	0.9700	C27—H27B	0.9600
C3—N4	1.4768 (15)	C27—H27C	0.9600
C3—H3A	0.9700	C31—C36	1.3844 (17)
C3—H3B	0.9700	C31—C32	1.3897 (17)
N4—C21	1.4235 (16)	C31—C37	1.4982 (16)
N4—C5	1.4654 (16)	C32—C33	1.3796 (18)
C5—C6	1.5067 (18)	C32—H32	0.9300
C5—H5A	0.9700	C33—C34	1.3902 (18)
C5—H5B	0.9700	C33—H33	0.9300
C6—H6A	0.9700	C34—N34	1.3881 (16)
C6—H6B	0.9700	C34—C35	1.3923 (18)
C21—C26	1.380 (2)	C35—C36	1.3784 (17)
C21—C22	1.4079 (19)	C35—H35	0.9300
C22—O22	1.3627 (17)	C36—H36	0.9300
C22—C23	1.3863 (19)	C37—O31	1.2506 (16)
C23—C24	1.371 (2)	C37—O32	1.2542 (15)
C23—H23	0.9300	N34—H341	0.877 (16)
C24—C25	1.363 (2)	N34—H342	0.913 (16)
			
C2—N1—C6	109.66 (10)	C22—C23—H23	119.8
C2—N1—H11	107.7 (8)	C25—C24—C23	120.16 (15)
C6—N1—H11	111.5 (7)	C25—C24—H24	119.9
C2—N1—H12	108.2 (9)	C23—C24—H24	119.9
C6—N1—H12	108.3 (9)	C24—C25—C26	120.10 (16)
H11—N1—H12	111.5 (11)	C24—C25—H25	119.9
N1—C2—C3	110.42 (11)	C26—C25—H25	120.0
N1—C2—H2A	109.6	C21—C26—C25	121.33 (15)
C3—C2—H2A	109.6	C21—C26—H26	119.3
N1—C2—H2B	109.6	C25—C26—H26	119.3
C3—C2—H2B	109.6	C22—O22—C27	117.90 (12)
H2A—C2—H2B	108.1	O22—C27—H27A	109.5
N4—C3—C2	110.62 (10)	O22—C27—H27B	109.5
N4—C3—H3A	109.5	H27A—C27—H27B	109.5
C2—C3—H3A	109.5	O22—C27—H27C	109.5
N4—C3—H3B	109.5	H27A—C27—H27C	109.5
C2—C3—H3B	109.5	H27B—C27—H27C	109.5
H3A—C3—H3B	108.1	C36—C31—C32	117.75 (11)
C21—N4—C5	115.29 (10)	C36—C31—C37	120.43 (11)
C21—N4—C3	111.66 (9)	C32—C31—C37	121.82 (11)
C5—N4—C3	109.81 (9)	C33—C32—C31	121.46 (12)
N4—C5—C6	110.91 (11)	C33—C32—H32	119.3
N4—C5—H5A	109.5	C31—C32—H32	119.3
C6—C5—H5A	109.5	C32—C33—C34	120.54 (12)
N4—C5—H5B	109.5	C32—C33—H33	119.7
C6—C5—H5B	109.5	C34—C33—H33	119.7
H5A—C5—H5B	108.0	N34—C34—C33	121.17 (12)
N1—C6—C5	110.38 (10)	N34—C34—C35	120.69 (12)
N1—C6—H6A	109.6	C33—C34—C35	118.11 (11)
C5—C6—H6A	109.6	C36—C35—C34	120.86 (11)
N1—C6—H6B	109.6	C36—C35—H35	119.6
C5—C6—H6B	109.6	C34—C35—H35	119.6
H6A—C6—H6B	108.1	C35—C36—C31	121.27 (12)
C26—C21—C22	117.66 (13)	C35—C36—H36	119.4
C26—C21—N4	123.51 (12)	C31—C36—H36	119.4
C22—C21—N4	118.83 (12)	O31—C37—O32	124.36 (12)
O22—C22—C23	123.91 (13)	O31—C37—C31	117.02 (11)
O22—C22—C21	115.81 (12)	O32—C37—C31	118.62 (11)
C23—C22—C21	120.28 (14)	C34—N34—H341	111.8 (11)
C24—C23—C22	120.38 (15)	C34—N34—H342	114.2 (10)
C24—C23—H23	119.8	H341—N34—H342	120.4 (15)
			
C6—N1—C2—C3	−57.43 (14)	C22—C21—C26—C25	2.1 (2)
N1—C2—C3—N4	58.53 (13)	N4—C21—C26—C25	−177.79 (13)
C2—C3—N4—C21	172.34 (11)	C24—C25—C26—C21	0.6 (3)
C2—C3—N4—C5	−58.48 (13)	C23—C22—O22—C27	−7.6 (2)
C21—N4—C5—C6	−174.66 (10)	C21—C22—O22—C27	172.72 (13)
C3—N4—C5—C6	58.16 (13)	C36—C31—C32—C33	0.31 (19)
C2—N1—C6—C5	56.92 (14)	C37—C31—C32—C33	179.98 (12)
N4—C5—C6—N1	−57.96 (14)	C31—C32—C33—C34	−0.6 (2)
C5—N4—C21—C26	−10.87 (18)	C32—C33—C34—N34	178.21 (12)
C3—N4—C21—C26	115.36 (14)	C32—C33—C34—C35	0.14 (19)
C5—N4—C21—C22	169.26 (11)	N34—C34—C35—C36	−177.43 (12)
C3—N4—C21—C22	−64.51 (14)	C33—C34—C35—C36	0.65 (19)
C26—C21—C22—O22	176.36 (13)	C34—C35—C36—C31	−0.97 (19)
N4—C21—C22—O22	−3.76 (17)	C32—C31—C36—C35	0.48 (19)
C26—C21—C22—C23	−3.3 (2)	C37—C31—C36—C35	−179.20 (11)
N4—C21—C22—C23	176.56 (11)	C36—C31—C37—O31	−3.44 (17)
O22—C22—C23—C24	−177.72 (15)	C32—C31—C37—O31	176.90 (13)
C21—C22—C23—C24	1.9 (2)	C36—C31—C37—O32	177.29 (12)
C22—C23—C24—C25	0.8 (3)	C32—C31—C37—O32	−2.37 (18)
C23—C24—C25—C26	−2.1 (3)		

### 4-(2-Methoxyphenyl)piperazin-1-ium 4-aminobenzoate (IX) Hydrogen-bond geometry (Å, º)

**Table:**

*D*—H···*A*	*D*—H	H···*A*	*D*···*A*	*D*—H···*A*
N1—H11···O31	1.068 (15)	1.547 (15)	2.6048 (15)	169.7 (14)
N1—H12···O32^i^	0.942 (15)	1.861 (15)	2.7797 (15)	164.4 (14)
N34—H342···O32^ii^	0.914 (16)	2.155 (16)	3.0535 (18)	167.5 (14)

Symmetry codes: (i) −*x*+1, −*y*+1, −*z*+1; (ii) *x*, −*y*+1/2, *z*−1/2.

### 4-(2-Methoxyphenyl)piperazin-1-ium 4-nitrobenzoate (X) Crystal data

**Table:**

C_11_H_17_N_2_O^+^·C_7_H_4_NO_4_^−^	*F*(000) = 760
*M**_r_* = 359.38	*D*_x_ = 1.344 Mg m^−^^3^
Monoclinic, *P*2_1_/*c*	Mo *K*α radiation, λ = 0.71073 Å
*a* = 7.5174 (5) Å	Cell parameters from 3934 reflections
*b* = 7.9761 (5) Å	θ = 2.7–27.9°
*c* = 29.860 (2) Å	µ = 0.10 mm^−^^1^
β = 97.322 (6)°	*T* = 296 K
*V* = 1775.8 (2) Å^3^	Block, yellow
*Z* = 4	0.50 × 0.50 × 0.40 mm

### 4-(2-Methoxyphenyl)piperazin-1-ium 4-nitrobenzoate (X) Data collection

**Table:**

Oxford Diffraction Xcalibur with Sapphire CCD diffractometer	3934 independent reflections
Radiation source: Enhance (Mo) X-ray Source	2879 reflections with *I* > 2σ(*I*)
Graphite monochromator	*R*_int_ = 0.019
ω scans	θ_max_ = 27.9°, θ_min_ = 2.7°
Absorption correction: multi-scan (CrysAlis RED; Oxford Diffraction, 2009)	*h* = −9→8
*T*_min_ = 0.855, *T*_max_ = 0.961	*k* = −10→10
13660 measured reflections	*l* = −38→38

### 4-(2-Methoxyphenyl)piperazin-1-ium 4-nitrobenzoate (X) Refinement

**Table:**

Refinement on *F*^2^	Hydrogen site location: mixed
Least-squares matrix: full	H atoms treated by a mixture of independent and constrained refinement
*R*[*F*^2^ > 2σ(*F*^2^)] = 0.040	*w* = 1/[σ^2^(*F*_o_^2^) + (0.0457*P*)^2^ + 0.4848*P*] where *P* = (*F*_o_^2^ + 2*F*_c_^2^)/3
*wR*(*F*^2^) = 0.111	(Δ/σ)_max_ = 0.001
*S* = 1.03	Δρ_max_ = 0.17 e Å^−^^3^
3934 reflections	Δρ_min_ = −0.15 e Å^−^^3^
242 parameters	Extinction correction: SHELXL, Fc^*^=kFc[1+0.001xFc^2^λ^3^/sin(2θ)]^-1/4^
0 restraints	Extinction coefficient: 0.0175 (15)
Primary atom site location: difference Fourier map	

### 4-(2-Methoxyphenyl)piperazin-1-ium 4-nitrobenzoate (X) Special details

**Table:**

Experimental. Empirical absorption correction using spherical harmonics, implemented in SCALE3 ABSPACK scaling algorithm.
Geometry. All esds (except the esd in the dihedral angle between two l.s. planes) are estimated using the full covariance matrix. The cell esds are taken into account individually in the estimation of esds in distances, angles and torsion angles; correlations between esds in cell parameters are only used when they are defined by crystal symmetry. An approximate (isotropic) treatment of cell esds is used for estimating esds involving l.s. planes.

### 4-(2-Methoxyphenyl)piperazin-1-ium 4-nitrobenzoate (X) Fractional atomic coordinates and isotropic or equivalent isotropic displacement parameters (Å^2^)

**Table:**

	*x*	*y*	*z*	*U*_iso_*/*U*_eq_	
N1	0.44452 (18)	0.51582 (18)	0.42036 (4)	0.0456 (3)	
H11	0.359 (2)	0.579 (2)	0.4356 (6)	0.055*	
H12	0.486 (2)	0.426 (2)	0.4397 (6)	0.055*	
C2	0.5986 (2)	0.6202 (2)	0.41168 (5)	0.0509 (4)	
H2A	0.6633	0.6571	0.4402	0.061*	
H2B	0.5558	0.7188	0.3946	0.061*	
C3	0.72308 (19)	0.5222 (2)	0.38567 (5)	0.0445 (4)	
H3A	0.8229	0.5926	0.3799	0.053*	
H3B	0.7707	0.4266	0.4034	0.053*	
N4	0.62657 (15)	0.46482 (15)	0.34309 (4)	0.0388 (3)	
C5	0.47731 (19)	0.3566 (2)	0.35153 (5)	0.0431 (3)	
H5A	0.5234	0.2588	0.3685	0.052*	
H5B	0.4140	0.3187	0.3230	0.052*	
C6	0.3495 (2)	0.4492 (2)	0.37760 (5)	0.0468 (4)	
H6A	0.2950	0.5410	0.3595	0.056*	
H6B	0.2548	0.3740	0.3841	0.056*	
C21	0.73290 (19)	0.39011 (18)	0.31211 (5)	0.0405 (3)	
C22	0.6595 (2)	0.3763 (2)	0.26645 (5)	0.0474 (4)	
C23	0.7580 (3)	0.3012 (2)	0.23580 (6)	0.0602 (5)	
H23	0.7093	0.2922	0.2057	0.072*	
C24	0.9269 (3)	0.2400 (3)	0.24948 (7)	0.0697 (5)	
H24	0.9919	0.1898	0.2286	0.084*	
C25	1.0000 (2)	0.2525 (3)	0.29365 (7)	0.0695 (5)	
H25	1.1143	0.2108	0.3029	0.083*	
C26	0.9025 (2)	0.3280 (2)	0.32471 (6)	0.0546 (4)	
H26	0.9533	0.3368	0.3547	0.066*	
O22	0.49191 (17)	0.43933 (18)	0.25585 (4)	0.0657 (4)	
C27	0.4027 (3)	0.4122 (3)	0.21158 (6)	0.0739 (6)	
H27A	0.2862	0.4633	0.2088	0.111*	
H27B	0.4715	0.4612	0.1900	0.111*	
H27C	0.3902	0.2940	0.2061	0.111*	
C31	0.16123 (19)	0.82005 (18)	0.52883 (4)	0.0392 (3)	
C32	−0.0213 (2)	0.79178 (19)	0.52353 (5)	0.0450 (4)	
H32	−0.0716	0.7179	0.5014	0.054*	
C33	−0.1305 (2)	0.8720 (2)	0.55077 (5)	0.0458 (4)	
H33	−0.2535	0.8531	0.5473	0.055*	
C34	−0.05131 (19)	0.98058 (18)	0.58316 (5)	0.0414 (3)	
C35	0.1295 (2)	1.01111 (19)	0.58934 (5)	0.0441 (4)	
H35	0.1794	1.0853	0.6115	0.053*	
C36	0.2358 (2)	0.92921 (19)	0.56193 (5)	0.0435 (3)	
H36	0.3590	0.9477	0.5658	0.052*	
C37	0.2801 (2)	0.73155 (19)	0.49920 (5)	0.0458 (4)	
O31	0.20537 (17)	0.67769 (17)	0.46209 (4)	0.0656 (4)	
O32	0.44235 (15)	0.71810 (15)	0.51349 (4)	0.0570 (3)	
N34	−0.1638 (2)	1.06966 (19)	0.61219 (5)	0.0541 (4)	
O41	−0.32042 (19)	1.0270 (2)	0.61086 (5)	0.0826 (4)	
O42	−0.09646 (19)	1.18199 (18)	0.63615 (4)	0.0731 (4)	

### 4-(2-Methoxyphenyl)piperazin-1-ium 4-nitrobenzoate (X) Atomic displacement parameters (Å^2^)

**Table:**

	*U*^11^	*U*^22^	*U*^33^	*U*^12^	*U*^13^	*U*^23^
N1	0.0481 (7)	0.0554 (8)	0.0341 (6)	0.0041 (6)	0.0078 (5)	−0.0034 (6)
C2	0.0574 (9)	0.0535 (9)	0.0410 (8)	−0.0073 (8)	0.0034 (7)	−0.0110 (7)
C3	0.0444 (8)	0.0490 (9)	0.0395 (7)	−0.0089 (7)	0.0027 (6)	−0.0033 (7)
N4	0.0394 (6)	0.0450 (7)	0.0321 (6)	−0.0041 (5)	0.0041 (5)	−0.0029 (5)
C5	0.0409 (8)	0.0510 (9)	0.0372 (7)	−0.0061 (7)	0.0039 (6)	−0.0064 (6)
C6	0.0412 (8)	0.0611 (10)	0.0377 (7)	−0.0022 (7)	0.0038 (6)	−0.0026 (7)
C21	0.0424 (8)	0.0419 (8)	0.0383 (7)	−0.0049 (6)	0.0089 (6)	−0.0004 (6)
C22	0.0532 (9)	0.0523 (9)	0.0375 (7)	0.0025 (7)	0.0093 (6)	0.0007 (7)
C23	0.0696 (11)	0.0717 (12)	0.0422 (9)	0.0004 (9)	0.0182 (8)	−0.0069 (8)
C24	0.0610 (11)	0.0838 (14)	0.0703 (12)	−0.0019 (10)	0.0314 (10)	−0.0169 (11)
C25	0.0408 (9)	0.0878 (14)	0.0816 (13)	0.0024 (9)	0.0144 (9)	−0.0132 (11)
C26	0.0400 (8)	0.0695 (11)	0.0543 (9)	−0.0039 (8)	0.0058 (7)	−0.0040 (8)
O22	0.0696 (7)	0.0899 (10)	0.0349 (6)	0.0288 (7)	−0.0039 (5)	−0.0076 (6)
C27	0.0843 (13)	0.0919 (15)	0.0406 (9)	0.0168 (12)	−0.0106 (9)	−0.0046 (9)
C31	0.0456 (8)	0.0401 (8)	0.0318 (7)	0.0060 (6)	0.0049 (6)	0.0060 (6)
C32	0.0504 (9)	0.0470 (8)	0.0359 (7)	−0.0014 (7)	−0.0010 (6)	0.0003 (6)
C33	0.0392 (8)	0.0526 (9)	0.0454 (8)	−0.0017 (7)	0.0052 (6)	0.0076 (7)
C34	0.0467 (8)	0.0430 (8)	0.0360 (7)	0.0057 (7)	0.0121 (6)	0.0071 (6)
C35	0.0498 (9)	0.0438 (8)	0.0385 (7)	−0.0012 (7)	0.0044 (6)	−0.0019 (6)
C36	0.0402 (8)	0.0468 (8)	0.0432 (8)	0.0014 (7)	0.0046 (6)	0.0019 (7)
C37	0.0556 (9)	0.0455 (8)	0.0372 (7)	0.0100 (7)	0.0089 (7)	0.0053 (7)
O31	0.0698 (8)	0.0835 (9)	0.0428 (6)	0.0201 (7)	0.0044 (6)	−0.0148 (6)
O32	0.0520 (7)	0.0674 (8)	0.0526 (7)	0.0152 (6)	0.0111 (5)	0.0023 (6)
N34	0.0606 (9)	0.0598 (9)	0.0456 (7)	0.0109 (7)	0.0207 (6)	0.0082 (7)
O41	0.0611 (8)	0.1020 (11)	0.0923 (10)	0.0029 (8)	0.0395 (7)	−0.0035 (9)
O42	0.0905 (10)	0.0772 (9)	0.0558 (7)	0.0075 (8)	0.0258 (7)	−0.0159 (7)

### 4-(2-Methoxyphenyl)piperazin-1-ium 4-nitrobenzoate (X) Geometric parameters (Å, º)

**Table:**

N1—C2	1.476 (2)	C24—H24	0.9300
N1—C6	1.4798 (18)	C25—C26	1.390 (2)
N1—H11	0.974 (18)	C25—H25	0.9300
N1—H12	0.947 (18)	C26—H26	0.9300
C2—C3	1.508 (2)	O22—C27	1.4204 (19)
C2—H2A	0.9700	C27—H27A	0.9600
C2—H2B	0.9700	C27—H27B	0.9600
C3—N4	1.4551 (17)	C27—H27C	0.9600
C3—H3A	0.9700	C31—C32	1.380 (2)
C3—H3B	0.9700	C31—C36	1.381 (2)
N4—C21	1.4275 (18)	C31—C37	1.510 (2)
N4—C5	1.4626 (18)	C32—C33	1.384 (2)
C5—C6	1.505 (2)	C32—H32	0.9300
C5—H5A	0.9700	C33—C34	1.376 (2)
C5—H5B	0.9700	C33—H33	0.9300
C6—H6A	0.9700	C34—C35	1.370 (2)
C6—H6B	0.9700	C34—N34	1.4690 (19)
C21—C26	1.375 (2)	C35—C36	1.379 (2)
C21—C22	1.409 (2)	C35—H35	0.9300
C22—O22	1.3562 (19)	C36—H36	0.9300
C22—C23	1.384 (2)	C37—O32	1.2443 (18)
C23—C24	1.373 (3)	C37—O31	1.2528 (19)
C23—H23	0.9300	N34—O42	1.2157 (19)
C24—C25	1.366 (3)	N34—O41	1.2217 (19)
			
C2—N1—C6	110.74 (11)	C22—C23—H23	119.7
C2—N1—H11	111.7 (10)	C25—C24—C23	120.23 (17)
C6—N1—H11	108.4 (10)	C25—C24—H24	119.9
C2—N1—H12	109.0 (10)	C23—C24—H24	119.9
C6—N1—H12	109.8 (10)	C24—C25—C26	119.65 (17)
H11—N1—H12	107.1 (14)	C24—C25—H25	120.2
N1—C2—C3	110.53 (13)	C26—C25—H25	120.2
N1—C2—H2A	109.5	C21—C26—C25	121.52 (16)
C3—C2—H2A	109.5	C21—C26—H26	119.2
N1—C2—H2B	109.5	C25—C26—H26	119.2
C3—C2—H2B	109.5	C22—O22—C27	118.37 (13)
H2A—C2—H2B	108.1	O22—C27—H27A	109.5
N4—C3—C2	109.89 (12)	O22—C27—H27B	109.5
N4—C3—H3A	109.7	H27A—C27—H27B	109.5
C2—C3—H3A	109.7	O22—C27—H27C	109.5
N4—C3—H3B	109.7	H27A—C27—H27C	109.5
C2—C3—H3B	109.7	H27B—C27—H27C	109.5
H3A—C3—H3B	108.2	C32—C31—C36	119.48 (13)
C21—N4—C3	116.07 (11)	C32—C31—C37	120.70 (13)
C21—N4—C5	111.75 (11)	C36—C31—C37	119.81 (13)
C3—N4—C5	110.07 (11)	C31—C32—C33	120.84 (14)
N4—C5—C6	110.72 (12)	C31—C32—H32	119.6
N4—C5—H5A	109.5	C33—C32—H32	119.6
C6—C5—H5A	109.5	C34—C33—C32	117.93 (14)
N4—C5—H5B	109.5	C34—C33—H33	121.0
C6—C5—H5B	109.5	C32—C33—H33	121.0
H5A—C5—H5B	108.1	C35—C34—C33	122.65 (14)
N1—C6—C5	110.67 (12)	C35—C34—N34	118.04 (14)
N1—C6—H6A	109.5	C33—C34—N34	119.30 (14)
C5—C6—H6A	109.5	C34—C35—C36	118.40 (14)
N1—C6—H6B	109.5	C34—C35—H35	120.8
C5—C6—H6B	109.5	C36—C35—H35	120.8
H6A—C6—H6B	108.1	C35—C36—C31	120.68 (14)
C26—C21—C22	118.14 (14)	C35—C36—H36	119.7
C26—C21—N4	123.33 (13)	C31—C36—H36	119.7
C22—C21—N4	118.52 (13)	O32—C37—O31	125.68 (14)
O22—C22—C23	124.42 (14)	O32—C37—C31	117.82 (13)
O22—C22—C21	115.75 (13)	O31—C37—C31	116.50 (14)
C23—C22—C21	119.83 (15)	O42—N34—O41	123.48 (15)
C24—C23—C22	120.63 (16)	O42—N34—C34	118.43 (14)
C24—C23—H23	119.7	O41—N34—C34	118.09 (15)
			
C6—N1—C2—C3	−55.83 (17)	C24—C25—C26—C21	0.4 (3)
N1—C2—C3—N4	58.62 (16)	C23—C22—O22—C27	6.8 (3)
C2—C3—N4—C21	171.70 (12)	C21—C22—O22—C27	−172.93 (16)
C2—C3—N4—C5	−60.13 (16)	C36—C31—C32—C33	0.3 (2)
C21—N4—C5—C6	−170.14 (12)	C37—C31—C32—C33	179.66 (13)
C3—N4—C5—C6	59.35 (15)	C31—C32—C33—C34	0.1 (2)
C2—N1—C6—C5	54.67 (17)	C32—C33—C34—C35	−0.2 (2)
N4—C5—C6—N1	−56.40 (16)	C32—C33—C34—N34	179.10 (13)
C3—N4—C21—C26	19.2 (2)	C33—C34—C35—C36	−0.1 (2)
C5—N4—C21—C26	−108.17 (16)	N34—C34—C35—C36	−179.38 (13)
C3—N4—C21—C22	−162.02 (14)	C34—C35—C36—C31	0.5 (2)
C5—N4—C21—C22	70.65 (17)	C32—C31—C36—C35	−0.6 (2)
C26—C21—C22—O22	179.94 (15)	C37—C31—C36—C35	−179.95 (13)
N4—C21—C22—O22	1.1 (2)	C32—C31—C37—O32	−157.78 (15)
C26—C21—C22—C23	0.2 (2)	C36—C31—C37—O32	21.5 (2)
N4—C21—C22—C23	−178.64 (15)	C32—C31—C37—O31	22.1 (2)
O22—C22—C23—C24	−179.71 (18)	C36—C31—C37—O31	−158.53 (15)
C21—C22—C23—C24	0.0 (3)	C35—C34—N34—O42	9.5 (2)
C22—C23—C24—C25	0.0 (3)	C33—C34—N34—O42	−169.80 (14)
C23—C24—C25—C26	−0.1 (3)	C35—C34—N34—O41	−170.64 (15)
C22—C21—C26—C25	−0.4 (3)	C33—C34—N34—O41	10.0 (2)
N4—C21—C26—C25	178.42 (16)		

### 4-(2-Methoxyphenyl)piperazin-1-ium 4-nitrobenzoate (X) Hydrogen-bond geometry (Å, º)

**Table:**

*D*—H···*A*	*D*—H	H···*A*	*D*···*A*	*D*—H···*A*
N1—H11···O31	0.974 (16)	1.677 (16)	2.6500 (19)	176.8 (15)
N1—H11···O32	0.974 (16)	2.581 (17)	3.2169 (17)	123.0 (12)
N1—H12···O32^i^	0.948 (17)	1.837 (17)	2.7709 (18)	168.2 (16)

Symmetry code: (i) −*x*+1, −*y*+1, −*z*+1.

### 4-(2-Methoxyphenyl)piperazin-1-ium 3,5-dinitrobenzoate dihydrate (XI) Crystal data

**Table:**

C_11_H_17_N_2_O^+^·C_7_H_3_N_2_O_6_^−^·2H_2_O	*Z* = 2
*M**_r_* = 440.41	*F*(000) = 464
Triclinic, *P*1	*D*_x_ = 1.405 Mg m^−^^3^
*a* = 7.8448 (6) Å	Mo *K*α radiation, λ = 0.71073 Å
*b* = 11.4635 (9) Å	Cell parameters from 4419 reflections
*c* = 12.0747 (9) Å	θ = 2.6–27.7°
α = 94.406 (7)°	µ = 0.11 mm^−^^1^
β = 105.075 (8)°	*T* = 296 K
γ = 93.717 (7)°	Block, yellow
*V* = 1041.33 (14) Å^3^	0.48 × 0.48 × 0.44 mm

### 4-(2-Methoxyphenyl)piperazin-1-ium 3,5-dinitrobenzoate dihydrate (XI) Data collection

**Table:**

Oxford Diffraction Xcalibur with Sapphire CCD diffractometer	4419 independent reflections
Radiation source: Enhance (Mo) X-ray Source	3409 reflections with *I* > 2σ(*I*)
Graphite monochromator	*R*_int_ = 0.016
ω scans	θ_max_ = 27.7°, θ_min_ = 2.6°
Absorption correction: multi-scan (CrysAlis RED; Oxford Diffraction, 2009)	*h* = −9→10
*T*_min_ = 0.892, *T*_max_ = 0.951	*k* = −9→14
7353 measured reflections	*l* = −15→14

### 4-(2-Methoxyphenyl)piperazin-1-ium 3,5-dinitrobenzoate dihydrate (XI) Refinement

**Table:**

Refinement on *F*^2^	Hydrogen site location: mixed
Least-squares matrix: full	H atoms treated by a mixture of independent and constrained refinement
*R*[*F*^2^ > 2σ(*F*^2^)] = 0.039	*w* = 1/[σ^2^(*F*_o_^2^) + (0.0554*P*)^2^ + 0.1343*P*] where *P* = (*F*_o_^2^ + 2*F*_c_^2^)/3
*wR*(*F*^2^) = 0.108	(Δ/σ)_max_ < 0.001
*S* = 1.06	Δρ_max_ = 0.23 e Å^−^^3^
4419 reflections	Δρ_min_ = −0.17 e Å^−^^3^
300 parameters	Extinction correction: SHELXL, Fc^*^=kFc[1+0.001xFc^2^λ^3^/sin(2θ)]^-1/4^
0 restraints	Extinction coefficient: 0.052 (4)
Primary atom site location: difference Fourier map	

### 4-(2-Methoxyphenyl)piperazin-1-ium 3,5-dinitrobenzoate dihydrate (XI) Special details

**Table:**

Experimental. Empirical absorption correction using spherical harmonics, implemented in SCALE3 ABSPACK scaling algorithm.
Geometry. All esds (except the esd in the dihedral angle between two l.s. planes) are estimated using the full covariance matrix. The cell esds are taken into account individually in the estimation of esds in distances, angles and torsion angles; correlations between esds in cell parameters are only used when they are defined by crystal symmetry. An approximate (isotropic) treatment of cell esds is used for estimating esds involving l.s. planes.

### 4-(2-Methoxyphenyl)piperazin-1-ium 3,5-dinitrobenzoate dihydrate (XI) Fractional atomic coordinates and isotropic or equivalent isotropic displacement parameters (Å^2^)

**Table:**

	*x*	*y*	*z*	*U*_iso_*/*U*_eq_	
N1	0.65966 (17)	0.38158 (10)	0.34243 (10)	0.0387 (3)	
H11	0.718 (2)	0.3310 (14)	0.3931 (14)	0.046*	
H12	0.541 (2)	0.3638 (13)	0.3304 (13)	0.046*	
C2	0.7170 (2)	0.37246 (12)	0.23459 (12)	0.0437 (3)	
H2A	0.6800	0.2946	0.1947	0.052*	
H2B	0.8452	0.3838	0.2531	0.052*	
C3	0.63768 (19)	0.46357 (11)	0.15669 (11)	0.0383 (3)	
H3A	0.6811	0.4588	0.0884	0.046*	
H3B	0.5097	0.4481	0.1325	0.046*	
N4	0.68560 (14)	0.58186 (8)	0.21770 (8)	0.0317 (2)	
C5	0.61652 (19)	0.58963 (11)	0.31932 (11)	0.0374 (3)	
H5A	0.4888	0.5723	0.2961	0.045*	
H5B	0.6431	0.6686	0.3579	0.045*	
C6	0.7004 (2)	0.50306 (12)	0.40043 (11)	0.0422 (3)	
H6A	0.8278	0.5219	0.4251	0.051*	
H6B	0.6557	0.5085	0.4682	0.051*	
C21	0.64076 (16)	0.67242 (10)	0.14284 (10)	0.0311 (3)	
C22	0.74860 (16)	0.69609 (11)	0.06906 (11)	0.0342 (3)	
C23	0.71254 (19)	0.78402 (12)	−0.00441 (12)	0.0421 (3)	
H23	0.7837	0.7991	−0.0533	0.051*	
C24	0.5696 (2)	0.84922 (12)	−0.00446 (13)	0.0466 (4)	
H24	0.5458	0.9083	−0.0535	0.056*	
C25	0.4631 (2)	0.82783 (12)	0.06667 (13)	0.0476 (4)	
H25	0.3677	0.8721	0.0660	0.057*	
C26	0.49877 (18)	0.73919 (12)	0.14005 (12)	0.0399 (3)	
H26	0.4260	0.7245	0.1881	0.048*	
O22	0.88764 (13)	0.62807 (9)	0.07539 (9)	0.0471 (3)	
C27	1.0094 (2)	0.65658 (16)	0.01004 (16)	0.0601 (4)	
H27A	1.1050	0.6069	0.0266	0.090*	
H27B	1.0556	0.7372	0.0301	0.090*	
H27C	0.9495	0.6448	−0.0706	0.090*	
C31	1.07262 (16)	0.14162 (10)	0.58115 (10)	0.0328 (3)	
C32	1.23171 (17)	0.09894 (11)	0.57551 (11)	0.0361 (3)	
H32	1.2888	0.1233	0.5216	0.043*	
C33	1.30429 (17)	0.01961 (11)	0.65116 (12)	0.0384 (3)	
C34	1.22367 (18)	−0.02069 (11)	0.73110 (12)	0.0406 (3)	
H34	1.2719	−0.0765	0.7792	0.049*	
C35	1.06862 (18)	0.02555 (11)	0.73629 (11)	0.0376 (3)	
C36	0.99163 (17)	0.10646 (11)	0.66424 (11)	0.0356 (3)	
H36	0.8874	0.1369	0.6712	0.043*	
C37	0.98662 (19)	0.22652 (11)	0.49671 (11)	0.0402 (3)	
O31	0.84578 (15)	0.26458 (10)	0.51102 (9)	0.0585 (3)	
O32	1.05696 (16)	0.24991 (11)	0.42026 (10)	0.0655 (3)	
N33	1.47682 (18)	−0.02225 (14)	0.64945 (12)	0.0578 (4)	
O33	1.57628 (16)	0.04182 (15)	0.61300 (12)	0.0825 (4)	
O34	1.51295 (19)	−0.11651 (13)	0.68590 (15)	0.0890 (5)	
N35	0.98229 (19)	−0.01443 (13)	0.82304 (11)	0.0558 (4)	
O35	1.0381 (2)	−0.09880 (14)	0.87252 (13)	0.0929 (5)	
O36	0.86187 (19)	0.03867 (14)	0.84155 (11)	0.0794 (4)	
O41	0.28832 (16)	0.36547 (12)	0.31241 (12)	0.0639 (3)	
H41	0.225 (3)	0.322 (2)	0.341 (2)	0.096*	
H42	0.230 (3)	0.431 (2)	0.306 (2)	0.096*	
O51	0.13836 (19)	0.58148 (13)	0.29313 (13)	0.0722 (4)	
H51	0.152 (3)	0.630 (2)	0.356 (2)	0.087*	
H52	0.051 (3)	0.593 (2)	0.250 (2)	0.087*	

### 4-(2-Methoxyphenyl)piperazin-1-ium 3,5-dinitrobenzoate dihydrate (XI) Atomic displacement parameters (Å^2^)

**Table:**

	*U*^11^	*U*^22^	*U*^33^	*U*^12^	*U*^13^	*U*^23^
N1	0.0413 (6)	0.0353 (6)	0.0404 (6)	0.0049 (5)	0.0087 (5)	0.0154 (5)
C2	0.0584 (9)	0.0332 (7)	0.0429 (7)	0.0107 (6)	0.0162 (6)	0.0094 (6)
C3	0.0516 (8)	0.0308 (6)	0.0324 (6)	0.0051 (5)	0.0098 (6)	0.0057 (5)
N4	0.0371 (6)	0.0279 (5)	0.0311 (5)	0.0029 (4)	0.0096 (4)	0.0077 (4)
C5	0.0470 (8)	0.0315 (6)	0.0363 (7)	0.0019 (5)	0.0155 (6)	0.0049 (5)
C6	0.0536 (8)	0.0414 (7)	0.0320 (6)	0.0004 (6)	0.0118 (6)	0.0079 (5)
C21	0.0327 (6)	0.0270 (6)	0.0318 (6)	0.0005 (5)	0.0052 (5)	0.0060 (5)
C22	0.0338 (6)	0.0321 (6)	0.0365 (7)	0.0022 (5)	0.0077 (5)	0.0080 (5)
C23	0.0484 (8)	0.0401 (7)	0.0390 (7)	0.0001 (6)	0.0115 (6)	0.0141 (6)
C24	0.0540 (9)	0.0357 (7)	0.0460 (8)	0.0060 (6)	0.0022 (7)	0.0161 (6)
C25	0.0445 (8)	0.0382 (7)	0.0580 (9)	0.0130 (6)	0.0057 (7)	0.0111 (6)
C26	0.0375 (7)	0.0381 (7)	0.0459 (7)	0.0054 (5)	0.0125 (6)	0.0087 (6)
O22	0.0455 (6)	0.0506 (6)	0.0572 (6)	0.0148 (4)	0.0270 (5)	0.0240 (5)
C27	0.0554 (10)	0.0656 (10)	0.0750 (11)	0.0128 (8)	0.0392 (9)	0.0226 (9)
C31	0.0363 (7)	0.0269 (6)	0.0337 (6)	−0.0015 (5)	0.0072 (5)	0.0046 (5)
C32	0.0371 (7)	0.0359 (6)	0.0353 (6)	−0.0037 (5)	0.0113 (5)	0.0042 (5)
C33	0.0327 (7)	0.0377 (7)	0.0425 (7)	0.0049 (5)	0.0064 (5)	0.0007 (6)
C34	0.0440 (8)	0.0335 (7)	0.0413 (7)	0.0048 (5)	0.0038 (6)	0.0107 (6)
C35	0.0418 (7)	0.0367 (7)	0.0347 (7)	−0.0030 (5)	0.0107 (5)	0.0100 (5)
C36	0.0340 (7)	0.0360 (6)	0.0375 (7)	0.0032 (5)	0.0099 (5)	0.0070 (5)
C37	0.0465 (8)	0.0338 (7)	0.0375 (7)	−0.0006 (6)	0.0055 (6)	0.0098 (5)
O31	0.0658 (7)	0.0649 (7)	0.0502 (6)	0.0306 (6)	0.0137 (5)	0.0242 (5)
O32	0.0711 (8)	0.0732 (8)	0.0619 (7)	0.0061 (6)	0.0257 (6)	0.0399 (6)
N33	0.0409 (7)	0.0721 (10)	0.0583 (8)	0.0138 (7)	0.0086 (6)	0.0005 (7)
O33	0.0407 (7)	0.1309 (13)	0.0809 (9)	0.0122 (7)	0.0210 (6)	0.0204 (9)
O34	0.0697 (9)	0.0731 (9)	0.1238 (13)	0.0407 (7)	0.0150 (8)	0.0152 (8)
N35	0.0564 (8)	0.0689 (9)	0.0457 (7)	−0.0032 (7)	0.0170 (6)	0.0226 (6)
O35	0.1110 (12)	0.0950 (11)	0.0922 (10)	0.0168 (9)	0.0434 (9)	0.0664 (9)
O36	0.0691 (8)	0.1213 (12)	0.0645 (8)	0.0177 (8)	0.0387 (7)	0.0319 (8)
O41	0.0520 (7)	0.0584 (7)	0.0919 (9)	0.0049 (5)	0.0336 (6)	0.0231 (7)
O51	0.0665 (8)	0.0786 (9)	0.0674 (8)	0.0321 (7)	0.0051 (7)	0.0044 (7)

### 4-(2-Methoxyphenyl)piperazin-1-ium 3,5-dinitrobenzoate dihydrate (XI) Geometric parameters (Å, º)

**Table:**

N1—C2	1.4826 (18)	C26—H26	0.9300
N1—C6	1.4860 (18)	O22—C27	1.4257 (17)
N1—H11	0.929 (16)	C27—H27A	0.9600
N1—H12	0.908 (16)	C27—H27B	0.9600
C2—C3	1.5143 (17)	C27—H27C	0.9600
C2—H2A	0.9700	C31—C32	1.3856 (19)
C2—H2B	0.9700	C31—C36	1.3903 (17)
C3—N4	1.4694 (16)	C31—C37	1.5237 (17)
C3—H3A	0.9700	C32—C33	1.3820 (18)
C3—H3B	0.9700	C32—H32	0.9300
N4—C21	1.4300 (15)	C33—C34	1.3759 (19)
N4—C5	1.4638 (16)	C33—N33	1.4696 (19)
C5—C6	1.5086 (18)	C34—C35	1.372 (2)
C5—H5A	0.9700	C34—H34	0.9300
C5—H5B	0.9700	C35—C36	1.3803 (17)
C6—H6A	0.9700	C35—N35	1.4727 (17)
C6—H6B	0.9700	C36—H36	0.9300
C21—C26	1.3866 (18)	C37—O32	1.2297 (17)
C21—C22	1.4080 (17)	C37—O31	1.2615 (18)
C22—O22	1.3709 (16)	N33—O34	1.2204 (19)
C22—C23	1.3881 (17)	N33—O33	1.2231 (19)
C23—C24	1.387 (2)	N35—O36	1.2150 (19)
C23—H23	0.9300	N35—O35	1.2214 (18)
C24—C25	1.369 (2)	O41—H41	0.84 (2)
C24—H24	0.9300	O41—H42	0.91 (3)
C25—C26	1.3936 (19)	O51—H51	0.88 (2)
C25—H25	0.9300	O51—H52	0.77 (2)
			
C2—N1—C6	110.76 (10)	C23—C24—H24	119.5
C2—N1—H11	110.5 (10)	C24—C25—C26	119.49 (13)
C6—N1—H11	108.2 (9)	C24—C25—H25	120.3
C2—N1—H12	113.0 (10)	C26—C25—H25	120.3
C6—N1—H12	106.6 (10)	C21—C26—C25	121.23 (13)
H11—N1—H12	107.6 (13)	C21—C26—H26	119.4
N1—C2—C3	110.82 (11)	C25—C26—H26	119.4
N1—C2—H2A	109.5	C22—O22—C27	117.66 (11)
C3—C2—H2A	109.5	O22—C27—H27A	109.5
N1—C2—H2B	109.5	O22—C27—H27B	109.5
C3—C2—H2B	109.5	H27A—C27—H27B	109.5
H2A—C2—H2B	108.1	O22—C27—H27C	109.5
N4—C3—C2	110.21 (10)	H27A—C27—H27C	109.5
N4—C3—H3A	109.6	H27B—C27—H27C	109.5
C2—C3—H3A	109.6	C32—C31—C36	119.65 (11)
N4—C3—H3B	109.6	C32—C31—C37	120.23 (11)
C2—C3—H3B	109.6	C36—C31—C37	120.12 (12)
H3A—C3—H3B	108.1	C33—C32—C31	118.99 (12)
C21—N4—C5	115.30 (10)	C33—C32—H32	120.5
C21—N4—C3	112.57 (9)	C31—C32—H32	120.5
C5—N4—C3	109.48 (10)	C34—C33—C32	122.75 (12)
N4—C5—C6	109.39 (11)	C34—C33—N33	117.76 (12)
N4—C5—H5A	109.8	C32—C33—N33	119.48 (13)
C6—C5—H5A	109.8	C35—C34—C33	116.72 (12)
N4—C5—H5B	109.8	C35—C34—H34	121.6
C6—C5—H5B	109.8	C33—C34—H34	121.6
H5A—C5—H5B	108.2	C34—C35—C36	122.99 (12)
N1—C6—C5	110.28 (11)	C34—C35—N35	117.58 (12)
N1—C6—H6A	109.6	C36—C35—N35	119.42 (12)
C5—C6—H6A	109.6	C35—C36—C31	118.82 (12)
N1—C6—H6B	109.6	C35—C36—H36	120.6
C5—C6—H6B	109.6	C31—C36—H36	120.6
H6A—C6—H6B	108.1	O32—C37—O31	125.98 (13)
C26—C21—C22	118.27 (11)	O32—C37—C31	118.05 (13)
C26—C21—N4	123.70 (11)	O31—C37—C31	115.95 (12)
C22—C21—N4	118.02 (11)	O34—N33—O33	124.40 (15)
O22—C22—C23	123.48 (12)	O34—N33—C33	118.57 (15)
O22—C22—C21	116.04 (10)	O33—N33—C33	117.02 (14)
C23—C22—C21	120.48 (12)	O36—N35—O35	124.28 (14)
C24—C23—C22	119.57 (13)	O36—N35—C35	118.33 (13)
C24—C23—H23	120.2	O35—N35—C35	117.39 (15)
C22—C23—H23	120.2	H41—O41—H42	102 (2)
C25—C24—C23	120.95 (12)	H51—O51—H52	108 (2)
C25—C24—H24	119.5		
			
C6—N1—C2—C3	−54.19 (15)	C36—C31—C32—C33	1.65 (18)
N1—C2—C3—N4	56.54 (15)	C37—C31—C32—C33	−178.27 (11)
C2—C3—N4—C21	169.85 (11)	C31—C32—C33—C34	1.13 (19)
C2—C3—N4—C5	−60.51 (14)	C31—C32—C33—N33	−177.30 (12)
C21—N4—C5—C6	−169.80 (10)	C32—C33—C34—C35	−2.7 (2)
C3—N4—C5—C6	62.06 (13)	N33—C33—C34—C35	175.77 (12)
C2—N1—C6—C5	55.87 (15)	C33—C34—C35—C36	1.5 (2)
N4—C5—C6—N1	−59.79 (14)	C33—C34—C35—N35	−178.66 (12)
C5—N4—C21—C26	−21.15 (17)	C34—C35—C36—C31	1.1 (2)
C3—N4—C21—C26	105.44 (14)	N35—C35—C36—C31	−178.69 (12)
C5—N4—C21—C22	157.91 (11)	C32—C31—C36—C35	−2.72 (18)
C3—N4—C21—C22	−75.51 (14)	C37—C31—C36—C35	177.21 (11)
C26—C21—C22—O22	179.73 (11)	C32—C31—C37—O32	4.20 (18)
N4—C21—C22—O22	0.62 (16)	C36—C31—C37—O32	−175.73 (13)
C26—C21—C22—C23	−0.10 (18)	C32—C31—C37—O31	−177.24 (13)
N4—C21—C22—C23	−179.21 (11)	C36—C31—C37—O31	2.83 (18)
O22—C22—C23—C24	−179.50 (13)	C34—C33—N33—O34	25.3 (2)
C21—C22—C23—C24	0.3 (2)	C32—C33—N33—O34	−156.20 (15)
C22—C23—C24—C25	−0.2 (2)	C34—C33—N33—O33	−153.41 (14)
C23—C24—C25—C26	−0.1 (2)	C32—C33—N33—O33	25.1 (2)
C22—C21—C26—C25	−0.20 (19)	C34—C35—N35—O36	168.36 (14)
N4—C21—C26—C25	178.85 (12)	C36—C35—N35—O36	−11.8 (2)
C24—C25—C26—C21	0.3 (2)	C34—C35—N35—O35	−11.0 (2)
C23—C22—O22—C27	5.6 (2)	C36—C35—N35—O35	168.77 (14)
C21—C22—O22—C27	−174.26 (12)		

### 4-(2-Methoxyphenyl)piperazin-1-ium 3,5-dinitrobenzoate dihydrate (XI) Hydrogen-bond geometry (Å, º)

**Table:**

*D*—H···*A*	*D*—H	H···*A*	*D*···*A*	*D*—H···*A*
N1—H11···O31	0.929 (16)	1.771 (16)	2.6837 (16)	166.8 (15)
N1—H12···O41	0.911 (16)	1.939 (16)	2.8324 (19)	165.5 (14)
O41—H41···O32^i^	0.84 (2)	1.99 (2)	2.8156 (19)	168 (2)
O41—H42···O51	0.90 (2)	1.91 (2)	2.810 (2)	172 (2)
O51—H51···O31^ii^	0.90 (2)	1.91 (2)	2.810 (2)	172 (2)
O51—H52···O22^i^	0.77 (2)	2.25 (2)	2.9544 (19)	153 (2)
C25—H25···O36^ii^	0.93	2.58	3.433 (2)	153

Symmetry codes: (i) *x*−1, *y*, *z*; (ii) −*x*+1, −*y*+1, −*z*+1.

### 4-(2-Methoxyphenyl)piperazin-1-ium 2,4,6-trinitrophenolate (XII) Crystal data

**Table:**

C_11_H_17_N_2_O^+^·C_6_H_2_N_3_O_7_^−^	*Z* = 2
*M**_r_* = 421.33	*F*(000) = 440
Triclinic, *P*1	*D*_x_ = 1.435 Mg m^−^^3^
*a* = 9.4151 (5) Å	Mo *K*α radiation, λ = 0.71073 Å
*b* = 9.8721 (5) Å	Cell parameters from 4279 reflections
*c* = 10.9572 (5) Å	θ = 2.9–27.8°
α = 77.524 (4)°	µ = 0.12 mm^−^^1^
β = 81.360 (5)°	*T* = 296 K
γ = 81.002 (5)°	Plate, orange
*V* = 974.97 (9) Å^3^	0.48 × 0.48 × 0.24 mm

### 4-(2-Methoxyphenyl)piperazin-1-ium 2,4,6-trinitrophenolate (XII) Data collection

**Table:**

Oxford Diffraction Xcalibur with Sapphire CCD diffractometer	4279 independent reflections
Radiation source: Enhance (Mo) X-ray Source	3276 reflections with *I* > 2σ(*I*)
Graphite monochromator	*R*_int_ = 0.017
ω scans	θ_max_ = 27.8°, θ_min_ = 2.9°
Absorption correction: multi-scan (CrysAlis RED; Oxford Diffraction, 2009)	*h* = −12→12
*T*_min_ = 0.805, *T*_max_ = 0.973	*k* = −12→12
12926 measured reflections	*l* = −13→13

### 4-(2-Methoxyphenyl)piperazin-1-ium 2,4,6-trinitrophenolate (XII) Refinement

**Table:**

Refinement on *F*^2^	Hydrogen site location: mixed
Least-squares matrix: full	H atoms treated by a mixture of independent and constrained refinement
*R*[*F*^2^ > 2σ(*F*^2^)] = 0.040	*w* = 1/[σ^2^(*F*_o_^2^) + (0.0587*P*)^2^ + 0.1566*P*] where *P* = (*F*_o_^2^ + 2*F*_c_^2^)/3
*wR*(*F*^2^) = 0.119	(Δ/σ)_max_ < 0.001
*S* = 1.07	Δρ_max_ = 0.24 e Å^−^^3^
4279 reflections	Δρ_min_ = −0.27 e Å^−^^3^
317 parameters	Extinction correction: SHELXL, Fc^*^=kFc[1+0.001xFc^2^λ^3^/sin(2θ)]^-1/4^
85 restraints	Extinction coefficient: 0.021 (3)
Primary atom site location: difference Fourier map	

### 4-(2-Methoxyphenyl)piperazin-1-ium 2,4,6-trinitrophenolate (XII) Special details

**Table:**

Experimental. Empirical absorption correction using spherical harmonics, implemented in SCALE3 ABSPACK scaling algorithm.
Geometry. All esds (except the esd in the dihedral angle between two l.s. planes) are estimated using the full covariance matrix. The cell esds are taken into account individually in the estimation of esds in distances, angles and torsion angles; correlations between esds in cell parameters are only used when they are defined by crystal symmetry. An approximate (isotropic) treatment of cell esds is used for estimating esds involving l.s. planes.

### 4-(2-Methoxyphenyl)piperazin-1-ium 2,4,6-trinitrophenolate (XII) Fractional atomic coordinates and isotropic or equivalent isotropic displacement parameters (Å^2^)

**Table:**

	*x*	*y*	*z*	*U*_iso_*/*U*_eq_	Occ. (<1)
N1	0.55402 (16)	0.61143 (13)	0.19946 (11)	0.0462 (3)	
H11	0.480 (2)	0.5879 (18)	0.2530 (16)	0.055*	
H12	0.6047 (19)	0.6597 (18)	0.2347 (16)	0.055*	
C2	0.64890 (19)	0.48431 (16)	0.17048 (14)	0.0534 (4)	
H2A	0.5924	0.4256	0.1420	0.064*	
H2B	0.6880	0.4312	0.2461	0.064*	
C3	0.77123 (17)	0.52432 (16)	0.07001 (13)	0.0497 (4)	
H3A	0.8326	0.5761	0.1013	0.060*	
H3B	0.8296	0.4405	0.0492	0.060*	
N4	0.71457 (12)	0.61034 (12)	−0.04332 (10)	0.0418 (3)	
C5	0.62911 (16)	0.73798 (15)	−0.01273 (13)	0.0457 (3)	
H5A	0.5947	0.7967	−0.0883	0.055*	
H5B	0.6891	0.7898	0.0201	0.055*	
C6	0.50188 (16)	0.70175 (17)	0.08392 (14)	0.0490 (4)	
H6A	0.4457	0.7868	0.1043	0.059*	
H6B	0.4398	0.6532	0.0499	0.059*	
C21	0.82215 (14)	0.63009 (17)	−0.14998 (13)	0.0446 (3)	
C22	0.87678 (16)	0.51580 (19)	−0.20905 (14)	0.0525 (4)	
C23	0.97820 (18)	0.5326 (2)	−0.31534 (16)	0.0665 (5)	
H23	1.0151	0.4568	−0.3534	0.080*	
C24	1.02430 (19)	0.6610 (3)	−0.36469 (17)	0.0741 (6)	
H24	1.0912	0.6719	−0.4366	0.089*	
C25	0.97236 (19)	0.7728 (2)	−0.30852 (19)	0.0706 (5)	
H25	1.0036	0.8594	−0.3426	0.085*	
C26	0.87250 (17)	0.75717 (19)	−0.19997 (15)	0.0556 (4)	
H26	0.8396	0.8330	−0.1610	0.067*	
O22	0.82269 (13)	0.39384 (13)	−0.15675 (12)	0.0685 (4)	
C27	0.8927 (2)	0.2687 (2)	−0.1938 (2)	0.0859 (7)	
H27A	0.8424	0.1918	−0.1492	0.129*	
H27B	0.8921	0.2773	−0.2827	0.129*	
H27C	0.9909	0.2521	−0.1747	0.129*	
C31	0.29075 (13)	0.15664 (13)	0.64803 (12)	0.0362 (3)	
O31	0.27727 (12)	0.22396 (11)	0.73386 (9)	0.0512 (3)	
C32	0.32774 (14)	0.20872 (13)	0.51527 (12)	0.0361 (3)	
N32	0.35214 (13)	0.35445 (12)	0.47084 (11)	0.0445 (3)	
O32	0.3202 (2)	0.43611 (12)	0.54139 (13)	0.0911 (5)	
O33	0.40574 (16)	0.38892 (13)	0.36249 (11)	0.0705 (4)	
C33	0.34703 (14)	0.12760 (14)	0.42520 (12)	0.0381 (3)	
H33	0.3709	0.1669	0.3409	0.046*	
C34	0.33077 (14)	−0.01230 (14)	0.46070 (13)	0.0393 (3)	
N34	0.35475 (14)	−0.09763 (14)	0.36584 (13)	0.0501 (3)	
O34	0.38287 (16)	−0.04191 (13)	0.25521 (11)	0.0731 (4)	
O35	0.34933 (15)	−0.22433 (12)	0.40011 (12)	0.0699 (4)	
C35	0.29789 (14)	−0.07370 (14)	0.58638 (13)	0.0413 (3)	
H35	0.2907	−0.1689	0.6101	0.050*	
C36	0.27649 (15)	0.00877 (14)	0.67410 (13)	0.0404 (3)	
N36	0.24422 (18)	−0.05867 (14)	0.80615 (13)	0.0590 (4)	0.850 (5)
O36	0.3154 (3)	−0.1672 (2)	0.84426 (18)	0.0830 (8)	0.850 (5)
O37	0.1395 (3)	−0.0058 (3)	0.8665 (2)	0.1155 (12)	0.850 (5)
N37	0.24422 (18)	−0.05867 (14)	0.80615 (13)	0.0590 (4)	0.069 (4)
O38	0.1277 (16)	−0.090 (3)	0.853 (2)	0.094 (5)	0.069 (4)
O39	0.3392 (18)	−0.077 (3)	0.8764 (17)	0.081 (5)	0.069 (4)
N38	0.24422 (18)	−0.05867 (14)	0.80615 (13)	0.0590 (4)	0.080 (4)
O310	0.219 (3)	−0.1764 (12)	0.8390 (16)	0.074 (4)	0.080 (4)
O311	0.275 (3)	−0.0034 (19)	0.8882 (12)	0.084 (5)	0.080 (4)

### 4-(2-Methoxyphenyl)piperazin-1-ium 2,4,6-trinitrophenolate (XII) Atomic displacement parameters (Å^2^)

**Table:**

	*U*^11^	*U*^22^	*U*^33^	*U*^12^	*U*^13^	*U*^23^
N1	0.0592 (8)	0.0466 (7)	0.0358 (6)	−0.0182 (6)	0.0091 (5)	−0.0164 (5)
C2	0.0759 (11)	0.0414 (8)	0.0405 (8)	−0.0091 (7)	0.0051 (7)	−0.0101 (6)
C3	0.0557 (9)	0.0483 (8)	0.0401 (8)	0.0020 (7)	−0.0023 (6)	−0.0066 (6)
N4	0.0405 (6)	0.0504 (7)	0.0326 (6)	−0.0034 (5)	0.0021 (5)	−0.0102 (5)
C5	0.0490 (8)	0.0473 (8)	0.0384 (7)	−0.0022 (6)	−0.0017 (6)	−0.0083 (6)
C6	0.0478 (8)	0.0562 (9)	0.0443 (8)	−0.0028 (7)	0.0012 (6)	−0.0200 (7)
C21	0.0335 (7)	0.0627 (9)	0.0359 (7)	−0.0058 (6)	−0.0026 (5)	−0.0075 (6)
C22	0.0393 (7)	0.0748 (11)	0.0445 (8)	−0.0083 (7)	0.0029 (6)	−0.0190 (7)
C23	0.0459 (9)	0.1026 (15)	0.0524 (9)	−0.0098 (9)	0.0095 (7)	−0.0289 (10)
C24	0.0457 (9)	0.1197 (18)	0.0492 (10)	−0.0162 (10)	0.0118 (7)	−0.0081 (11)
C25	0.0446 (9)	0.0875 (14)	0.0697 (11)	−0.0201 (9)	0.0023 (8)	0.0086 (10)
C26	0.0427 (8)	0.0673 (10)	0.0532 (9)	−0.0103 (7)	−0.0022 (7)	−0.0040 (8)
O22	0.0631 (7)	0.0668 (8)	0.0754 (8)	−0.0114 (6)	0.0235 (6)	−0.0334 (6)
C27	0.0654 (12)	0.0845 (14)	0.1171 (18)	−0.0103 (10)	0.0152 (12)	−0.0576 (13)
C31	0.0339 (6)	0.0377 (7)	0.0377 (7)	−0.0051 (5)	−0.0007 (5)	−0.0108 (5)
O31	0.0628 (7)	0.0526 (6)	0.0424 (5)	−0.0143 (5)	0.0048 (5)	−0.0213 (5)
C32	0.0350 (6)	0.0334 (6)	0.0394 (7)	−0.0052 (5)	−0.0022 (5)	−0.0074 (5)
N32	0.0503 (7)	0.0376 (6)	0.0439 (7)	−0.0078 (5)	−0.0004 (5)	−0.0063 (5)
O32	0.1554 (15)	0.0395 (6)	0.0721 (9)	−0.0210 (8)	0.0280 (9)	−0.0198 (6)
O33	0.1042 (10)	0.0583 (7)	0.0468 (6)	−0.0345 (7)	0.0120 (6)	−0.0028 (5)
C33	0.0364 (6)	0.0435 (7)	0.0342 (6)	−0.0030 (5)	−0.0035 (5)	−0.0092 (5)
C34	0.0364 (7)	0.0417 (7)	0.0434 (7)	−0.0035 (5)	−0.0048 (5)	−0.0177 (6)
N34	0.0508 (7)	0.0505 (7)	0.0550 (8)	−0.0020 (6)	−0.0089 (6)	−0.0249 (6)
O34	0.1083 (11)	0.0670 (8)	0.0462 (7)	−0.0009 (7)	−0.0053 (6)	−0.0262 (6)
O35	0.0909 (9)	0.0500 (7)	0.0782 (8)	−0.0137 (6)	−0.0071 (7)	−0.0319 (6)
C35	0.0401 (7)	0.0345 (7)	0.0500 (8)	−0.0062 (5)	−0.0032 (6)	−0.0102 (6)
C36	0.0411 (7)	0.0395 (7)	0.0382 (7)	−0.0061 (6)	0.0004 (5)	−0.0050 (5)
N36	0.0847 (11)	0.0435 (8)	0.0447 (8)	−0.0127 (7)	0.0056 (7)	−0.0060 (6)
O36	0.1144 (18)	0.0577 (12)	0.0612 (10)	0.0080 (12)	−0.0148 (11)	0.0118 (8)
O37	0.153 (2)	0.0750 (15)	0.0736 (12)	0.0185 (14)	0.0626 (14)	0.0073 (10)
N37	0.0847 (11)	0.0435 (8)	0.0447 (8)	−0.0127 (7)	0.0056 (7)	−0.0060 (6)
O38	0.116 (10)	0.076 (9)	0.079 (9)	−0.015 (9)	0.010 (9)	−0.003 (9)
O39	0.095 (9)	0.072 (9)	0.051 (8)	0.024 (8)	−0.021 (7)	0.025 (8)
N38	0.0847 (11)	0.0435 (8)	0.0447 (8)	−0.0127 (7)	0.0056 (7)	−0.0060 (6)
O310	0.119 (10)	0.042 (7)	0.056 (7)	−0.011 (8)	−0.004 (8)	−0.001 (6)
O311	0.148 (10)	0.055 (8)	0.045 (7)	−0.019 (8)	0.014 (8)	−0.015 (6)

### 4-(2-Methoxyphenyl)piperazin-1-ium 2,4,6-trinitrophenolate (XII) Geometric parameters (Å, º)

**Table:**

N1—C6	1.484 (2)	C25—C26	1.397 (2)
N1—C2	1.4866 (19)	C25—H25	0.9300
N1—H11	0.869 (18)	C26—H26	0.9300
N1—H12	0.900 (19)	O22—C27	1.417 (2)
C2—C3	1.506 (2)	C27—H27A	0.9600
C2—H2A	0.9700	C27—H27B	0.9600
C2—H2B	0.9700	C27—H27C	0.9600
C3—N4	1.4661 (18)	C31—O31	1.2450 (15)
C3—H3A	0.9700	C31—C32	1.4430 (18)
C3—H3B	0.9700	C31—C36	1.4492 (19)
N4—C21	1.4274 (17)	C32—C33	1.3749 (18)
N4—C5	1.4601 (18)	C32—N32	1.4583 (17)
C5—C6	1.508 (2)	N32—O32	1.2089 (16)
C5—H5A	0.9700	N32—O33	1.2161 (16)
C5—H5B	0.9700	C33—C34	1.3777 (19)
C6—H6A	0.9700	C33—H33	0.9300
C6—H6B	0.9700	C34—C35	1.3892 (19)
C21—C26	1.382 (2)	C34—N34	1.4454 (17)
C21—C22	1.411 (2)	N34—O34	1.2260 (17)
C22—O22	1.358 (2)	N34—O35	1.2326 (17)
C22—C23	1.388 (2)	C35—C36	1.3623 (19)
C23—C24	1.376 (3)	C35—H35	0.9300
C23—H23	0.9300	C36—N36	1.4643 (19)
C24—C25	1.367 (3)	N36—O36	1.199 (2)
C24—H24	0.9300	N36—O37	1.214 (2)
			
C6—N1—C2	111.24 (11)	C25—C24—H24	119.8
C6—N1—H11	109.0 (11)	C23—C24—H24	119.8
C2—N1—H11	110.2 (11)	C24—C25—C26	120.14 (18)
C6—N1—H12	109.4 (11)	C24—C25—H25	119.9
C2—N1—H12	108.7 (11)	C26—C25—H25	119.9
H11—N1—H12	108.3 (15)	C21—C26—C25	120.64 (18)
N1—C2—C3	110.41 (12)	C21—C26—H26	119.7
N1—C2—H2A	109.6	C25—C26—H26	119.7
C3—C2—H2A	109.6	C22—O22—C27	119.05 (13)
N1—C2—H2B	109.6	O22—C27—H27A	109.5
C3—C2—H2B	109.6	O22—C27—H27B	109.5
H2A—C2—H2B	108.1	H27A—C27—H27B	109.5
N4—C3—C2	110.51 (13)	O22—C27—H27C	109.5
N4—C3—H3A	109.5	H27A—C27—H27C	109.5
C2—C3—H3A	109.5	H27B—C27—H27C	109.5
N4—C3—H3B	109.5	O31—C31—C32	126.64 (12)
C2—C3—H3B	109.5	O31—C31—C36	121.75 (12)
H3A—C3—H3B	108.1	C32—C31—C36	111.56 (11)
C21—N4—C5	115.52 (11)	C33—C32—C31	123.98 (12)
C21—N4—C3	113.44 (11)	C33—C32—N32	116.28 (11)
C5—N4—C3	109.59 (11)	C31—C32—N32	119.70 (11)
N4—C5—C6	109.95 (12)	O32—N32—O33	122.28 (13)
N4—C5—H5A	109.7	O32—N32—C32	119.94 (12)
C6—C5—H5A	109.7	O33—N32—C32	117.78 (12)
N4—C5—H5B	109.7	C32—C33—C34	119.57 (12)
C6—C5—H5B	109.7	C32—C33—H33	120.2
H5A—C5—H5B	108.2	C34—C33—H33	120.2
N1—C6—C5	109.85 (12)	C33—C34—C35	121.15 (12)
N1—C6—H6A	109.7	C33—C34—N34	119.23 (12)
C5—C6—H6A	109.7	C35—C34—N34	119.56 (12)
N1—C6—H6B	109.7	O34—N34—O35	122.70 (13)
C5—C6—H6B	109.7	O34—N34—C34	118.86 (13)
H6A—C6—H6B	108.2	O35—N34—C34	118.41 (13)
C26—C21—C22	118.49 (13)	C36—C35—C34	118.47 (12)
C26—C21—N4	122.91 (14)	C36—C35—H35	120.8
C22—C21—N4	118.58 (13)	C34—C35—H35	120.8
O22—C22—C23	123.92 (16)	C35—C36—C31	125.23 (12)
O22—C22—C21	116.04 (12)	C35—C36—N36	117.48 (12)
C23—C22—C21	120.03 (16)	C31—C36—N36	117.20 (12)
C24—C23—C22	120.29 (18)	O36—N36—O37	124.53 (18)
C24—C23—H23	119.9	O36—N36—C36	118.10 (16)
C22—C23—H23	119.9	O37—N36—C36	117.11 (16)
C25—C24—C23	120.37 (16)		
			
C6—N1—C2—C3	54.43 (18)	O31—C31—C32—N32	−0.5 (2)
N1—C2—C3—N4	−56.58 (17)	C36—C31—C32—N32	−177.72 (11)
C2—C3—N4—C21	−168.88 (12)	C33—C32—N32—O32	170.90 (15)
C2—C3—N4—C5	60.37 (16)	C31—C32—N32—O32	−11.3 (2)
C21—N4—C5—C6	168.83 (12)	C33—C32—N32—O33	−9.62 (19)
C3—N4—C5—C6	−61.53 (15)	C31—C32—N32—O33	168.23 (13)
C2—N1—C6—C5	−55.55 (16)	C31—C32—C33—C34	0.0 (2)
N4—C5—C6—N1	59.07 (15)	N32—C32—C33—C34	177.78 (11)
C5—N4—C21—C26	17.82 (19)	C32—C33—C34—C35	−1.2 (2)
C3—N4—C21—C26	−109.92 (16)	C32—C33—C34—N34	−178.48 (12)
C5—N4—C21—C22	−160.45 (13)	C33—C34—N34—O34	−3.1 (2)
C3—N4—C21—C22	71.82 (17)	C35—C34—N34—O34	179.59 (14)
C26—C21—C22—O22	−179.42 (14)	C33—C34—N34—O35	175.20 (13)
N4—C21—C22—O22	−1.1 (2)	C35—C34—N34—O35	−2.1 (2)
C26—C21—C22—C23	−0.3 (2)	C33—C34—C35—C36	2.4 (2)
N4—C21—C22—C23	178.00 (14)	N34—C34—C35—C36	179.67 (12)
O22—C22—C23—C24	178.03 (17)	C34—C35—C36—C31	−2.6 (2)
C21—C22—C23—C24	−1.0 (3)	C34—C35—C36—N36	−179.06 (13)
C22—C23—C24—C25	1.0 (3)	O31—C31—C36—C35	−176.00 (13)
C23—C24—C25—C26	0.4 (3)	C32—C31—C36—C35	1.36 (19)
C22—C21—C26—C25	1.7 (2)	O31—C31—C36—N36	0.5 (2)
N4—C21—C26—C25	−176.60 (14)	C32—C31—C36—N36	177.86 (12)
C24—C25—C26—C21	−1.7 (3)	C35—C36—N36—O36	44.8 (3)
C23—C22—O22—C27	13.9 (3)	C31—C36—N36—O36	−132.0 (2)
C21—C22—O22—C27	−167.10 (17)	C35—C36—N36—O37	−129.6 (3)
O31—C31—C32—C33	177.16 (13)	C31—C36—N36—O37	53.7 (3)
C36—C31—C32—C33	−0.05 (18)		

### 4-(2-Methoxyphenyl)piperazin-1-ium 2,4,6-trinitrophenolate (XII) Hydrogen-bond geometry (Å, º)

**Table:**

*D*—H···*A*	*D*—H	H···*A*	*D*···*A*	*D*—H···*A*
N1—H11···O33	0.868 (18)	2.224 (18)	2.9120 (19)	136.1 (16)
N1—H12···O31^i^	0.900 (18)	1.833 (18)	2.7142 (18)	165.9 (16)
N1—H12···O32^i^	0.900 (19)	2.593 (17)	3.154 (2)	121.2 (13)
C6—H6*A*···O34^ii^	0.97	2.56	3.423 (2)	148

Symmetry codes: (i) −*x*+1, −*y*+1, −*z*+1; (ii) *x*, *y*+1, *z*.

### 4-(2-Methoxyphenyl)piperazin-1-ium hydrogen maleate (XIII) Crystal data

**Table:**

C_11_H_17_N_2_O^+^·C_4_H_3_O_4_^−^	*Z* = 4
*M**_r_* = 308.33	*F*(000) = 656
Triclinic, *P*1	*D*_x_ = 1.287 Mg m^−^^3^
*a* = 11.1076 (6) Å	Mo *K*α radiation, λ = 0.71073 Å
*b* = 11.1164 (6) Å	Cell parameters from 6817 reflections
*c* = 13.7649 (7) Å	θ = 2.6–27.9°
α = 80.353 (5)°	µ = 0.10 mm^−^^1^
β = 78.353 (5)°	*T* = 296 K
γ = 74.406 (5)°	Block, orange
*V* = 1591.76 (16) Å^3^	0.48 × 0.40 × 0.36 mm

### 4-(2-Methoxyphenyl)piperazin-1-ium hydrogen maleate (XIII) Data collection

**Table:**

Oxford Diffraction Xcalibur with Sapphire CCD diffractometer	6817 independent reflections
Radiation source: Enhance (Mo) X-ray Source	4221 reflections with *I* > 2σ(*I*)
Graphite monochromator	*R*_int_ = 0.012
ω scans	θ_max_ = 27.9°, θ_min_ = 2.6°
Absorption correction: multi-scan (CrysAlis RED; Oxford Diffraction, 2009)	*h* = −14→11
*T*_min_ = 0.863, *T*_max_ = 0.966	*k* = −14→10
11727 measured reflections	*l* = −17→16

### 4-(2-Methoxyphenyl)piperazin-1-ium hydrogen maleate (XIII) Refinement

**Table:**

Refinement on *F*^2^	Primary atom site location: difference Fourier map
Least-squares matrix: full	Hydrogen site location: mixed
*R*[*F*^2^ > 2σ(*F*^2^)] = 0.042	H atoms treated by a mixture of independent and constrained refinement
*wR*(*F*^2^) = 0.121	*w* = 1/[σ^2^(*F*_o_^2^) + (0.0662*P*)^2^] where *P* = (*F*_o_^2^ + 2*F*_c_^2^)/3
*S* = 1.03	(Δ/σ)_max_ = 0.001
6817 reflections	Δρ_max_ = 0.15 e Å^−^^3^
415 parameters	Δρ_min_ = −0.16 e Å^−^^3^
0 restraints	

### 4-(2-Methoxyphenyl)piperazin-1-ium hydrogen maleate (XIII) Special details

**Table:**

Experimental. Empirical absorption correction using spherical harmonics, implemented in SCALE3 ABSPACK scaling algorithm.
Geometry. All esds (except the esd in the dihedral angle between two l.s. planes) are estimated using the full covariance matrix. The cell esds are taken into account individually in the estimation of esds in distances, angles and torsion angles; correlations between esds in cell parameters are only used when they are defined by crystal symmetry. An approximate (isotropic) treatment of cell esds is used for estimating esds involving l.s. planes.

### 4-(2-Methoxyphenyl)piperazin-1-ium hydrogen maleate (XIII) Fractional atomic coordinates and isotropic or equivalent isotropic displacement parameters (Å^2^)

**Table:**

	*x*	*y*	*z*	*U*_iso_*/*U*_eq_	
N11	0.34622 (12)	0.18907 (13)	0.37745 (11)	0.0623 (4)	
H111	0.2917 (16)	0.2421 (16)	0.4214 (12)	0.075*	
H112	0.3338 (15)	0.1085 (16)	0.3969 (11)	0.075*	
C12	0.47834 (15)	0.19077 (16)	0.38284 (12)	0.0642 (4)	
H12A	0.4969	0.1564	0.4493	0.077*	
H12B	0.4870	0.2768	0.3702	0.077*	
C13	0.57059 (14)	0.11410 (15)	0.30673 (11)	0.0570 (4)	
H13A	0.6560	0.1195	0.3080	0.068*	
H13B	0.5675	0.0265	0.3231	0.068*	
N14	0.53919 (10)	0.16040 (11)	0.20732 (8)	0.0467 (3)	
C15	0.41371 (12)	0.14755 (14)	0.20256 (12)	0.0562 (4)	
H15A	0.4102	0.0602	0.2201	0.067*	
H15B	0.3954	0.1744	0.1352	0.067*	
C16	0.31747 (14)	0.22734 (15)	0.27418 (13)	0.0616 (4)	
H16A	0.3182	0.3151	0.2542	0.074*	
H16B	0.2335	0.2182	0.2724	0.074*	
C121	0.63719 (12)	0.12322 (13)	0.12699 (10)	0.0455 (3)	
C122	0.74375 (13)	0.17360 (14)	0.10986 (11)	0.0516 (4)	
C123	0.83917 (14)	0.14329 (16)	0.02954 (12)	0.0636 (4)	
H123	0.9099	0.1764	0.0188	0.076*	
C124	0.83016 (15)	0.06460 (16)	−0.03458 (12)	0.0669 (5)	
H124	0.8946	0.0451	−0.0884	0.080*	
C125	0.72766 (15)	0.01536 (16)	−0.01957 (12)	0.0654 (4)	
H125	0.7218	−0.0375	−0.0632	0.078*	
C126	0.63143 (14)	0.04385 (14)	0.06097 (11)	0.0576 (4)	
H126	0.5618	0.0092	0.0709	0.069*	
O122	0.74475 (10)	0.25199 (11)	0.17621 (8)	0.0716 (3)	
C127	0.84336 (17)	0.31480 (17)	0.15906 (15)	0.0774 (5)	
H17A	0.8314	0.3660	0.2115	0.116*	
H17B	0.9234	0.2540	0.1580	0.116*	
H17C	0.8424	0.3671	0.0960	0.116*	
N21	0.32610 (11)	0.68791 (12)	0.35465 (9)	0.0506 (3)	
H211	0.2716 (14)	0.7345 (14)	0.4078 (11)	0.061*	
H212	0.3068 (13)	0.6135 (14)	0.3614 (11)	0.061*	
C22	0.45843 (14)	0.66671 (15)	0.37162 (11)	0.0559 (4)	
H22A	0.4692	0.6135	0.4343	0.067*	
H22B	0.4753	0.7466	0.3763	0.067*	
C23	0.55121 (13)	0.60521 (13)	0.28769 (10)	0.0510 (4)	
H23A	0.6371	0.5971	0.2979	0.061*	
H23B	0.5404	0.5216	0.2872	0.061*	
N24	0.53089 (10)	0.68033 (10)	0.19212 (8)	0.0443 (3)	
C25	0.40353 (12)	0.68965 (15)	0.17454 (11)	0.0548 (4)	
H25A	0.3914	0.6062	0.1757	0.066*	
H25B	0.3920	0.7357	0.1094	0.066*	
C26	0.30813 (13)	0.75658 (14)	0.25435 (11)	0.0549 (4)	
H26A	0.3179	0.8414	0.2509	0.066*	
H26B	0.2231	0.7622	0.2434	0.066*	
C221	0.63168 (12)	0.64911 (13)	0.11180 (10)	0.0454 (3)	
C222	0.74589 (13)	0.68107 (14)	0.11058 (11)	0.0505 (4)	
C223	0.84489 (15)	0.65473 (16)	0.03183 (13)	0.0660 (5)	
H223	0.9205	0.6760	0.0311	0.079*	
C224	0.83202 (17)	0.59725 (17)	−0.04533 (13)	0.0722 (5)	
H224	0.8991	0.5797	−0.0977	0.087*	
C225	0.72259 (17)	0.56616 (17)	−0.04537 (12)	0.0735 (5)	
H225	0.7143	0.5275	−0.0977	0.088*	
C226	0.62268 (16)	0.59194 (16)	0.03260 (11)	0.0641 (4)	
H226	0.5477	0.5703	0.0317	0.077*	
O222	0.75012 (10)	0.73834 (11)	0.18954 (8)	0.0683 (3)	
C227	0.85515 (17)	0.78893 (18)	0.18703 (14)	0.0806 (6)	
H27A	0.8449	0.8259	0.2473	0.121*	
H27B	0.9316	0.7230	0.1816	0.121*	
H27C	0.8601	0.8521	0.1305	0.121*	
C31	0.21393 (13)	0.42548 (13)	0.52990 (11)	0.0491 (3)	
O31	0.31437 (10)	0.44663 (10)	0.48375 (9)	0.0721 (3)	
O32	0.17455 (10)	0.33051 (9)	0.52076 (8)	0.0630 (3)	
C32	0.13422 (14)	0.51635 (13)	0.59942 (11)	0.0527 (4)	
H32	0.1710	0.5799	0.6060	0.063*	
C33	0.01974 (14)	0.52237 (13)	0.65358 (11)	0.0515 (4)	
H331	−0.0094	0.5890	0.6919	0.062*	
C34	−0.06961 (14)	0.44152 (13)	0.66358 (11)	0.0493 (4)	
O33	−0.03904 (10)	0.34388 (9)	0.61545 (8)	0.0587 (3)	
H33	0.0541 (18)	0.3376 (15)	0.5724 (13)	0.088*	
O34	−0.17286 (11)	0.46868 (11)	0.71631 (9)	0.0830 (4)	
C41	0.21753 (13)	0.92641 (13)	0.50681 (10)	0.0472 (3)	
O41	0.31284 (10)	0.95022 (9)	0.45083 (8)	0.0676 (3)	
O42	0.18296 (10)	0.82652 (9)	0.50841 (8)	0.0661 (3)	
C42	0.14234 (14)	1.02060 (13)	0.57458 (11)	0.0554 (4)	
H42	0.1724	1.0926	0.5676	0.067*	
C43	0.03981 (13)	1.01999 (13)	0.64339 (11)	0.0536 (4)	
H431	0.0104	1.0921	0.6756	0.064*	
C44	−0.03624 (14)	0.92597 (13)	0.67828 (11)	0.0525 (4)	
O43	−0.01234 (11)	0.82687 (10)	0.63191 (9)	0.0756 (4)	
H43	0.066 (2)	0.8219 (18)	0.5806 (15)	0.113*	
O44	−0.12061 (12)	0.94251 (11)	0.74866 (9)	0.0869 (4)	

### 4-(2-Methoxyphenyl)piperazin-1-ium hydrogen maleate (XIII) Atomic displacement parameters (Å^2^)

**Table:**

	*U*^11^	*U*^22^	*U*^33^	*U*^12^	*U*^13^	*U*^23^
N11	0.0566 (8)	0.0488 (8)	0.0713 (10)	−0.0164 (6)	0.0283 (7)	−0.0197 (7)
C12	0.0610 (10)	0.0759 (11)	0.0497 (9)	−0.0144 (8)	0.0086 (8)	−0.0156 (8)
C13	0.0480 (9)	0.0685 (10)	0.0474 (9)	−0.0093 (7)	0.0035 (7)	−0.0095 (7)
N14	0.0351 (6)	0.0597 (7)	0.0451 (7)	−0.0136 (5)	0.0027 (5)	−0.0138 (6)
C15	0.0391 (8)	0.0619 (9)	0.0705 (10)	−0.0165 (7)	0.0024 (7)	−0.0226 (8)
C16	0.0389 (8)	0.0622 (10)	0.0817 (12)	−0.0128 (7)	0.0077 (8)	−0.0233 (9)
C121	0.0390 (7)	0.0525 (8)	0.0417 (8)	−0.0080 (6)	−0.0006 (6)	−0.0092 (6)
C122	0.0445 (8)	0.0603 (9)	0.0483 (8)	−0.0159 (7)	0.0038 (7)	−0.0111 (7)
C123	0.0460 (9)	0.0804 (11)	0.0586 (10)	−0.0186 (8)	0.0103 (7)	−0.0097 (9)
C124	0.0546 (10)	0.0855 (12)	0.0474 (9)	−0.0003 (9)	0.0066 (7)	−0.0172 (9)
C125	0.0637 (11)	0.0742 (11)	0.0550 (10)	0.0003 (8)	−0.0073 (8)	−0.0296 (8)
C126	0.0500 (9)	0.0654 (10)	0.0600 (10)	−0.0121 (7)	−0.0065 (7)	−0.0211 (8)
O122	0.0621 (7)	0.0907 (8)	0.0735 (8)	−0.0425 (6)	0.0152 (6)	−0.0346 (7)
C127	0.0680 (11)	0.0796 (12)	0.0960 (14)	−0.0367 (10)	−0.0123 (10)	−0.0126 (10)
N21	0.0459 (7)	0.0451 (7)	0.0560 (8)	−0.0138 (6)	0.0135 (6)	−0.0152 (6)
C22	0.0519 (9)	0.0666 (10)	0.0432 (8)	−0.0120 (7)	0.0029 (7)	−0.0064 (7)
C23	0.0446 (8)	0.0558 (9)	0.0463 (8)	−0.0096 (7)	0.0019 (6)	−0.0049 (7)
N24	0.0362 (6)	0.0554 (7)	0.0402 (6)	−0.0131 (5)	0.0009 (5)	−0.0085 (5)
C25	0.0415 (8)	0.0714 (10)	0.0518 (9)	−0.0156 (7)	−0.0011 (7)	−0.0133 (7)
C26	0.0394 (8)	0.0578 (9)	0.0632 (10)	−0.0095 (7)	0.0004 (7)	−0.0103 (8)
C221	0.0416 (8)	0.0494 (8)	0.0431 (8)	−0.0128 (6)	0.0017 (6)	−0.0076 (6)
C222	0.0456 (8)	0.0556 (9)	0.0497 (9)	−0.0182 (7)	0.0020 (7)	−0.0063 (7)
C223	0.0464 (9)	0.0781 (11)	0.0677 (11)	−0.0225 (8)	0.0116 (8)	−0.0063 (9)
C224	0.0671 (11)	0.0813 (12)	0.0551 (10)	−0.0155 (9)	0.0207 (9)	−0.0132 (9)
C225	0.0785 (12)	0.0898 (13)	0.0520 (10)	−0.0218 (10)	0.0078 (9)	−0.0288 (9)
C226	0.0621 (10)	0.0839 (12)	0.0530 (10)	−0.0279 (9)	0.0021 (8)	−0.0242 (9)
O222	0.0581 (7)	0.0936 (8)	0.0664 (7)	−0.0409 (6)	0.0024 (5)	−0.0248 (6)
C227	0.0703 (12)	0.0969 (14)	0.0911 (14)	−0.0433 (10)	−0.0245 (10)	−0.0056 (11)
C31	0.0442 (8)	0.0477 (8)	0.0536 (9)	−0.0101 (6)	−0.0053 (7)	−0.0065 (7)
O31	0.0473 (6)	0.0693 (7)	0.0955 (9)	−0.0180 (5)	0.0097 (6)	−0.0181 (6)
O32	0.0595 (7)	0.0564 (6)	0.0738 (7)	−0.0217 (5)	0.0168 (5)	−0.0306 (5)
C32	0.0555 (9)	0.0422 (8)	0.0653 (10)	−0.0176 (7)	−0.0070 (8)	−0.0143 (7)
C33	0.0582 (9)	0.0408 (8)	0.0558 (9)	−0.0123 (7)	0.0004 (7)	−0.0180 (7)
C34	0.0545 (9)	0.0416 (8)	0.0479 (8)	−0.0125 (6)	0.0058 (7)	−0.0107 (6)
O33	0.0557 (6)	0.0550 (6)	0.0687 (7)	−0.0246 (5)	0.0121 (5)	−0.0255 (5)
O34	0.0722 (8)	0.0720 (8)	0.0981 (9)	−0.0285 (6)	0.0364 (7)	−0.0352 (7)
C41	0.0459 (8)	0.0441 (8)	0.0486 (8)	−0.0095 (6)	−0.0021 (7)	−0.0065 (6)
O41	0.0577 (7)	0.0555 (6)	0.0801 (8)	−0.0158 (5)	0.0177 (6)	−0.0139 (6)
O42	0.0704 (7)	0.0537 (6)	0.0733 (7)	−0.0257 (5)	0.0230 (6)	−0.0303 (5)
C42	0.0574 (9)	0.0424 (8)	0.0688 (10)	−0.0190 (7)	0.0029 (8)	−0.0180 (7)
C43	0.0555 (9)	0.0433 (8)	0.0620 (9)	−0.0114 (7)	0.0019 (7)	−0.0214 (7)
C44	0.0541 (9)	0.0486 (8)	0.0521 (9)	−0.0119 (7)	0.0051 (7)	−0.0161 (7)
O43	0.0816 (8)	0.0597 (7)	0.0857 (9)	−0.0368 (6)	0.0332 (7)	−0.0343 (6)
O44	0.0886 (9)	0.0869 (9)	0.0816 (9)	−0.0369 (7)	0.0373 (7)	−0.0372 (7)

### 4-(2-Methoxyphenyl)piperazin-1-ium hydrogen maleate (XIII) Geometric parameters (Å, º)

**Table:**

N11—C16	1.487 (2)	N24—C221	1.4199 (15)
N11—C12	1.490 (2)	N24—C25	1.4566 (17)
N11—H111	0.927 (17)	C25—C26	1.5045 (18)
N11—H112	0.930 (16)	C25—H25A	0.9700
C12—C13	1.5068 (19)	C25—H25B	0.9700
C12—H12A	0.9700	C26—H26A	0.9700
C12—H12B	0.9700	C26—H26B	0.9700
C13—N14	1.4556 (18)	C221—C226	1.383 (2)
C13—H13A	0.9700	C221—C222	1.4027 (18)
C13—H13B	0.9700	C222—O222	1.3629 (17)
N14—C121	1.4155 (16)	C222—C223	1.3863 (19)
N14—C15	1.4541 (16)	C223—C224	1.376 (2)
C15—C16	1.5031 (19)	C223—H223	0.9300
C15—H15A	0.9700	C224—C225	1.350 (2)
C15—H15B	0.9700	C224—H224	0.9300
C16—H16A	0.9700	C225—C226	1.384 (2)
C16—H16B	0.9700	C225—H225	0.9300
C121—C126	1.3896 (19)	C226—H226	0.9300
C121—C122	1.4034 (18)	O222—C227	1.4172 (17)
C122—O122	1.3673 (17)	C227—H27A	0.9600
C122—C123	1.3853 (19)	C227—H27B	0.9600
C123—C124	1.377 (2)	C227—H27C	0.9600
C123—H123	0.9300	C31—O31	1.2281 (15)
C124—C125	1.357 (2)	C31—O32	1.2793 (16)
C124—H124	0.9300	C31—C32	1.4904 (19)
C125—C126	1.3889 (19)	C32—C33	1.3287 (18)
C125—H125	0.9300	C32—H32	0.9300
C126—H126	0.9300	C33—C34	1.4814 (19)
O122—C127	1.4123 (16)	C33—H331	0.9300
C127—H17A	0.9600	C34—O34	1.2185 (16)
C127—H17B	0.9600	C34—O33	1.2977 (16)
C127—H17C	0.9600	O33—H33	1.074 (19)
N21—C22	1.4848 (19)	C41—O41	1.2400 (15)
N21—C26	1.4849 (19)	C41—O42	1.2643 (15)
N21—H211	0.974 (16)	C41—C42	1.4855 (19)
N21—H212	0.894 (14)	C42—C43	1.3269 (18)
C22—C23	1.5064 (18)	C42—H42	0.9300
C22—H22A	0.9700	C43—C44	1.4762 (19)
C22—H22B	0.9700	C43—H431	0.9300
C23—N24	1.4592 (17)	C44—O44	1.2046 (16)
C23—H23A	0.9700	C44—O43	1.3044 (16)
C23—H23B	0.9700	O43—H43	1.00 (2)
			
C16—N11—C12	111.87 (11)	C22—C23—H23A	109.6
C16—N11—H111	111.2 (11)	N24—C23—H23B	109.6
C12—N11—H111	107.9 (10)	C22—C23—H23B	109.6
C16—N11—H112	106.9 (10)	H23A—C23—H23B	108.1
C12—N11—H112	110.6 (10)	C221—N24—C25	116.82 (11)
H111—N11—H112	108.4 (13)	C221—N24—C23	114.50 (11)
N11—C12—C13	110.06 (13)	C25—N24—C23	110.09 (10)
N11—C12—H12A	109.6	N24—C25—C26	109.32 (12)
C13—C12—H12A	109.6	N24—C25—H25A	109.8
N11—C12—H12B	109.6	C26—C25—H25A	109.8
C13—C12—H12B	109.6	N24—C25—H25B	109.8
H12A—C12—H12B	108.2	C26—C25—H25B	109.8
N14—C13—C12	110.14 (12)	H25A—C25—H25B	108.3
N14—C13—H13A	109.6	N21—C26—C25	110.30 (12)
C12—C13—H13A	109.6	N21—C26—H26A	109.6
N14—C13—H13B	109.6	C25—C26—H26A	109.6
C12—C13—H13B	109.6	N21—C26—H26B	109.6
H13A—C13—H13B	108.1	C25—C26—H26B	109.6
C121—N14—C15	117.32 (11)	H26A—C26—H26B	108.1
C121—N14—C13	115.49 (11)	C226—C221—C222	117.72 (12)
C15—N14—C13	110.45 (11)	C226—C221—N24	123.68 (12)
N14—C15—C16	108.98 (12)	C222—C221—N24	118.57 (12)
N14—C15—H15A	109.9	O222—C222—C223	124.36 (13)
C16—C15—H15A	109.9	O222—C222—C221	115.74 (12)
N14—C15—H15B	109.9	C223—C222—C221	119.90 (14)
C16—C15—H15B	109.9	C224—C223—C222	120.46 (14)
H15A—C15—H15B	108.3	C224—C223—H223	119.8
N11—C16—C15	110.40 (13)	C222—C223—H223	119.8
N11—C16—H16A	109.6	C225—C224—C223	120.36 (14)
C15—C16—H16A	109.6	C225—C224—H224	119.8
N11—C16—H16B	109.6	C223—C224—H224	119.8
C15—C16—H16B	109.6	C224—C225—C226	119.96 (15)
H16A—C16—H16B	108.1	C224—C225—H225	120.0
C126—C121—C122	117.83 (12)	C226—C225—H225	120.0
C126—C121—N14	123.65 (12)	C221—C226—C225	121.60 (14)
C122—C121—N14	118.45 (12)	C221—C226—H226	119.2
O122—C122—C123	124.19 (13)	C225—C226—H226	119.2
O122—C122—C121	115.76 (11)	C222—O222—C227	119.00 (12)
C123—C122—C121	120.05 (14)	O222—C227—H27A	109.5
C124—C123—C122	120.56 (14)	O222—C227—H27B	109.5
C124—C123—H123	119.7	H27A—C227—H27B	109.5
C122—C123—H123	119.7	O222—C227—H27C	109.5
C125—C124—C123	120.29 (14)	H27A—C227—H27C	109.5
C125—C124—H124	119.9	H27B—C227—H27C	109.5
C123—C124—H124	119.9	O31—C31—O32	123.42 (13)
C124—C125—C126	120.03 (14)	O31—C31—C32	117.74 (13)
C124—C125—H125	120.0	O32—C31—C32	118.84 (12)
C126—C125—H125	120.0	C31—O32—H33	111.0 (7)
C125—C126—C121	121.23 (14)	C33—C32—C31	131.17 (13)
C125—C126—H126	119.4	C33—C32—H32	114.4
C121—C126—H126	119.4	C31—C32—H32	114.4
C122—O122—C127	119.05 (12)	C32—C33—C34	131.27 (13)
O122—C127—H17A	109.5	C32—C33—H331	114.4
O122—C127—H17B	109.5	C34—C33—H331	114.4
H17A—C127—H17B	109.5	O34—C34—O33	120.32 (13)
O122—C127—H17C	109.5	O34—C34—C33	119.31 (13)
H17A—C127—H17C	109.5	O33—C34—C33	120.37 (12)
H17B—C127—H17C	109.5	C34—O33—H33	108.8 (9)
C22—N21—C26	111.73 (11)	O41—C41—O42	122.92 (13)
C22—N21—H211	106.8 (8)	O41—C41—C42	117.54 (12)
C26—N21—H211	111.6 (9)	O42—C41—C42	119.54 (12)
C22—N21—H212	108.9 (10)	C41—O42—H43	112.1 (8)
C26—N21—H212	110.2 (10)	C43—C42—C41	131.25 (13)
H211—N21—H212	107.5 (12)	C43—C42—H42	114.4
N21—C22—C23	110.82 (12)	C41—C42—H42	114.4
N21—C22—H22A	109.5	C42—C43—C44	131.86 (13)
C23—C22—H22A	109.5	C42—C43—H431	114.1
N21—C22—H22B	109.5	C44—C43—H431	114.1
C23—C22—H22B	109.5	O44—C44—O43	120.81 (13)
H22A—C22—H22B	108.1	O44—C44—C43	119.49 (13)
N24—C23—C22	110.15 (12)	O43—C44—C43	119.69 (12)
N24—C23—H23A	109.6	C44—O43—H43	111.0 (11)
			
C16—N11—C12—C13	−52.90 (17)	C23—N24—C25—C26	62.57 (15)
N11—C12—C13—N14	56.09 (17)	C22—N21—C26—C25	54.13 (15)
C12—C13—N14—C121	161.93 (12)	N24—C25—C26—N21	−58.61 (15)
C12—C13—N14—C15	−61.98 (15)	C25—N24—C221—C226	−20.0 (2)
C121—N14—C15—C16	−162.19 (12)	C23—N24—C221—C226	110.76 (16)
C13—N14—C15—C16	62.61 (16)	C25—N24—C221—C222	157.98 (13)
C12—N11—C16—C15	54.36 (16)	C23—N24—C221—C222	−71.22 (16)
N14—C15—C16—N11	−58.23 (16)	C226—C221—C222—O222	179.36 (13)
C15—N14—C121—C126	−19.4 (2)	N24—C221—C222—O222	1.2 (2)
C13—N14—C121—C126	113.61 (16)	C226—C221—C222—C223	−0.2 (2)
C15—N14—C121—C122	157.54 (13)	N24—C221—C222—C223	−178.34 (13)
C13—N14—C121—C122	−69.47 (16)	O222—C222—C223—C224	−179.56 (15)
C126—C121—C122—O122	179.46 (13)	C221—C222—C223—C224	0.0 (2)
N14—C121—C122—O122	2.4 (2)	C222—C223—C224—C225	0.2 (3)
C126—C121—C122—C123	−0.3 (2)	C223—C224—C225—C226	−0.1 (3)
N14—C121—C122—C123	−177.42 (14)	C222—C221—C226—C225	0.3 (2)
O122—C122—C123—C124	−179.23 (15)	N24—C221—C226—C225	178.33 (15)
C121—C122—C123—C124	0.5 (2)	C224—C225—C226—C221	−0.2 (3)
C122—C123—C124—C125	−0.2 (3)	C223—C222—O222—C227	8.1 (2)
C123—C124—C125—C126	−0.2 (3)	C221—C222—O222—C227	−171.41 (14)
C124—C125—C126—C121	0.5 (2)	O31—C31—C32—C33	175.34 (16)
C122—C121—C126—C125	−0.2 (2)	O32—C31—C32—C33	−4.3 (3)
N14—C121—C126—C125	176.77 (14)	C31—C32—C33—C34	−0.5 (3)
C123—C122—O122—C127	5.1 (2)	C32—C33—C34—O34	−177.55 (16)
C121—C122—O122—C127	−174.63 (14)	C32—C33—C34—O33	1.4 (3)
C26—N21—C22—C23	−52.60 (16)	O41—C41—C42—C43	−177.97 (16)
N21—C22—C23—N24	55.64 (15)	O42—C41—C42—C43	1.8 (3)
C22—C23—N24—C221	164.83 (11)	C41—C42—C43—C44	0.5 (3)
C22—C23—N24—C25	−61.17 (14)	C42—C43—C44—O44	173.80 (18)
C221—N24—C25—C26	−164.61 (11)	C42—C43—C44—O43	−7.0 (3)

### 4-(2-Methoxyphenyl)piperazin-1-ium hydrogen maleate (XIII) Hydrogen-bond geometry (Å, º)

**Table:**

*D*—H···*A*	*D*—H	H···*A*	*D*···*A*	*D*—H···*A*
O33—H33···O32	1.07 (2)	1.37 (2)	2.4447 (16)	177.7 (16)
O43—H43···O42	1.00 (2)	1.48 (2)	2.4707 (17)	174.0 (17)
N11—H111···O32	0.927 (17)	1.891 (17)	2.8122 (18)	172.3 (16)
N11—H112···O41^i^	0.930 (17)	1.848 (17)	2.7725 (17)	172.9 (13)
N21—H211···O42	0.975 (15)	1.821 (15)	2.7926 (16)	174.5 (14)
N21—H212···O31	0.895 (15)	2.283 (15)	2.9776 (17)	134.4 (12)
N21—H212···O34^ii^	0.895 (15)	2.428 (15)	3.1170 (18)	134.1 (12)
C16—H16*A*···O34^ii^	0.97	2.55	3.341 (2)	138
C16—H16*B*···O44^ii^	0.97	2.52	3.338 (2)	141
C25—H25*B*···*Cg*4^iii^	0.97	2.92	3.8440 (16)	159

Symmetry codes: (i) *x*, *y*−1, *z*; (ii) −*x*, −*y*+1, −*z*+1; (iii) −*x*+1, −*y*, −*z*.

### 4-(2-Methoxyphenyl)piperazin-1-ium hydrogen fumarate (XIV) Crystal data

**Table:**

C_11_H_17_N_2_O^+^·C_4_H_3_O_4_^−^	*Z* = 2
*M**_r_* = 308.33	*F*(000) = 328
Triclinic, *P*1	*D*_x_ = 1.326 Mg m^−^^3^
*a* = 7.8546 (4) Å	Mo *K*α radiation, λ = 0.71073 Å
*b* = 8.9626 (6) Å	Cell parameters from 3307 reflections
*c* = 11.2056 (8) Å	θ = 2.6–27.8°
α = 79.043 (5)°	µ = 0.10 mm^−^^1^
β = 87.715 (5)°	*T* = 296 K
γ = 85.840 (5)°	Block, colourless
*V* = 772.15 (9) Å^3^	0.48 × 0.48 × 0.34 mm

### 4-(2-Methoxyphenyl)piperazin-1-ium hydrogen fumarate (XIV) Data collection

**Table:**

Oxford Diffraction Xcalibur with Sapphire CCD diffractometer	3307 independent reflections
Radiation source: Enhance (Mo) X-ray Source	2608 reflections with *I* > 2σ(*I*)
Graphite monochromator	*R*_int_ = 0.009
ω scans	θ_max_ = 27.8°, θ_min_ = 2.6°
Absorption correction: multi-scan (CrysAlis RED; Oxford Diffraction, 2009)	*h* = −10→5
*T*_min_ = 0.867, *T*_max_ = 0.967	*k* = −11→11
5533 measured reflections	*l* = −13→14

### 4-(2-Methoxyphenyl)piperazin-1-ium hydrogen fumarate (XIV) Refinement

**Table:**

Refinement on *F*^2^	Hydrogen site location: mixed
Least-squares matrix: full	H-atom parameters constrained
*R*[*F*^2^ > 2σ(*F*^2^)] = 0.036	*w* = 1/[σ^2^(*F*_o_^2^) + (0.0552*P*)^2^ + 0.0881*P*] where *P* = (*F*_o_^2^ + 2*F*_c_^2^)/3
*wR*(*F*^2^) = 0.105	(Δ/σ)_max_ < 0.001
*S* = 1.06	Δρ_max_ = 0.20 e Å^−^^3^
3307 reflections	Δρ_min_ = −0.15 e Å^−^^3^
240 parameters	Extinction correction: SHELXL, Fc^*^=kFc[1+0.001xFc^2^λ^3^/sin(2θ)]^-1/4^
6 restraints	Extinction coefficient: 0.022 (4)
Primary atom site location: difference Fourier map	

### 4-(2-Methoxyphenyl)piperazin-1-ium hydrogen fumarate (XIV) Special details

**Table:**

Experimental. Empirical absorption correction using spherical harmonics, implemented in SCALE3 ABSPACK scaling algorithm.
Geometry. All esds (except the esd in the dihedral angle between two l.s. planes) are estimated using the full covariance matrix. The cell esds are taken into account individually in the estimation of esds in distances, angles and torsion angles; correlations between esds in cell parameters are only used when they are defined by crystal symmetry. An approximate (isotropic) treatment of cell esds is used for estimating esds involving l.s. planes.

### 4-(2-Methoxyphenyl)piperazin-1-ium hydrogen fumarate (XIV) Fractional atomic coordinates and isotropic or equivalent isotropic displacement parameters (Å^2^)

**Table:**

	*x*	*y*	*z*	*U*_iso_*/*U*_eq_	Occ. (<1)
N1	0.47840 (14)	0.33032 (13)	0.75754 (9)	0.0496 (3)	
H11	0.5555	0.3715	0.7951	0.059*	
H12	0.4236	0.2650	0.8131	0.059*	
C2	0.56542 (16)	0.24842 (16)	0.66646 (12)	0.0521 (3)	
H2A	0.6417	0.1661	0.7071	0.063*	
H2B	0.6330	0.3178	0.6099	0.063*	
C3	0.43454 (16)	0.18491 (14)	0.59804 (12)	0.0478 (3)	
H3A	0.4922	0.1350	0.5367	0.057*	
H3B	0.3740	0.1092	0.6540	0.057*	
N4	0.31104 (13)	0.30552 (11)	0.53920 (9)	0.0435 (2)	
C5	0.22526 (17)	0.38282 (15)	0.63102 (12)	0.0499 (3)	
H5A	0.1632	0.3105	0.6890	0.060*	
H5B	0.1438	0.4621	0.5923	0.060*	
C6	0.35447 (19)	0.45212 (16)	0.69650 (13)	0.0561 (3)	
H6A	0.4148	0.5262	0.6389	0.067*	
H6B	0.2966	0.5042	0.7565	0.067*	
C21	0.19938 (15)	0.25100 (13)	0.46154 (11)	0.0427 (3)	
C22	0.26637 (16)	0.21262 (14)	0.35255 (11)	0.0463 (3)	
C23	0.16107 (18)	0.15988 (17)	0.27531 (13)	0.0576 (3)	
H23	0.2063	0.1337	0.2035	0.069*	
C24	−0.01099 (19)	0.14591 (19)	0.30429 (15)	0.0649 (4)	
H24	−0.0806	0.1102	0.2521	0.078*	
C25	−0.07834 (18)	0.18457 (18)	0.40935 (15)	0.0644 (4)	
H25	−0.1940	0.1763	0.4283	0.077*	
C26	0.02619 (17)	0.23620 (15)	0.48775 (13)	0.0532 (3)	
H26	−0.0207	0.2615	0.5594	0.064*	
O22	0.43698 (12)	0.23087 (12)	0.32891 (8)	0.0587 (3)	
C27	0.50598 (18)	0.20342 (16)	0.21566 (12)	0.0541 (3)	
H27A	0.6256	0.2200	0.2105	0.081*	
H27B	0.4904	0.1001	0.2090	0.081*	
H27C	0.4486	0.2715	0.1508	0.081*	
C31	0.7592 (6)	0.4912 (5)	0.9702 (5)	0.0396 (11)	0.572 (9)
O31	0.7521 (6)	0.4322 (5)	0.8811 (5)	0.0587 (10)	0.572 (9)
O32	0.6295 (6)	0.5334 (6)	1.0339 (5)	0.0540 (9)	0.572 (9)
H32	0.5412	0.5112	1.0068	0.081*	0.286
C32	0.9263 (5)	0.5248 (3)	1.0179 (3)	0.0476 (11)	0.572 (9)
H32A	0.9320	0.6190	1.0756	0.057*	0.572 (9)
C34	0.7860 (7)	0.4556 (8)	0.9314 (7)	0.0410 (14)	0.428 (9)
O33	0.7520 (8)	0.4140 (9)	0.8394 (6)	0.0583 (12)	0.428 (9)
O34	0.6784 (8)	0.4985 (9)	1.0107 (5)	0.0557 (14)	0.428 (9)
H34	0.5809	0.4886	0.9913	0.084*	0.214
C33	0.9707 (5)	0.4644 (5)	0.9604 (4)	0.0472 (15)	0.428 (9)
H33A	1.0572	0.4552	0.8986	0.057*	0.428 (9)
C41	0.24159 (13)	0.01915 (13)	0.97848 (10)	0.0383 (3)	
O41	0.25523 (10)	0.12259 (12)	0.89098 (9)	0.0615 (3)	
O42	0.36567 (10)	−0.05149 (12)	1.04016 (8)	0.0574 (3)	
H42	0.4557	−0.0178	1.0112	0.086*	0.5
C42	0.07112 (13)	−0.03520 (14)	1.02179 (10)	0.0393 (3)	
H42A	0.0657	−0.1221	1.0818	0.047*	

### 4-(2-Methoxyphenyl)piperazin-1-ium hydrogen fumarate (XIV) Atomic displacement parameters (Å^2^)

**Table:**

	*U*^11^	*U*^22^	*U*^33^	*U*^12^	*U*^13^	*U*^23^
N1	0.0464 (6)	0.0605 (7)	0.0424 (5)	−0.0165 (5)	−0.0024 (4)	−0.0058 (5)
C2	0.0406 (7)	0.0675 (8)	0.0470 (7)	−0.0047 (6)	−0.0008 (5)	−0.0071 (6)
C3	0.0443 (7)	0.0494 (7)	0.0489 (7)	0.0028 (5)	−0.0057 (5)	−0.0081 (5)
N4	0.0452 (6)	0.0427 (5)	0.0427 (5)	−0.0007 (4)	−0.0064 (4)	−0.0076 (4)
C5	0.0524 (7)	0.0463 (7)	0.0513 (7)	0.0047 (5)	−0.0088 (6)	−0.0114 (5)
C6	0.0666 (9)	0.0500 (7)	0.0535 (8)	−0.0074 (6)	−0.0068 (6)	−0.0121 (6)
C21	0.0432 (6)	0.0374 (6)	0.0463 (6)	−0.0015 (5)	−0.0077 (5)	−0.0043 (5)
C22	0.0416 (6)	0.0481 (7)	0.0496 (7)	−0.0042 (5)	−0.0059 (5)	−0.0088 (5)
C23	0.0527 (8)	0.0692 (9)	0.0557 (8)	−0.0046 (6)	−0.0071 (6)	−0.0227 (7)
C24	0.0501 (8)	0.0785 (10)	0.0735 (10)	−0.0069 (7)	−0.0142 (7)	−0.0292 (8)
C25	0.0400 (7)	0.0780 (10)	0.0783 (10)	−0.0063 (7)	−0.0053 (7)	−0.0211 (8)
C26	0.0451 (7)	0.0573 (8)	0.0582 (8)	−0.0007 (6)	−0.0026 (6)	−0.0142 (6)
O22	0.0458 (5)	0.0834 (7)	0.0526 (5)	−0.0150 (5)	0.0024 (4)	−0.0235 (5)
C27	0.0530 (8)	0.0604 (8)	0.0467 (7)	−0.0043 (6)	0.0009 (6)	−0.0050 (6)
C31	0.035 (2)	0.0441 (19)	0.040 (3)	−0.0097 (16)	−0.0107 (19)	−0.0044 (15)
O31	0.0420 (13)	0.0858 (19)	0.060 (3)	−0.0113 (11)	−0.0172 (19)	−0.038 (2)
O32	0.0362 (19)	0.081 (2)	0.0480 (18)	−0.0149 (14)	−0.0069 (14)	−0.0143 (13)
C32	0.037 (2)	0.0607 (15)	0.0489 (17)	−0.0106 (11)	−0.0115 (12)	−0.0157 (12)
C34	0.033 (2)	0.053 (3)	0.039 (4)	−0.0108 (19)	−0.008 (2)	−0.011 (2)
O33	0.0441 (15)	0.086 (3)	0.054 (3)	−0.0188 (15)	−0.015 (2)	−0.028 (2)
O34	0.039 (4)	0.089 (4)	0.044 (3)	−0.017 (3)	−0.010 (2)	−0.0188 (19)
C33	0.033 (2)	0.068 (2)	0.044 (2)	−0.0125 (15)	−0.0099 (15)	−0.0141 (18)
C41	0.0237 (5)	0.0535 (7)	0.0386 (6)	−0.0081 (4)	0.0017 (4)	−0.0091 (5)
O41	0.0273 (4)	0.0793 (7)	0.0671 (6)	−0.0118 (4)	−0.0007 (4)	0.0162 (5)
O42	0.0222 (4)	0.0826 (7)	0.0590 (6)	−0.0101 (4)	−0.0031 (4)	0.0110 (5)
C42	0.0254 (5)	0.0516 (6)	0.0407 (6)	−0.0093 (4)	0.0010 (4)	−0.0058 (5)

### 4-(2-Methoxyphenyl)piperazin-1-ium hydrogen fumarate (XIV) Geometric parameters (Å, º)

**Table:**

N1—C2	1.4845 (17)	C24—H24	0.9300
N1—C6	1.4907 (17)	C25—C26	1.3883 (19)
N1—H11	0.8900	C25—H25	0.9300
N1—H12	0.8900	C26—H26	0.9300
C2—C3	1.5108 (18)	O22—C27	1.4175 (16)
C2—H2A	0.9700	C27—H27A	0.9600
C2—H2B	0.9700	C27—H27B	0.9600
C3—N4	1.4738 (15)	C27—H27C	0.9600
C3—H3A	0.9700	C31—O31	1.221 (4)
C3—H3B	0.9700	C31—O32	1.296 (5)
N4—C21	1.4318 (15)	C31—C32	1.508 (4)
N4—C5	1.4630 (16)	O32—H32	0.8200
C5—C6	1.5093 (18)	C32—H32A	1.1605
C5—H5A	0.9700	C34—O33	1.207 (6)
C5—H5B	0.9700	C34—O34	1.294 (6)
C6—H6A	0.9700	C34—C33	1.510 (5)
C6—H6B	0.9700	O34—H34	0.8200
C21—C26	1.3906 (18)	C33—H33A	0.9611
C21—C22	1.4033 (18)	C41—O41	1.2219 (14)
C22—O22	1.3710 (16)	C41—O42	1.2752 (14)
C22—C23	1.3877 (18)	C41—C42	1.4895 (14)
C23—C24	1.386 (2)	O42—H42	0.8200
C23—H23	0.9300	C42—C42^i^	1.307 (2)
C24—C25	1.365 (2)	C42—H42A	0.9300
			
C2—N1—C6	110.03 (10)	C23—C22—C21	120.06 (12)
C2—N1—H11	109.7	C24—C23—C22	120.53 (13)
C6—N1—H11	109.7	C24—C23—H23	119.7
C2—N1—H12	109.7	C22—C23—H23	119.7
C6—N1—H12	109.7	C25—C24—C23	120.04 (13)
H11—N1—H12	108.2	C25—C24—H24	120.0
N1—C2—C3	109.88 (10)	C23—C24—H24	120.0
N1—C2—H2A	109.7	C24—C25—C26	119.89 (13)
C3—C2—H2A	109.7	C24—C25—H25	120.1
N1—C2—H2B	109.7	C26—C25—H25	120.1
C3—C2—H2B	109.7	C25—C26—C21	121.48 (13)
H2A—C2—H2B	108.2	C25—C26—H26	119.3
N4—C3—C2	111.53 (10)	C21—C26—H26	119.3
N4—C3—H3A	109.3	C22—O22—C27	117.72 (10)
C2—C3—H3A	109.3	O22—C27—H27A	109.5
N4—C3—H3B	109.3	O22—C27—H27B	109.5
C2—C3—H3B	109.3	H27A—C27—H27B	109.5
H3A—C3—H3B	108.0	O22—C27—H27C	109.5
C21—N4—C5	115.02 (10)	H27A—C27—H27C	109.5
C21—N4—C3	112.31 (9)	H27B—C27—H27C	109.5
C5—N4—C3	109.65 (9)	O31—C31—O32	125.7 (4)
N4—C5—C6	110.19 (11)	O31—C31—C32	122.3 (5)
N4—C5—H5A	109.6	O32—C31—C32	112.0 (5)
C6—C5—H5A	109.6	C31—O32—H32	109.5
N4—C5—H5B	109.6	C31—C32—H32A	120.8
C6—C5—H5B	109.6	O33—C34—O34	126.6 (6)
H5A—C5—H5B	108.1	O33—C34—C33	119.4 (6)
N1—C6—C5	109.74 (11)	O34—C34—C33	114.0 (5)
N1—C6—H6A	109.7	C34—O34—H34	109.5
C5—C6—H6A	109.7	C34—C33—H33A	119.0
N1—C6—H6B	109.7	O41—C41—O42	125.06 (10)
C5—C6—H6B	109.7	O41—C41—C42	120.88 (10)
H6A—C6—H6B	108.2	O42—C41—C42	114.07 (10)
C26—C21—C22	118.00 (11)	C41—O42—H42	109.5
C26—C21—N4	123.21 (11)	C42^i^—C42—C41	122.20 (14)
C22—C21—N4	118.79 (11)	C42^i^—C42—H42A	118.9
O22—C22—C23	123.60 (12)	C41—C42—H42A	118.9
O22—C22—C21	116.34 (10)		
			
C6—N1—C2—C3	−56.53 (14)	C26—C21—C22—C23	−0.80 (19)
N1—C2—C3—N4	56.82 (14)	N4—C21—C22—C23	−179.82 (11)
C2—C3—N4—C21	172.63 (10)	O22—C22—C23—C24	−179.23 (14)
C2—C3—N4—C5	−58.17 (13)	C21—C22—C23—C24	0.6 (2)
C21—N4—C5—C6	−172.92 (10)	C22—C23—C24—C25	0.2 (2)
C3—N4—C5—C6	59.39 (13)	C23—C24—C25—C26	−0.7 (2)
C2—N1—C6—C5	58.42 (14)	C24—C25—C26—C21	0.5 (2)
N4—C5—C6—N1	−60.04 (14)	C22—C21—C26—C25	0.3 (2)
C5—N4—C21—C26	−15.65 (17)	N4—C21—C26—C25	179.22 (12)
C3—N4—C21—C26	110.69 (13)	C23—C22—O22—C27	4.19 (19)
C5—N4—C21—C22	163.31 (11)	C21—C22—O22—C27	−175.63 (11)
C3—N4—C21—C22	−70.35 (14)	O41—C41—C42—C42^i^	−8.0 (2)
C26—C21—C22—O22	179.03 (11)	O42—C41—C42—C42^i^	172.10 (15)
N4—C21—C22—O22	0.02 (17)		

Symmetry code: (i) −*x*, −*y*, −*z*+2.

### 4-(2-Methoxyphenyl)piperazin-1-ium hydrogen fumarate (XIV) Hydrogen-bond geometry (Å, º)

**Table:**

*D*—H···*A*	*D*—H	H···*A*	*D*···*A*	*D*—H···*A*
N1—H11···O31	0.89	2.01	2.894 (5)	171
N1—H11···O33	0.89	1.73	2.584 (7)	160
N1—H12···O41	0.89	1.97	2.8251 (15)	161
O32—H32···O32^ii^	0.82	1.54	2.355 (7)	176
O34—H34···O34^ii^	0.82	2.03	2.820 (9)	161
O42—H42···O42^iii^	0.82	1.62	2.4352 (12)	177

Symmetry codes: (ii) −*x*+1, −*y*+1, −*z*+2; (iii) −*x*+1, −*y*, −*z*+2.

### 4-(2-Methoxyphenyl)piperazin-1-ium hydrogen (2R,3R)-tartrate 1.698-hydrate (XV) Crystal data

**Table:**

C_11_H_17_N_2_O^+^·C_4_H_5_O_6_^−^·1.698H_2_O	*F*(000) = 398
*M**_r_* = 372.97	*D*_x_ = 1.345 Mg m^−^^3^
Monoclinic, *P*2_1_	Mo *K*α radiation, λ = 0.71073 Å
*a* = 7.479 (1) Å	Cell parameters from 2896 reflections
*b* = 7.065 (1) Å	θ = 3.1–27.9°
*c* = 17.788 (3) Å	µ = 0.11 mm^−^^1^
β = 101.58 (2)°	*T* = 296 K
*V* = 920.8 (2) Å^3^	Plate, colourless
*Z* = 2	0.36 × 0.32 × 0.12 mm

### 4-(2-Methoxyphenyl)piperazin-1-ium hydrogen (2R,3R)-tartrate 1.698-hydrate (XV) Data collection

**Table:**

Oxford Diffraction Xcalibur with Sapphire CCD diffractometer	2895 independent reflections
Radiation source: Enhance (Mo) X-ray Source	2062 reflections with *I* > 2σ(*I*)
Graphite monochromator	*R*_int_ = 0.022
ω scans	θ_max_ = 27.9°, θ_min_ = 3.1°
Absorption correction: multi-scan (CrysAlis RED; Oxford Diffraction, 2009)	*h* = −9→9
*T*_min_ = 0.956, *T*_max_ = 0.987	*k* = −9→6
3655 measured reflections	*l* = −23→22

### 4-(2-Methoxyphenyl)piperazin-1-ium hydrogen (2R,3R)-tartrate 1.698-hydrate (XV) Refinement

**Table:**

Refinement on *F*^2^	Primary atom site location: difference Fourier map
Least-squares matrix: full	Hydrogen site location: mixed
*R*[*F*^2^ > 2σ(*F*^2^)] = 0.039	H atoms treated by a mixture of independent and constrained refinement
*wR*(*F*^2^) = 0.081	*w* = 1/[σ^2^(*F*_o_^2^) + (0.0402*P*)^2^] where *P* = (*F*_o_^2^ + 2*F*_c_^2^)/3
*S* = 0.97	(Δ/σ)_max_ < 0.001
2895 reflections	Δρ_max_ = 0.14 e Å^−^^3^
263 parameters	Δρ_min_ = −0.17 e Å^−^^3^
4 restraints	Absolute structure: Flack *x* determined using 493 quotients [(*I*^+^)-(*I*^-^)]/[(*I*^+^)+(*I*^-^)] (Parsons *et al.*, 2013)

### 4-(2-Methoxyphenyl)piperazin-1-ium hydrogen (2R,3R)-tartrate 1.698-hydrate (XV) Special details

**Table:**

Experimental. Empirical absorption correction using spherical harmonics, implemented in SCALE3 ABSPACK scaling algorithm.
Geometry. All esds (except the esd in the dihedral angle between two l.s. planes) are estimated using the full covariance matrix. The cell esds are taken into account individually in the estimation of esds in distances, angles and torsion angles; correlations between esds in cell parameters are only used when they are defined by crystal symmetry. An approximate (isotropic) treatment of cell esds is used for estimating esds involving l.s. planes.

### 4-(2-Methoxyphenyl)piperazin-1-ium hydrogen (2R,3R)-tartrate 1.698-hydrate (XV) Fractional atomic coordinates and isotropic or equivalent isotropic displacement parameters (Å^2^)

**Table:**

	*x*	*y*	*z*	*U*_iso_*/*U*_eq_	Occ. (<1)
N1	0.4545 (5)	0.3626 (4)	0.31605 (16)	0.0472 (7)	
H11	0.517 (5)	0.295 (5)	0.3458 (19)	0.057*	
H12	0.341 (5)	0.371 (6)	0.3245 (18)	0.057*	
C2	0.4519 (5)	0.2806 (5)	0.23968 (19)	0.0514 (9)	
H2A	0.3910	0.1588	0.2356	0.062*	
H2B	0.5760	0.2611	0.2328	0.062*	
C3	0.3538 (5)	0.4111 (4)	0.17851 (18)	0.0454 (8)	
H3A	0.3539	0.3575	0.1283	0.055*	
H3B	0.2280	0.4258	0.1839	0.055*	
N4	0.4437 (3)	0.5952 (4)	0.18516 (13)	0.0393 (6)	
C5	0.4369 (5)	0.6791 (5)	0.25945 (16)	0.0441 (8)	
H5A	0.3106	0.6953	0.2639	0.053*	
H5B	0.4941	0.8029	0.2633	0.053*	
C6	0.5333 (5)	0.5553 (4)	0.32330 (19)	0.0521 (10)	
H6A	0.6621	0.5488	0.3217	0.063*	
H6B	0.5219	0.6093	0.3723	0.063*	
C21	0.3879 (5)	0.7242 (5)	0.12346 (16)	0.0416 (8)	
C22	0.5099 (5)	0.8649 (5)	0.11148 (18)	0.0515 (9)	
C23	0.4638 (6)	0.9900 (6)	0.0511 (2)	0.0665 (11)	
H23	0.5453	1.0842	0.0435	0.080*	
C24	0.2984 (7)	0.9750 (7)	0.0025 (2)	0.0749 (13)	
H24	0.2678	1.0590	−0.0383	0.090*	
C25	0.1785 (6)	0.8393 (7)	0.0131 (2)	0.0695 (12)	
H25	0.0662	0.8300	−0.0205	0.083*	
C26	0.2227 (5)	0.7140 (6)	0.07395 (18)	0.0546 (9)	
H26	0.1390	0.6219	0.0812	0.066*	
O22	0.6727 (4)	0.8666 (4)	0.16178 (15)	0.0723 (8)	
C27	0.8158 (6)	0.9858 (8)	0.1471 (3)	0.0936 (15)	
H27A	0.9212	0.9712	0.1875	0.140*	
H27B	0.8463	0.9511	0.0990	0.140*	
H27C	0.7762	1.1153	0.1449	0.140*	
C31	0.8601 (4)	0.0130 (4)	0.38558 (16)	0.0321 (7)	
O31	0.8264 (3)	0.1842 (3)	0.36938 (12)	0.0422 (5)	
O32	0.7484 (3)	−0.1056 (3)	0.39964 (13)	0.0484 (6)	
C32	1.0532 (4)	−0.0580 (4)	0.38922 (16)	0.0300 (7)	
H32A	1.0927	−0.0187	0.3423	0.036*	
O33	1.0645 (3)	−0.2564 (3)	0.39473 (13)	0.0431 (6)	
H33	0.985 (5)	−0.285 (6)	0.414 (2)	0.065*	
C33	1.1809 (3)	0.0309 (4)	0.45784 (14)	0.0292 (7)	
H33A	1.1800	0.1684	0.4506	0.035*	
O34	1.1191 (3)	−0.0087 (3)	0.52580 (11)	0.0405 (5)	
H34	1.162 (5)	−0.109 (6)	0.544 (2)	0.061*	
C34	1.3725 (4)	−0.0400 (4)	0.46138 (16)	0.0307 (7)	
O35	1.4553 (3)	−0.1271 (3)	0.51524 (11)	0.0479 (6)	
O36	1.4319 (3)	−0.0004 (3)	0.39858 (11)	0.0351 (5)	
H36	1.537 (5)	−0.036 (6)	0.4062 (17)	0.053*	
O41	0.0854 (4)	0.4055 (4)	0.31999 (16)	0.0612 (8)	
H41	0.068 (6)	0.512 (7)	0.336 (2)	0.092*	
H42	−0.004 (6)	0.335 (7)	0.329 (2)	0.092*	
O51	−0.1304 (7)	0.4856 (9)	0.1775 (2)	0.104 (2)	0.698 (9)
H51	−0.051 (9)	0.470 (13)	0.228 (2)	0.157*	0.698 (9)
H52	−0.225 (8)	0.573 (10)	0.185 (4)	0.157*	0.698 (9)

### 4-(2-Methoxyphenyl)piperazin-1-ium hydrogen (2R,3R)-tartrate 1.698-hydrate (XV) Atomic displacement parameters (Å^2^)

**Table:**

	*U*^11^	*U*^22^	*U*^33^	*U*^12^	*U*^13^	*U*^23^
N1	0.0576 (19)	0.0355 (16)	0.0460 (17)	−0.0002 (16)	0.0043 (15)	0.0093 (14)
C2	0.063 (2)	0.0322 (18)	0.061 (2)	0.0033 (17)	0.0179 (18)	0.0013 (16)
C3	0.057 (2)	0.0362 (19)	0.0443 (18)	−0.0014 (17)	0.0143 (15)	−0.0036 (14)
N4	0.0462 (16)	0.0352 (14)	0.0382 (14)	0.0025 (13)	0.0127 (12)	0.0025 (12)
C5	0.060 (2)	0.0325 (17)	0.0389 (17)	−0.0028 (17)	0.0080 (16)	0.0031 (15)
C6	0.065 (2)	0.040 (2)	0.0468 (18)	−0.0102 (18)	−0.0007 (17)	0.0083 (15)
C21	0.053 (2)	0.0404 (19)	0.0355 (16)	0.0091 (16)	0.0186 (15)	0.0017 (15)
C22	0.064 (2)	0.047 (2)	0.0475 (19)	0.002 (2)	0.0208 (17)	0.0074 (18)
C23	0.093 (3)	0.054 (2)	0.062 (2)	0.005 (2)	0.037 (2)	0.015 (2)
C24	0.104 (4)	0.067 (3)	0.058 (2)	0.024 (3)	0.026 (2)	0.026 (2)
C25	0.074 (3)	0.077 (3)	0.053 (2)	0.018 (3)	0.003 (2)	0.009 (2)
C26	0.056 (2)	0.057 (2)	0.0502 (19)	0.009 (2)	0.0081 (17)	0.0031 (19)
O22	0.0621 (16)	0.0759 (19)	0.0786 (17)	−0.0177 (16)	0.0137 (14)	0.0213 (16)
C27	0.078 (3)	0.091 (3)	0.118 (4)	−0.030 (3)	0.034 (3)	0.010 (3)
C31	0.0235 (14)	0.0325 (18)	0.0411 (15)	0.0001 (14)	0.0084 (12)	−0.0038 (14)
O31	0.0316 (11)	0.0350 (13)	0.0616 (13)	0.0062 (10)	0.0129 (10)	0.0057 (11)
O32	0.0248 (10)	0.0384 (13)	0.0869 (16)	−0.0026 (10)	0.0224 (10)	0.0027 (12)
C32	0.0268 (15)	0.0240 (15)	0.0419 (15)	0.0009 (12)	0.0130 (12)	−0.0027 (13)
O33	0.0312 (12)	0.0296 (12)	0.0749 (16)	−0.0031 (10)	0.0256 (11)	−0.0055 (11)
C33	0.0243 (14)	0.0284 (16)	0.0364 (15)	0.0027 (12)	0.0098 (12)	0.0044 (13)
O34	0.0407 (12)	0.0439 (14)	0.0424 (11)	0.0089 (11)	0.0212 (10)	0.0037 (11)
C34	0.0244 (14)	0.0311 (15)	0.0368 (15)	−0.0018 (13)	0.0063 (13)	−0.0025 (14)
O35	0.0388 (12)	0.0597 (15)	0.0442 (12)	0.0126 (12)	0.0058 (10)	0.0128 (12)
O36	0.0200 (9)	0.0436 (13)	0.0435 (11)	0.0026 (10)	0.0104 (9)	0.0042 (10)
O41	0.0671 (17)	0.0429 (16)	0.0847 (18)	−0.0022 (14)	0.0415 (14)	−0.0044 (13)
O51	0.104 (4)	0.137 (5)	0.072 (3)	0.042 (4)	0.020 (2)	0.004 (3)

### 4-(2-Methoxyphenyl)piperazin-1-ium hydrogen (2R,3R)-tartrate 1.698-hydrate (XV) Geometric parameters (Å, º)

**Table:**

N1—C2	1.473 (4)	C25—C26	1.386 (5)
N1—C6	1.479 (4)	C25—H25	0.9300
N1—H11	0.79 (4)	C26—H26	0.9300
N1—H12	0.90 (4)	O22—C27	1.427 (5)
C2—C3	1.500 (4)	C27—H27A	0.9600
C2—H2A	0.9700	C27—H27B	0.9600
C2—H2B	0.9700	C27—H27C	0.9600
C3—N4	1.458 (4)	C31—O32	1.244 (4)
C3—H3A	0.9700	C31—O31	1.257 (4)
C3—H3B	0.9700	C31—C32	1.518 (4)
N4—C21	1.423 (4)	C32—O33	1.406 (3)
N4—C5	1.458 (4)	C32—C33	1.526 (4)
C5—C6	1.499 (4)	C32—H32A	0.9800
C5—H5A	0.9700	O33—H33	0.78 (4)
C5—H5B	0.9700	C33—O34	1.406 (3)
C6—H6A	0.9700	C33—C34	1.508 (4)
C6—H6B	0.9700	C33—H33A	0.9800
C21—C26	1.368 (4)	O34—H34	0.82 (4)
C21—C22	1.394 (5)	C34—O35	1.201 (3)
C22—O22	1.359 (4)	C34—O36	1.312 (3)
C22—C23	1.381 (5)	O36—H36	0.81 (3)
C23—C24	1.363 (6)	O41—H41	0.83 (5)
C23—H23	0.9300	O41—H42	0.88 (5)
C24—C25	1.352 (6)	O51—H51	0.98 (2)
C24—H24	0.9300	O51—H52	0.97 (2)
			
C2—N1—C6	111.9 (3)	C22—C23—H23	120.1
C2—N1—H11	106 (2)	C25—C24—C23	120.7 (4)
C6—N1—H11	109 (3)	C25—C24—H24	119.7
C2—N1—H12	110 (2)	C23—C24—H24	119.7
C6—N1—H12	107 (3)	C24—C25—C26	120.0 (4)
H11—N1—H12	112 (4)	C24—C25—H25	120.0
N1—C2—C3	109.9 (3)	C26—C25—H25	120.0
N1—C2—H2A	109.7	C21—C26—C25	120.9 (4)
C3—C2—H2A	109.7	C21—C26—H26	119.6
N1—C2—H2B	109.7	C25—C26—H26	119.6
C3—C2—H2B	109.7	C22—O22—C27	119.4 (3)
H2A—C2—H2B	108.2	O22—C27—H27A	109.5
N4—C3—C2	109.8 (3)	O22—C27—H27B	109.5
N4—C3—H3A	109.7	H27A—C27—H27B	109.5
C2—C3—H3A	109.7	O22—C27—H27C	109.5
N4—C3—H3B	109.7	H27A—C27—H27C	109.5
C2—C3—H3B	109.7	H27B—C27—H27C	109.5
H3A—C3—H3B	108.2	O32—C31—O31	125.6 (3)
C21—N4—C3	116.7 (2)	O32—C31—C32	116.1 (2)
C21—N4—C5	112.4 (2)	O31—C31—C32	118.2 (3)
C3—N4—C5	109.7 (2)	O33—C32—C31	112.1 (2)
N4—C5—C6	110.5 (3)	O33—C32—C33	109.6 (2)
N4—C5—H5A	109.5	C31—C32—C33	109.6 (2)
C6—C5—H5A	109.5	O33—C32—H32A	108.5
N4—C5—H5B	109.5	C31—C32—H32A	108.5
C6—C5—H5B	109.5	C33—C32—H32A	108.5
H5A—C5—H5B	108.1	C32—O33—H33	105 (3)
N1—C6—C5	110.4 (3)	O34—C33—C34	111.8 (2)
N1—C6—H6A	109.6	O34—C33—C32	110.2 (2)
C5—C6—H6A	109.6	C34—C33—C32	109.5 (2)
N1—C6—H6B	109.6	O34—C33—H33A	108.4
C5—C6—H6B	109.6	C34—C33—H33A	108.4
H6A—C6—H6B	108.1	C32—C33—H33A	108.4
C26—C21—C22	118.2 (3)	C33—O34—H34	110 (3)
C26—C21—N4	123.4 (3)	O35—C34—O36	125.5 (3)
C22—C21—N4	118.3 (3)	O35—C34—C33	122.5 (3)
O22—C22—C23	123.9 (4)	O36—C34—C33	112.0 (2)
O22—C22—C21	115.7 (3)	C34—O36—H36	106 (2)
C23—C22—C21	120.5 (3)	H41—O41—H42	106 (4)
C24—C23—C22	119.7 (4)	H51—O51—H52	106 (3)
C24—C23—H23	120.1		
			
C6—N1—C2—C3	−54.9 (4)	C23—C24—C25—C26	0.3 (6)
N1—C2—C3—N4	58.6 (4)	C22—C21—C26—C25	0.6 (5)
C2—C3—N4—C21	169.1 (3)	N4—C21—C26—C25	−177.5 (3)
C2—C3—N4—C5	−61.7 (3)	C24—C25—C26—C21	−0.7 (6)
C21—N4—C5—C6	−167.9 (3)	C23—C22—O22—C27	−8.7 (6)
C3—N4—C5—C6	60.6 (3)	C21—C22—O22—C27	170.4 (4)
C2—N1—C6—C5	53.7 (4)	O32—C31—C32—O33	10.6 (4)
N4—C5—C6—N1	−56.1 (4)	O31—C31—C32—O33	−169.8 (2)
C3—N4—C21—C26	22.6 (4)	O32—C31—C32—C33	−111.3 (3)
C5—N4—C21—C26	−105.3 (3)	O31—C31—C32—C33	68.3 (3)
C3—N4—C21—C22	−155.5 (3)	O33—C32—C33—O34	−66.8 (3)
C5—N4—C21—C22	76.6 (3)	C31—C32—C33—O34	56.6 (3)
C26—C21—C22—O22	−179.1 (3)	O33—C32—C33—C34	56.6 (3)
N4—C21—C22—O22	−0.9 (4)	C31—C32—C33—C34	179.9 (2)
C26—C21—C22—C23	0.0 (5)	O34—C33—C34—O35	3.2 (4)
N4—C21—C22—C23	178.2 (3)	C32—C33—C34—O35	−119.2 (3)
O22—C22—C23—C24	178.6 (4)	O34—C33—C34—O36	−178.9 (3)
C21—C22—C23—C24	−0.4 (5)	C32—C33—C34—O36	58.7 (3)
C22—C23—C24—C25	0.3 (6)		

### 4-(2-Methoxyphenyl)piperazin-1-ium hydrogen (2R,3R)-tartrate 1.698-hydrate (XV) Hydrogen-bond geometry (Å, º)

**Table:**

*D*—H···*A*	*D*—H	H···*A*	*D*···*A*	*D*—H···*A*
N1—H11···O31	0.79 (4)	2.40 (4)	3.028 (4)	137 (3)
N1—H11···O36^i^	0.79 (4)	2.43 (4)	2.977 (4)	128 (3)
N1—H11···O35^ii^	0.79 (4)	2.50 (3)	2.942 (3)	117 (3)
N1—H12···O41	0.89 (4)	1.91 (4)	2.792 (5)	168 (3)
O33—H33···O34^iii^	0.77 (4)	2.14 (4)	2.800 (3)	144 (4)
O34—H34···O31^iii^	0.82 (4)	2.11 (4)	2.836 (3)	148 (3)
O36—H36···O32^iv^	0.81 (4)	1.68 (4)	2.478 (3)	167 (3)
O41—H41···O33^v^	0.82 (5)	1.94 (5)	2.753 (4)	167 (3)
O41—H42···O31^i^	0.87 (5)	1.90 (5)	2.766 (4)	169 (3)
O51—H51···O41	0.98 (4)	1.80 (5)	2.776 (5)	172 (9)
O51—H52···O22^i^	0.97 (7)	2.22 (7)	3.054 (7)	144 (6)
O51—H52···N4^i^	0.97 (7)	2.48 (6)	3.307 (6)	143 (5)
C23—H23···*Cg*2^vi^	0.93	2.91	3.722 (4)	147

Symmetry codes: (i) *x*−1, *y*, *z*; (ii) −*x*+2, *y*+1/2, −*z*+1; (iii) −*x*+2, *y*−1/2, −*z*+1; (iv) *x*+1, *y*, *z*; (v) *x*−1, *y*+1, *z*; (vi) −*x*+1, *y*+1/2, −*z*.
